# A New 1,5-Disubstituted Triazole DNA Backbone Mimic with Enhanced Polymerase Compatibility

**DOI:** 10.1021/jacs.1c08057

**Published:** 2021-09-21

**Authors:** Sven Epple, Aman Modi, Ysobel R. Baker, Ewa Wȩgrzyn, Diallo Traoré, Przemyslaw Wanat, Agnes E. S. Tyburn, Arun Shivalingam, Lapatrada Taemaitree, Afaf H. El-Sagheer, Tom Brown

**Affiliations:** † Chemistry Research Laboratory, University of Oxford, Oxford, OX1 3TA, U.K.; ‡ Chemistry Branch, Department of Science and Mathematics, Faculty of Petroleum and Mining Engineering, Suez University, Suez 43721, Egypt

## Abstract

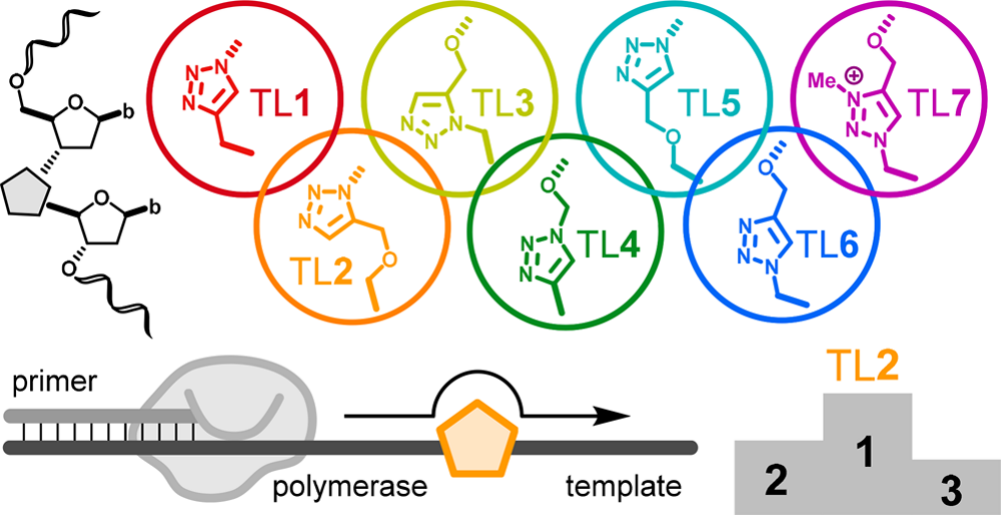

Triazole linkages (TLs) are mimics of the phosphodiester bond in oligonucleotides with applications in synthetic biology and biotechnology. Here we report the RuAAC-catalyzed synthesis of a novel 1,5-disubstituted triazole (TL**2**) dinucleoside phosphoramidite as well as its incorporation into oligonucleotides and compare its DNA polymerase replication competency with other TL analogues. We demonstrate that TL**2** has superior replication kinetics to these analogues and is accurately replicated by polymerases. Derived structure–biocompatibility relationships show that linker length and the orientation of a hydrogen bond acceptor are critical and provide further guidance for the rational design of artificial biocompatible nucleic acid backbones.

## Introduction

The replacement of natural phosphodiester (PO) bonds in DNA or RNA by artificial internucleoside linkages can generate remarkable biomimetic oligonucleotides (ONs) with applications as therapeutics,^1^ xenobiotic genetic polymers,^2−4^ aptamers,^3,5−7^ and synthetic genes.^8−10^ Biological integrity and favorable biophysical properties are critical, and good hybridization properties, mismatch discrimination, and compatibility with certain enzymes (e.g., RNase H) play crucial roles for antisense oligonucleotides.^11−15^ Other applications of backbone-modified, bioactive oligonucleotides include modified CRISPR-Cas9 systems,^16^ and compatibility with the cellular gene replication and expression machinery.

**Figure 1:**
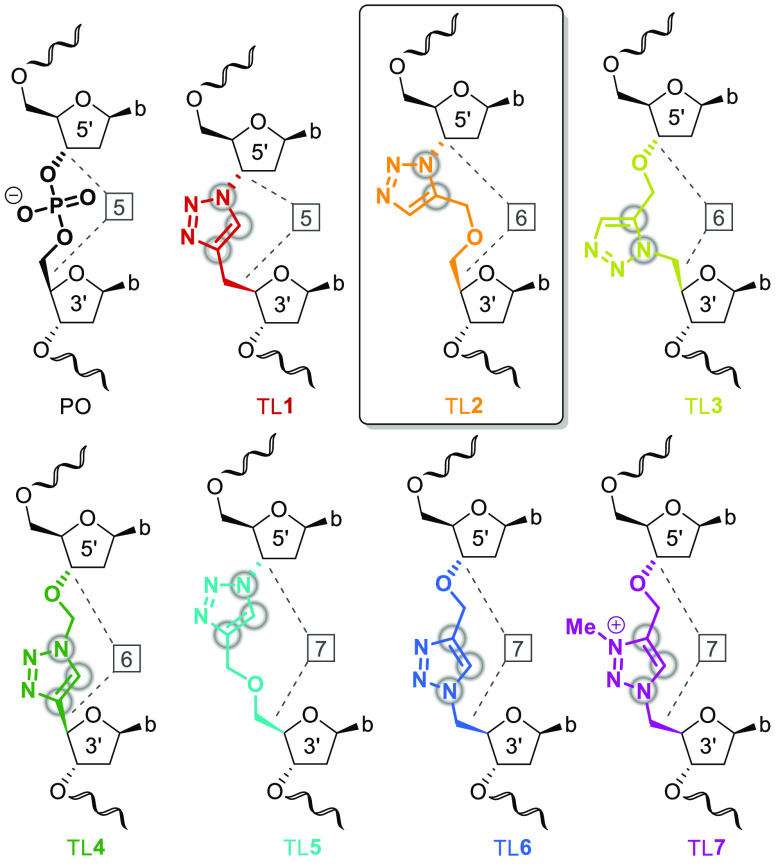
Summary of TLs connecting the 5′- and the 3′-furanose rings through multiple bonds as indicated by the boxed numbers. Trigonal planar orbital geometries (sp^2^) along the linkages are highlighted with gray circles. The structure of the natural PO is shown for reference. The TL linkage (TL2) reported here is highlighted. Abbreviations: b, base.

In general, replication-competent artificial DNA backbones can be used for gene synthesis,^8,9^ sequencing,^17^ or nucleic acid detection.^18^ A detailed study revealed several molecular characteristics of artificial backbones that are required for compatibility with DNA polymerases during replication.^19^ Such polymerase compatible artificial backbones comprise 5′-*S*-phosphorothioesters,^20^ phosphorothioates,^21^ disulfides,^22^ boranophosphates,^23^ phosphoramidates,^10,24,25^ amides,^19,26^ ureas,^18^ squaramides,^18^ and triazoles.^8,9,19,27−29^ Among these, the triazole linkage (TL) represents a powerful and versatile chemical moiety that can be readily formed by the Cu^I^-catalyzed azide–alkyne cycloaddition (CuAAC) reaction, resulting in 1,4-disubstituted 1,2,3-triazoles.^30^ Examples include TL**1**,^31^ TL**4**,^27^ TL**5**,^32^ and TL**6**^29^ (Figure 1).^33,34^ Further modification can be achieved by alkylation of the triazole as exemplified by cationic TL**7**,^19^ which is made by methylation of TL**6**. Many applications of such triazole backbones exist. For example, the CuAAC click ligation of 3′-azido ONs with 5′-alkyne adaptor ONs to form TL**1** was recently described for the preparation of next-generation sequencing libraries.^17^ However, the authors reported low replication efficiencies by several polymerases through TL**1**. Furthermore, click ligation has been used to assemble long DNA templates with isolated TL**6** modifications which can be replicated^9,29^ or transcribed^8,9^ by polymerases in bacterial^9^ or mammalian^8^ cells while retaining high fidelity read-through.^8,9,19,29^ However, TL**6** suffers from reduced binding affinity to a complementary DNA or RNA target^35−38^ and induces a TL**6**-dependent slowdown in PCR replications.^9^ In search of the ideal nucleic acid triazole linkage, we recently developed a 1,5-disubstituted 1,2,3-triazole internucleoside linkage which was prepared by Ru^II^-catalyzed azide–alkyne cycloaddition (RuAAC) (TL**3**; Figure 1).^38^ Altogether, a diverse toolbox of TLs has been reported over the past two decades, all having distinct triazole orientations and linker lengths as indicated in Figure 1. However, whether the more recent triazole isoforms, such as the 1,5-disubstituted TL**3**, retain compatibility for replication by DNA polymerases had not yet been determined.

Here we report the synthesis of a novel, 1,5-disubstituted TL connecting two nucleosides through 6 bonds (TL**2**; Figure 1). We directly compare the effect of RuAAC vs CuAAC to form TLs from the same precursors (TL**2** vs TL**5**), and we study the ability of oligonucleotides containing these TLs to form duplexes with a DNA or RNA target in comparison to reported triazoles TL**1**, TL**3**, TL**4**, and TL**6**.^38^ Moreover, we test how efficiently polymerases are able to read through TLs **1**–**7** in replication templates. For the first time, we assess replication through a 1,5-disubstituted 1,2,3-triazole (TL**2** and TL**3**). Evaluation of the molecular characteristics of the TLs with respect to conferred biocompatibility provides guidance for further biomimetic nucleic acid linkage designs.

## Results and Discussion

### Synthesis of TL-Linked Dinucleosides

**Scheme 1:**
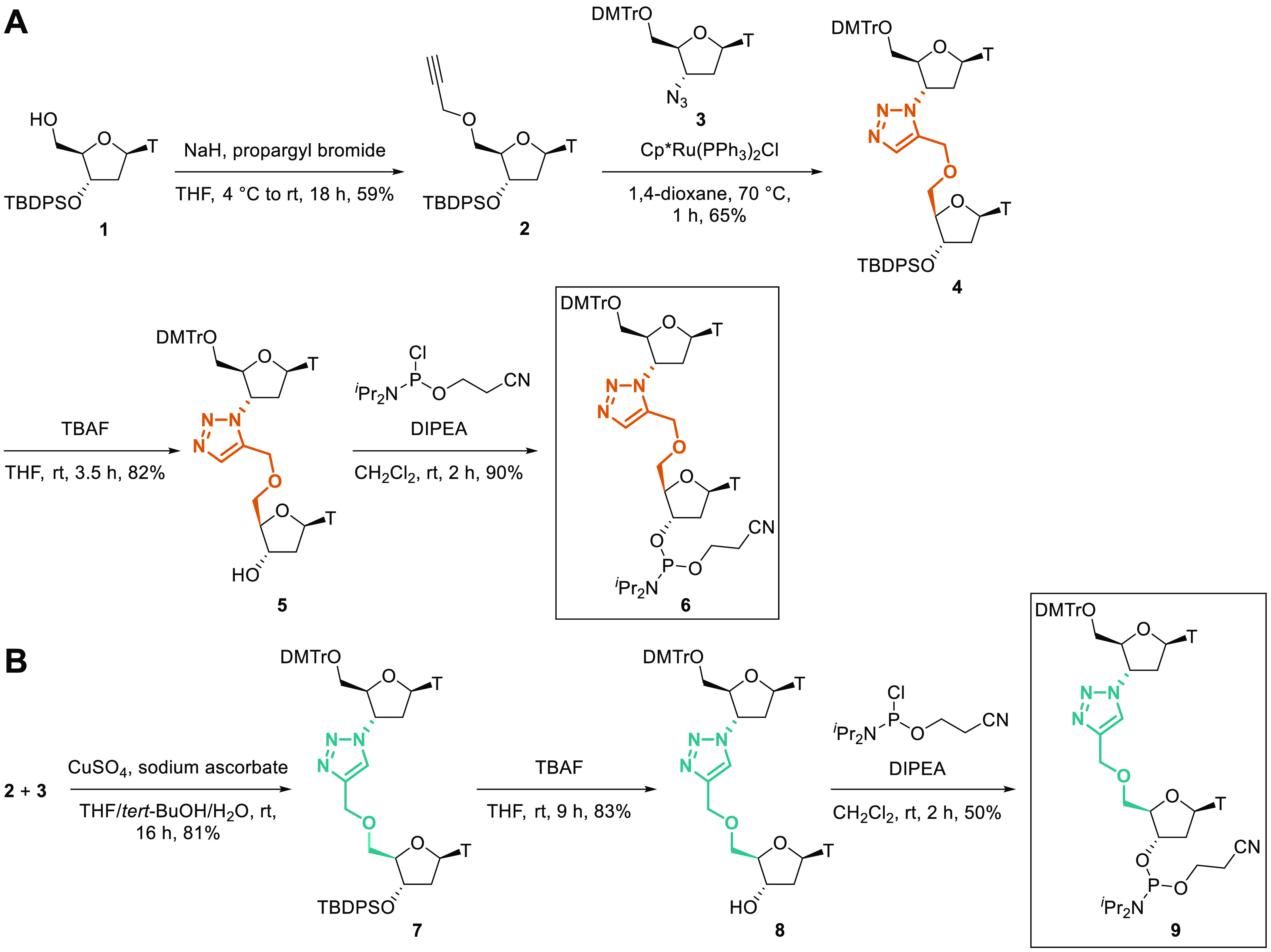
Synthesis of 1,5-TL Phosphoramidite **6** (A) and 1,4-TL Phosphoramidite **9** (B) Abbreviations: DIPEA, *N*,*N*-diisopropylethylamine; DMTr, 4,4′-dimethoxytrityl; T, thymine; TBAF, tetra-*n*-butylammonium fluoride; TBDPS, *tert*-butyldiphenylsilyl.

While the 1,5-substitution of a TL (TL**3**) dinucleotide has been recently reported,^38^ the corresponding RuAAC ligation of azide- and alkyne-modified ONs to form such triazole isoforms has not yet been developed. Thus, we opted for a dinucleoside strategy to incorporate TL**2** into ONs. A suitable dinucleoside phosphoramidite building block containing the 1,5-disubstituted triazole results from RuAAC between alkyne **2** and azide **3**(39) (Scheme 1A). A previously described method to selectively alkylate the 5′-OH of thymidine in a single step^40^ was unsuccessful and resulted in alkylation of the thymine nucleobase instead of the desired 5′-OH (Figure S1). Thus, silyl-protected thymidine **1**(41) served as a substrate for propargylation of the 5′-OH to give alkyne **2**. RuAAC with azide **3**(39) gave dimer **4** with the correct 1,5-substitution (Figure S2). TBAF-mediated deprotection of **4** gave alcohol **5**, which was phosphitylated to dimer phosphoramidite **6** for ON synthesis.

In parallel, CuAAC of alkyne **2** and azide **3** resulted in the formation of 1,4-disubstituted dimer **7** (Scheme 1B). Desilylation gave alcohol **8**, and phosphitylation using standard conditions yielded phosphoramidite **9**. The 1,4-substitution of dimer **7**—as opposed to the 1,5-substitution of dimer **4**—was confirmed by 2D NMR characterization (Figure S3).

### Hybridization Studies

#### Duplex Stability

Phosphoramidites **6** and **9** were coupled during standard ON synthesis to introduce TL**2** and TL**5** into ON2 and ON5, respectively (Table 1). The ON sequence and position of the TLs are identical to previously reported ONs with TL**1**, TL**3**, TL**4**, and TL**6** (ON1, ON3, ON4, and ON6)^38^ to facilitate accurate comparisons among the TLs in UV melting experiments (Table 1). In comparison to unmodified ON7, introduction of TL**2** results in destabilization of the duplex formed with DNA or RNA by −5.1 °C and −3.4 °C, respectively (Table 1). TL**5** in ON5 destabilizes the duplexes with DNA and RNA by −8.6 °C, confirming previously reported destabilization by the TL**5** modification in a different sequence context.^32^ Destabilizing effects have also been reported for TL**1**, TL**3**, TL**4**, and TL**6** (Table 1).^38^ All 6-bond TLs (TLs **2**–**4**) generally show more favorable duplex stability properties than their shorter 5-bond (TL**1**) or longer 7-bond (TL**5** and TL**6**) counterparts. Rigid TL**1** is the most destabilizing among the TLs. It is noteworthy that TL**4** introduces minimal duplex destabilization (only −0.8 °C) when hybridized to an RNA target. To summarize, the relative duplex stabilities of TL-modified ONs with a DNA target are TL**3** > TL**4** > TL**2** > TL**6** > TL**5** > TL**1**, and with an RNA target, TL**4** > TL**3** ≥ TL**2** > TL**6** > TL**5** > TL**1**.

**Table 1:** Melting Temperatures (*T*_m_)^a^ of TL-Modified ONs with a DNA^b^ and RNA^c^ Target

ON	Sequence (5′ → 3′)^d^	DNA^b^ target *T*_m_ (Δ*T*_m_)/°C	RNA^c^ target *T*_m_ (Δ*T*_m_)/°C
ON1	CGACGT^**TL1**^TTGCAGC	(−11.2)^38^	(−13.6)^38^
ON2	CGACGT^**TL2**^TTGCAGC	57.0 (−5.1)	55.5 (−3.4)
ON3	CGACGT^**TL3**^TTGCAGC	(−2.9)^38^	(−3.3)^38^
ON4	CGACGT^**TL4**^TTGCAGC	(−3.2)^38^	(−0.8)^38^
ON5	CGACGT^**TL5**^TTGCAGC	53.5 (−8.6)	50.3 (−8.6)
ON6	CGACGT^**TL6**^TTGCAGC	(−7.4)^38^	(−5.3)^38^
ON7	CGACGTTTGCAGC	62.1	58.9

a Values were obtained from the maxima d*A*_260_/d*T* vs *T* for 3 μM of each ON in 10 mM phosphate buffer, 200 mM NaCl, pH 7.0. The graphs of d*A*_260_/d*T* vs *T* are shown in Figure S4. Δ*T*_m_ are relative to the unmodified control ON7.

b Sequence of DNA target = 5′-GCTGCAAACGTCG-3′.

c Sequence of RNA target = 5′-GCUGCAAACGUCG-3′.

d ^TL^ indicates the site where a PO is replaced by TLs **1**–**6**.

#### Duplex Structure

**Figure 2:**
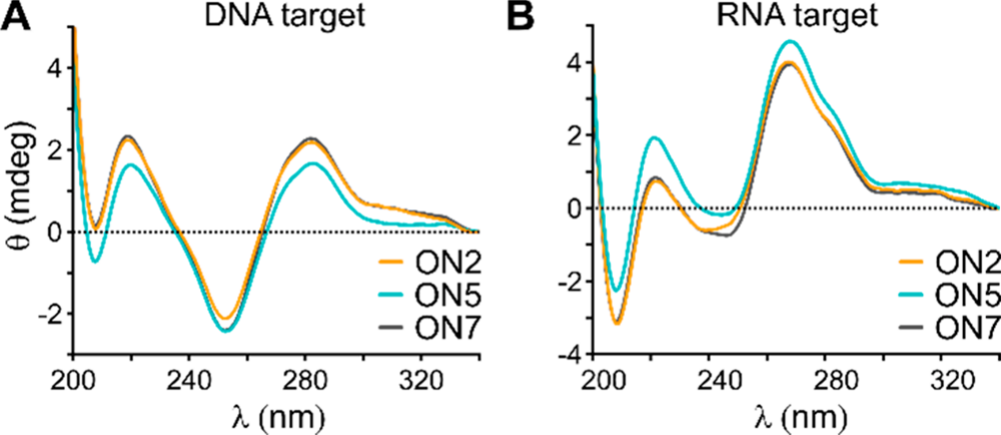
CD spectra of ON2, ON5 and ON7 in duplex with (A) DNA and (B) RNA containing 3 μM of ON and 3 μM of the target in 10 mM phosphate buffer, 200 mM NaCl, pH 7.0. Data points were taken as an average of four scans at rt. DNA target = 5′-GCTGCAAACGTCG-3′, RNA target = 5′-GCUGCAAACGUCG-3′.

Despite some destabilizing effects, TL**1**, TL**3**, TL**4**, and TL**6** are known to retain global B- or A-form duplex structures with the DNA or RNA target, respectively.^38^ In the current study, duplexes formed by TL**2**- and TL**5**-modified ON2 and ON5 with complementary DNA or RNA were analyzed by circular dichroism (CD) spectroscopy (Figure 2). The duplexes of ON2 with DNA and RNA show almost identical spectra to the equivalent duplexes formed by unmodified ON7. Duplexes of ON5 with DNA or RNA also retain global B- or A-form helical structures, respectively.

### Enzymatic Read-through Studies

**Figure 3:**
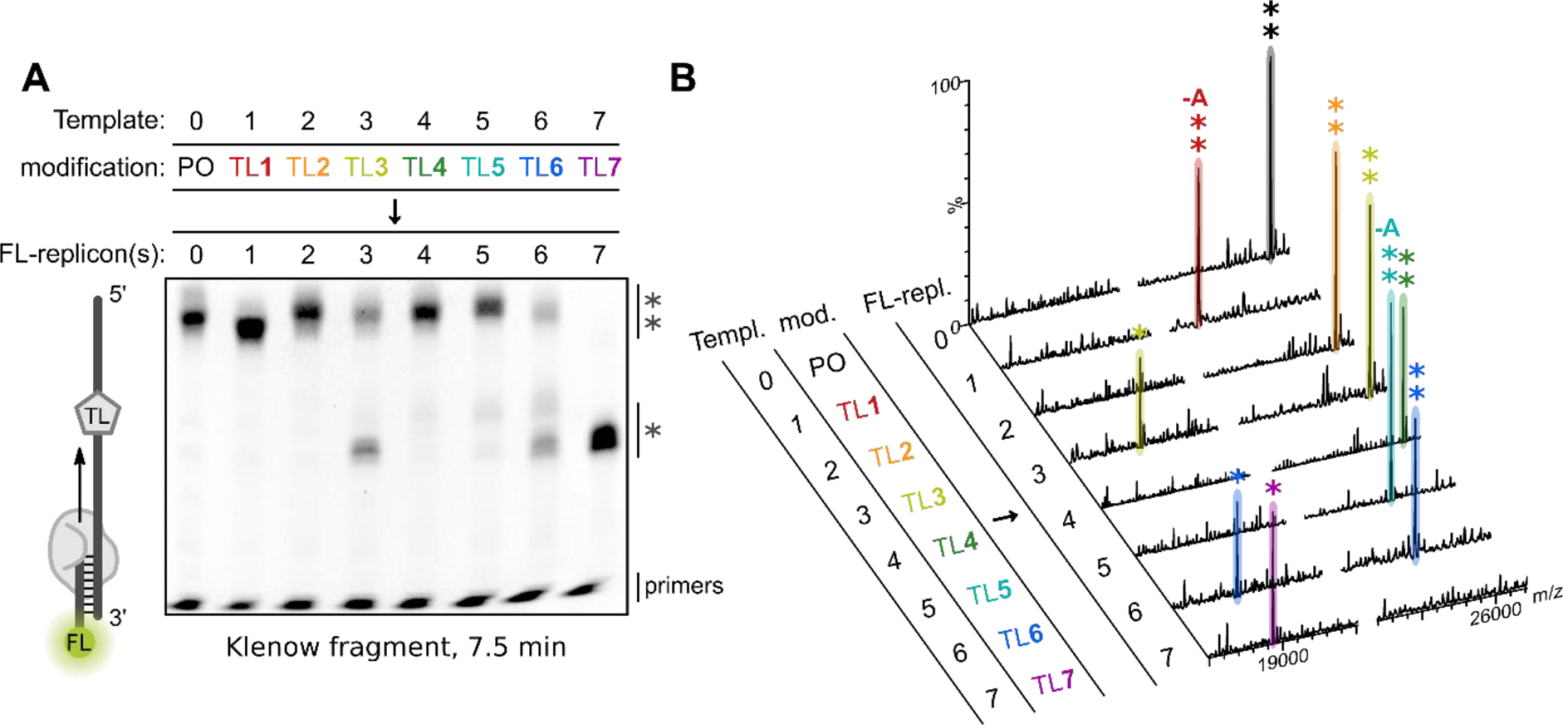
Klenow fragment-mediated extension of FL-tagged primers. (A) Polyacrylamide gel electrophoresis (PAGE) of FL-labeled extension products after incubation with Klenow fragment at 37 °C for 7.5 min using either unmodified Template 0 (PO) or templates containing a single TL modification: Template 1 (TL1), Template 2 (TL2), Template 3 (TL3), Template 4 (TL4), Template 5 (TL5), Template 6 (TL6), or Template 7 (TL7). The FL-label was visualized (λ_ex_ = 460 nm, λ_em_ = 516–600 nm). Read-through and truncated replicons are marked with (**) and (*), respectively. (B) MS analysis of the primer extension experiments using Klenow fragment at 37 °C for 7.5 min. Templates (Templ.) and their modifications (mod.) are identical to (A). Identified peaks for read-through and truncated FL-labeled replicons (FL-repl.) are marked with (**) and (*), respectively. –A denotes an identified A-deletion. For sequences of templates and FL-primers see Table S1. For full mass spectra and peak assignments see Figures S5–S7 and Table S2.

**Figure 4:**
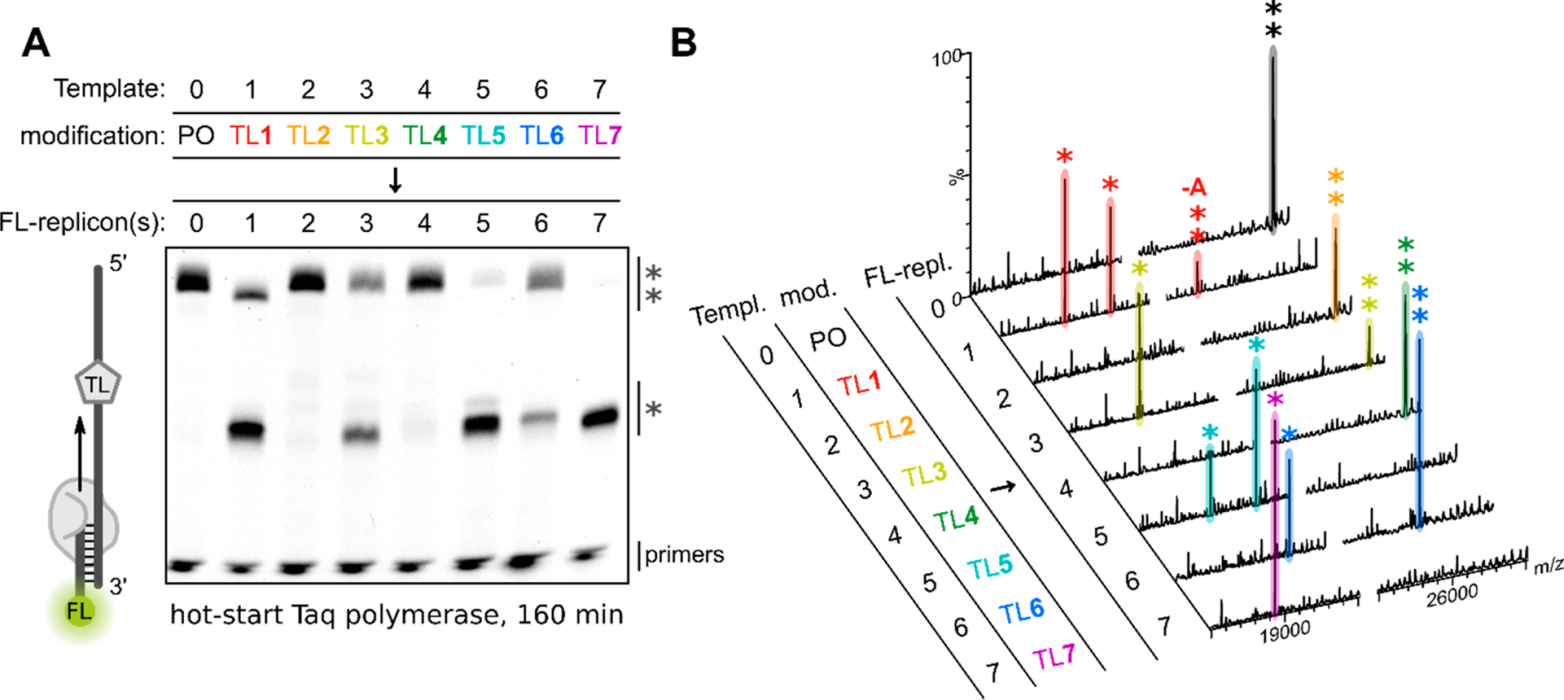
Hot-start Taq-mediated extension of FL-tagged primers. (A) PAGE of FL-labeled extension products after incubation with hot-start Taq at 60 °Cfor 160 min using either unmodified Template 0 (PO) or templates containing a single TL modification: Template 1 (TL1), Template 2 (TL2), Template 3 (TL3), Template 4 (TL4), Template 5 (TL5), Template 6 (TL6), or Template 7 (TL7). The FL-label was visualized (λ_ex_ = 460 nm, λ_em_ = 516–600 nm). (B) MS analysis of the primer extension experiments using hot-start Taq polymerase at 60 °C for 160 min. Templates (Templ.) and their modifications (mod.) are identical to (A). Identified peaks for read-through and truncated FL-labeled replicons (FL-repl.) are marked with (**) and (*), respectively. −A denotes an identified A-deletion. For sequences of templates and FL-primers see Table S1. For full mass spectra and peak assignments see Figures S8–S10 and Table S3.

The ability of TL-modified ONs (TLs **1**–**6**) to form duplexes with DNA and RNA targets while maintaining global structural integrity suggests that these TLs could be promising biocompatible DNA backbone mimics. Indeed, TL**1**, TL**6**, and methylated TL**7** have been extensively studied as modifications in templates for replication by DNA and RNA polymerases.^8,9,16,19,29,42^ Moreover, a previous study reported encouraging results for TL**4** within modified primers in qPCR.^27^ However, no read-through compatibilities of 1,4-disubstituted TL**5** or 1,5-disubstituted TLs (TL**2** and TL**3**) have been reported. Thus, we compared TLs **1**–**7** in primer extension studies (Figure 3 and Figure 4). We synthesized unmodified templates and TL-modified templates, each containing a single TL (TLs **1**–**7**), using phosphoramidite **6** (TL**2**), **9** (TL**5**), or previously reported phosphoramidites (TL**1**,^19^ TL**3**,^38^ TL**4**,^27^ TL**6**,^43^ and TL**7**^19^). We then assessed the polymerase compatibilities of TLs **1**–**7** by enzymatic extension of fluorescein (FL)-labeled primers followed by analysis of the FL-labeled products after gel electrophoresis. The sequences of the templates and primers can be found in Table S1.

#### Klenow Fragment

Extension of an FL-labeled primer by the large fragment of DNA polymerase I (Klenow fragment) using unmodified Template 0 (PO) resulted in formation of the expected full-length product, which was confirmed by mass spectrometry (MS) analysis (FL-replicon 0**; Figure 3A,B). Primer extension through the TL**1**-modified Template 1 gave a band that migrated slightly faster than FL-replicon 0 and a mass corresponding to a replicon with an A-deletion (FL-replicon 1**; Figure 3A,B). The fact that read-through of TL**1** by Klenow fragment results in an A-deletion mutation which is situated next to the TL**1** was previously shown by sequencing of the replicons.^19^ Templates bearing 6-bond TLs **2**–**4** resulted in the formation of the expected full-length products as confirmed by MS (FL-replicons 2**, 3**, and 4**; Figure 3A,B). However, replication of the TL**3** template also produced a truncated product, shown by MS analysis to be a consequence of premature extension termination directly before the triazole (FL-replicon 3*; Figure 3A,B). The longer 7-bond TL**5** in Template 5 was replicated but resulted in a product with an A-deletion (FL-replicon 5**; Figure 3A,B), and additional faint truncation bands were visible on the gel. Extension through TL**6** produced the expected full-length product (FL-replicon 6**; Figure 3A,B) and truncated products, one of which results from termination directly before TL**6** (FL-replicon 6*; Figure 3A,B). The presence of cationic methylated TL**7** in Template 7 is catastrophic to the primer extension and exclusively results in a truncated product from extension termination directly before the methylated triazole (FL-replicon 7*; Figure 3A,B). This confirms previous reports for this TL.^19^

#### Hot-Start Taq Polymerase

It is known that replication by Taq polymerase is slowed down at high DNA concentrations such as those used in the primer extension assays.^44^ Moreover, a previous study showed that the replication of an unmodified template can be significantly slowed down under these conditions (up to 120 min).^19^ Thus, primer extensions along the TL-modified templates were performed with hot-start Taq polymerase at 60 °C for an extended time of 160 min (Figure 4). Replication of unmodified Template 0 (PO) resulted in the exclusive formation of the expected full-length product containing a Taq-characteristic 3′-A overhang as determined by MS (FL-replicon 0**; Figure 4A,B). Replication of the TL**1**-modified template resulted in minor amounts of a product for which MS suggested deletion of an A-nucleotide (FL-replicon 1**; Figure 4A,B). The major products from TL**1** were truncated ONs where replication stopped directly before or after the triazole (FL-replicons 1*; Figure 4A,B). This supports the mutagenic effect of TL**1** in causing deletions around the artificial linkage with a Taq polymerase.^19^ Remarkably, replication through TL**2** yielded a clean full-length product including the expected Taq-characteristic 3′-A overhang (FL-replicon 2**; Figure 4A,B). No truncated products were detected on the gel or by MS. In contrast, a significant amount of truncated product was observed for the TL**3** template where extension terminated directly before the triazole (FL-replicon 3*; Figure 4A,B). Extension through TL**4** resulted in the expected full-length product without truncation (FL-replicon 4**; Figure 4A,B). In contrast to the previous primer extension experiment with Klenow fragment, replication through TL**5** using hot-start Taq polymerase mainly resulted in truncated products from termination directly before or after the triazole (FL-replicons 5*; Figure 4A,B). Replication of the TL**6** template produced the correct full-length construct (FL-replicon 6**; Figure 4A,B) and a truncated product (FL-replicon 6*; Figure 4A,B). As seen for Klenow fragment, methylated TL**7** completely blocks extension (FL-replicon 7*; Figure 4A,B). Across the Klenow and hot-start Taq primer extension assays, TL**2** and TL**4** stand out as being the only TLs resulting in the clean formation of the expected full-length product without any detectable mutations or truncated fragments observed on gel or by MS. The excellent read-through compatibility of TL**2** by Klenow fragment and hot-start Taq polymerase was confirmed with an alternative sequence (Template 9; Figures S11, S12 and Table S4).

#### qPCR Kinetics

**Figure 5:**
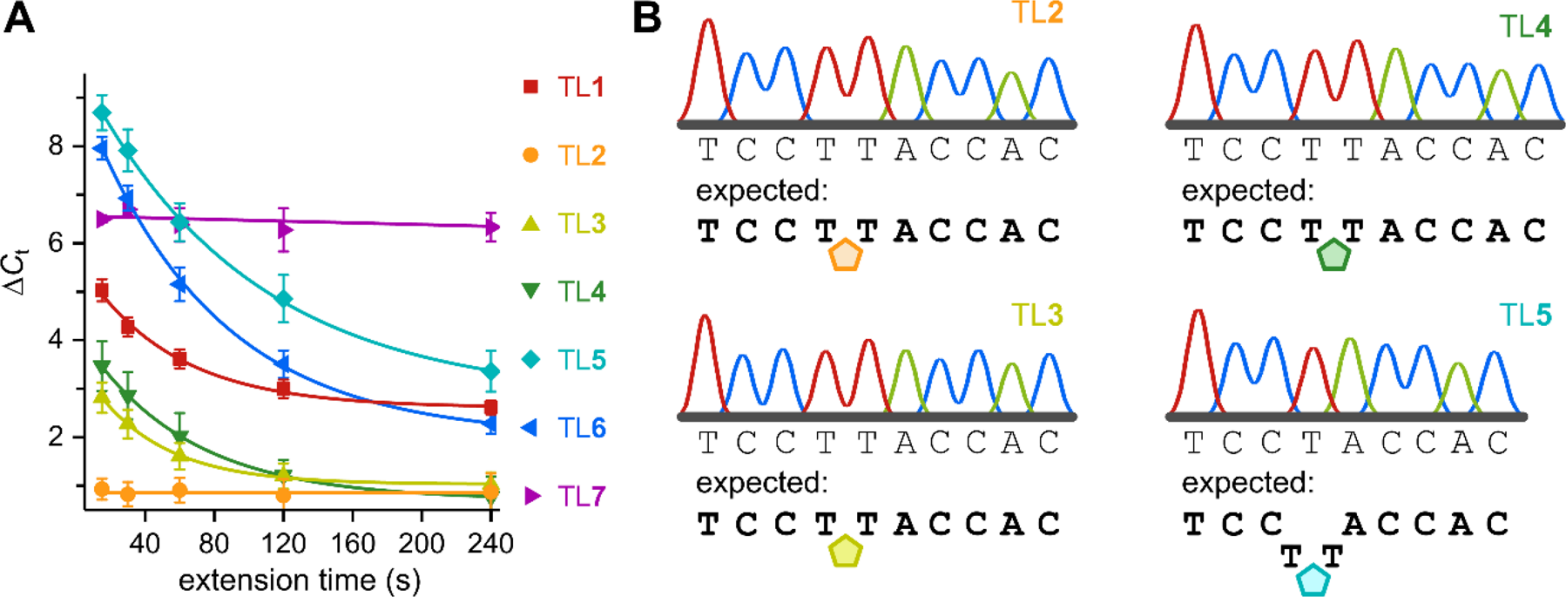
qPCR kinetics and PCR product sequencing. (A) Differences of threshold cycles (Δ*C*_t_s) of TL-modified templates to their respective isosequential unmodified control template from qPCRs with hot-start Taq polymerase as a function of extension time. Unmodified templates: Template 0 and Templates 10–12. TL-modified templates: Template 1 (TL1), Template 2 (TL2), Template 3(TL3), Template 4 (TL4), Template 5 (TL5), Template 6 (TL6), and Template 7 (TL7). Δ*C*_t_ = *C*_t_(TL-modified) – *C*_t_(unmodified) for each extension time. Data are presented as the average from triplicates ± standard error of the mean. For *C*_t_’s see Figure S22. (B) Representative Sanger sequencing results for hot-start Taq PCR amplicons cloned into a vector. Amplicons result from PCR amplifications of Template 2 (TL2), Template 3 (TL3), Template 4 (TL4), and Template 5 (TL5) using hot-start Taq polymerase with an extension time of 30 s each cycle. Corresponding positions of the TLs in the templates are indicated as pentagons. For full template sequences see Table S1, Figure S23, and Figure S24.

To assess the time dependencies of TLs **1**–**7**, the modified templates were used in qPCR experiments with various extension times. Impaired read-through of a given template results in an increase of the threshold cycle (*C*_t_)—the number of qPCR cycles after which a signal overcomes a set threshold. If impairment can be overcome with time, the resulting *C*_t_ value will decrease with longer extension times if within the range of the time dependency. Amplification of unmodified templates can be considered as being time independent under the conditions of the study, as all internucleoside linkages are natural POs which are formed rapidly. *C*_t_ values of qPCR reactions with extension times ranging from 15–240 s were determined for templates with TLs **1**–**7** using hot-start Taq polymerase (Figures S13–S18). The differences between the determined *C*_t_ values of the modified templates to the *C*_t_ values of the respective isosequential unmodified PO templates (Δ*C*_t_) are plotted for each extension time in Figure 5A. Except for TL**2** and TL**7**, all TLs show time dependency for the read-through of the artificial linkage. The apparent time independency of TL**7** has been previously explained by a thermodynamic barrier created by TL**7** that can only be overcome when transitioning from extension (60 °C) to denaturation (95 °C) in each cycle.^19^ Most notably, the shorter 5- and 6-bond TLs **1**–**4** are less time dependent than the longer 7-bond TL**5** and TL**6**. Indeed, Δ*C*_t_’s of TL**5** and TL**6** continued to decrease up to 240 s, the highest extension time tested in this study. The Δ*C*_t_’s of TL**1**, TL**3**, and TL**4** decrease with longer extension times, but the fitted curves flatten toward an extension time of 240 s. In contrast, the Δ*C*_t_ of TL**2** showed no time dependency within the tested conditions. Together with the superior results from the primer extension experiments, this suggests that the observed time independency of TL**2** does not originate from transitions between the cycles as seen for TL**7**. Remarkably, there is no significant kinetic barrier imposed by TL**2** for read-through of hot-start Taq polymerase under the tested conditions.

#### DNA Sequencing

To verify that read-through of the TL-modified templates results in correct replication, PCR products from hot-start Taq polymerase-mediated amplification were sequenced. While TL**1**, TL**6**, and TL**7** have been previously studied for replication fidelity,^19^ the question whether TLs **2**–**5** can be replicated correctly in PCR remains unanswered. Thus, PCR amplicons generated using hot-start Taq polymerase and templates containing TL**2**, TL**3**, TL**4**, or TL**5** were cloned into a vector and transformed into *Escherichia coli*. Several colonies were randomly picked, and the recovered vectors were analyzed by Sanger sequencing (Figure 5B). The 6-bond triazole backbones (TLs **2**–**4**) resulted in the correct read-through producing the expected sequence for all colonies picked (*n* = 4). In contrast, the longer 7-bond TL**5** resulted in a deletion mutation around the artificial linkage for which the T^TL**5**^T motif has been read as a single T in four out of five colonies picked.

### Structure–Biocompatibility Relationship

Analysis of the molecular properties of TLs **1**–**7** provides insight into the structure–biocompatibility relationship among the TLs for replication (Table 2). Most noticeable are the differences in TL backbone lengths. For 5-bond TL**1**, whose number of bonds is closest to the natural PO, inefficient read-through by hot-start Taq was observed confirming previous reports.^17,19^ Moreover, TL**1** is mutagenic and results in single point deletions around the triazole when read by Klenow fragment or Taq polymerases.^19^ The read-through efficiencies of 6-bond TLs **2**–**4** gave different results in the primer extension assay. However, all 6-bond TLs imposed the lowest kinetic barriers on polymerase read-through and all amplicons from PCRs with hot-start Taq polymerase had the correct sequences. The longer 7-bond TL**5** expressed poor read-through efficiency in the primer extension assay with hot-start Taq polymerase and resulted in deletion mutations in PCR amplicons. In contrast, 7-bond TL**6** is known to be read-through correctly resulting in the expected amplicon sequences.^19^ Previous studies suggested that flexibility of the artificial backbone is required for read-through.^19^ There is an overall increase of flexibility along the TL backbone of 1,5-disubstituted TLs (TL**2** and TL**3**: 2 × sp^2^; Table 2 and Figure 1) compared to 1,4-disubstituted TLs (TL**1** and TLs **4**–**6**: 3 × sp^2^; Table 2 and Figure 1). Moreover, the poor biocompatibility of 5-bond TL**1** might be attributed to the combination of increased rigidity and highest duplex destabilization, defeating the positive contributions from being closest to the natural PO linker length. However, the flexibility model does not explain the lower replication efficiency of 6-bond TL**3** (2 × sp^2^; Table 2 and Figure 1) compared to 6-bond TL**4** (3 × sp^2^; Table 2 and Figure 1) and 6-bond TL**2** (2 × sp^2^; Table 2 and Figure 1) in the primer extension studies (TL**2**, TL**3** and TL**4**; Figure 3A and Figure 4A). Taq polymerase mainly interacts with the DNA through hydrogen bonding with the phosphates,^45^ and a crucial factor in read-through of artificial backbones is the ability to donate an electron pair as a hydrogen bond acceptor.^19^ As such, undesirable orientation of the hydrogen bond acceptor in TL**3** is a plausible reason for its hampered read-through compatibility. Moreover, *N*3-methylation in TL**7** is catastrophic, further suggesting that a hydrogen acceptor is important for replication. However, it is likely that the additional charge inversion and steric hindrance contribute to the replication incompatibility of TL**7**, too.

**Table 2:** Summary of the Structural Characteristics and Read-through Compatibilities of TLs **1**–**7**

TL	linker length^a^	No. sp^2^ centers^b^	kinetic barrier (Taq)	Correct read-through (Taq)^c^
PO	5	0	–	–
TL**1**	5	3	medium/high	no, deletion^19^
TL**2**	6	2	not significant	yes
TL**3**	6	2	medium	yes
TL**4**	6	3	medium	yes
TL**5**	7	3	High	no, deletion
TL**6**	7	3	High	yes^19^
TL**7**	7	3	very high	no, deletions^19^

a Defined as the minimal number of bonds connecting the 5′- and the 3′-furanose rings.

b Along the internucleoside linkage.

c Defined as having the correct sequence after read-through has occurred.

Taking these observations together, we hypothesize that the ideal TL results from a combination of linker length, availability of a hydrogen bond acceptor, and overall flexibility. Extending this to any artificial DNA backbone, we propose that the artificial backbone should be 5–6 bonds in length and provide an accessible hydrogen bond acceptor, while maintaining an overall high degree of flexibility. The new 1,5-disubstituted TL**2** combines these molecular requirements and possesses outstanding biocompatibility. The proposed molecular requirements also agree with our previously described model, which was derived from read-through compatibility tests of a range of backbone linkages including amides, phosphoro(di)thioates, phosphoramidates, and squaramides.^18,19,24,25^ It is important to emphasize that the observed read-through compatibilities are dependent on the polymerase and similar polymerase dependencies were reported for other PO mimics.^18,19^

## Conclusions

In conclusion, triazole linkages are interesting phosphodiester mimics for applications in synthetic biology, biotechnology, and other areas. A profound understanding of the structure–biocompatibility relationship among different TLs is crucial for their successful application in biochemical and biological systems. Here we report the synthesis of novel 1,5-disubstituted TL**2** by RuAAC and directly compare this to its 1,4-disubstituted equivalent TL**5**, which is formed by CuAAC. In duplex melting experiments, TL**2**-modified ON2 forms more stable duplexes with DNA and RNA targets than TL**5**-modified ON5. Moreover, the read-through kinetics and amplification of TL**2** by Klenow fragment and Taq DNA polymerase exceed all other TLs tested. To the best of our knowledge, TL**2** has the fastest read-through kinetics and the highest efficiency of full-length product formation by Taq polymerase among the TLs reported to date. Further studies will be necessary to determine if the excellent read-through compatibility of TL**2** by Taq can be translated into a cellular environment and be accommodated by RNA polymerases as previously demonstrated for TL**6**.^8,9,29^ Moreover, direct comparison between TL**2** and TL**5** revealed a remarkable difference in read-through accuracy. While TL**2** was read correctly by hot-start Taq polymerase, TL**5** resulted in a deletion mutation around the triazole linkage. We have discussed the structure–biocompatibility relationship of several TLs, and we provide a rationale to assist future designs of artificial backbone mimics in replication templates. Importantly, the structure–biocompatibility relationship presented in this study is derived from ONs with TLs between two thymidines, providing a rather challenging sequence context for polymerase read-through. Efficiencies can vary with the nucleobase sequence and generally improve with greater duplex stability (a higher GC-content) around the triazole. Indeed, enhanced read-through compatibility was previously observed for a C^TL**6**^C motif.^19,29^ Hence, we anticipate that further investigations using different sequences will provide additional validation of the proposed model. The application of RuAAC to form TL**2** exemplifies a powerful strategy to increase TL flexibility through formation of 1,5-disubstituted 1,2,3-triazoles. In contrast to the widely adopted CuAAC reaction, the feasibility of the RuAAC reaction to efficiently ligate azide- and alkyne-modified ONs in high yield, and without inducing DNA damage, remains elusive. However, in light of recent advances to obtain 1,5-disubstituted 1,2,3-triazoles via water and air compatible Ni-catalyzed click chemistry,^46^ TL**2** represents a promising backbone for use in a plethora of applications across the life sciences.

## Supporting Information Available

Experimental section, characterization data and NMRs of new compounds, additional primer extension experiments, *T*_m_ curves, oligonucleotide HPLC traces, oligonucleotide mass spectra, qPCR and sequencing data (PDF)

## Abbreviations

Cas9: CRISPR associated protein 9
CRISPR: clustered regularly interspaced short palindromic repeats
DIPEA: *N*,*N*-diisopropylamine
DMTr: 4,4-dimethoxy trityl
TL: triazole linkage
qPCR: quantitative polymerase chain reaction
