# Supplementary material for: A New 1,5-Disubstituted Triazole DNA Backbone Mimic with Enhanced Polymerase Compatibility
Source: J Am Chem Soc. 2021 Sep 21;143(39):16293–301. doi: 10.1021/jacs.1c08057 (PMC8499026; doi:10.1021/jacs.1c08057)
Supplement: Supplementary file 1 — ja1c08057_si_001.pdf [file ja1c08057_si_001.pdf]

A New 1,5-Disubstituted Triazole DNA Backbone Mimic with Enhanced Polymerase Compatibility

Sven Epple[a], Aman Modi[a], Ysobel R. Baker[a], Ewa Węgrzyn[a§], Diallo Traoré[a], Przemyslaw Wanat[a#], Agnes E. S. Tyburn[a], Arun Shivalingam[a], Lapatrada Taemaitree[a], Afaf H. El-Sagheer[a,b] and Tom Brown*[a]

[a] *Department of Chemistry, University of Oxford, 12 Mansfield Road, Oxford, OX1 3TA, UK.*

[b] *Department of Science and Mathematics, Faculty of Petroleum and Mining Engineering, Suez University, Suez 43721, Egypt.*

Present Addresses:

§ *Department of Chemistry, Ludwig Maximilians University, 81377 Munich, Germany.*

Faculty of Physics, University of Warsaw, L. Pasteura 5, 02-093, Warsaw, Poland.

* Email: tom.brown@chem.ox.ac.uk

NMR spectra for structure determination 3

Additional T_m data 6

List of oligonucleotides 7

Mass spectrometry analysis of primer extension products 10

Additional primer extension experiments 18

qPCR Experiments 21

DNA sequencing 31

Protocols and procedures 33

UV melting studies 33

Circular dichroism studies 33

Linear copying assay 33

Linear copying for mass spectrometry 33

qPCR kinetics 34

DNA sequencing 34

Mass spectrometry 35

Synthesis and purification of oligonucleotides 35
Synthesis of DNA oligonucleotides 35
Synthesis of RNA oligonucleotides 35
Removal of 2′-TBS protection of RNA oligonucleotides 35
HPLC purification of oligonucleotides (DNA or RNA) 35
Oligonucleotide purification by gel electrophoresis 36
Synthetic Procedures 36
General 36
5′-*O*-Propargyl-3′-*O*-(*tert*-butyldiphenylsilyl)-thymidine (**2**) 37
5′-*O*-(4,4′-Dimethoxytrityl)-thymidine-1,5-triazole-3′-*O*-(*tert*-butyldiphenylsilyl)-thymidine dimer (**4**) 38
5′-*O*-(4,4′-Dimethoxytrityl)-thymidine-1,5-triazole-thymidine dimer (**5**) 40
5′-*O*-(4,4′-Dimethoxytrityl)-thymidine-1,5-triazole-thymidine dimer phosphoramidite (**6**) 41
5′-*O*-(4,4′-Dimethoxytrityl)-thymidine-1,4-triazole-3′-*O*-(*tert*-butyldiphenylsilyl)-thymidine dimer (**7**) 42
5′-*O*-(4,4′-Dimethoxytrityl)-thymidine-1,4-triazole-thymidine dimer (**8**) 44
5′-*O*-(4,4′-Dimethoxytrityl)-thymidine-1,4-triazole-thymidine dimer phosphoramidite (**9**) 45
UPLC-MS analysis and NMR spectra 46
References 88

NMR spectra for structure determination

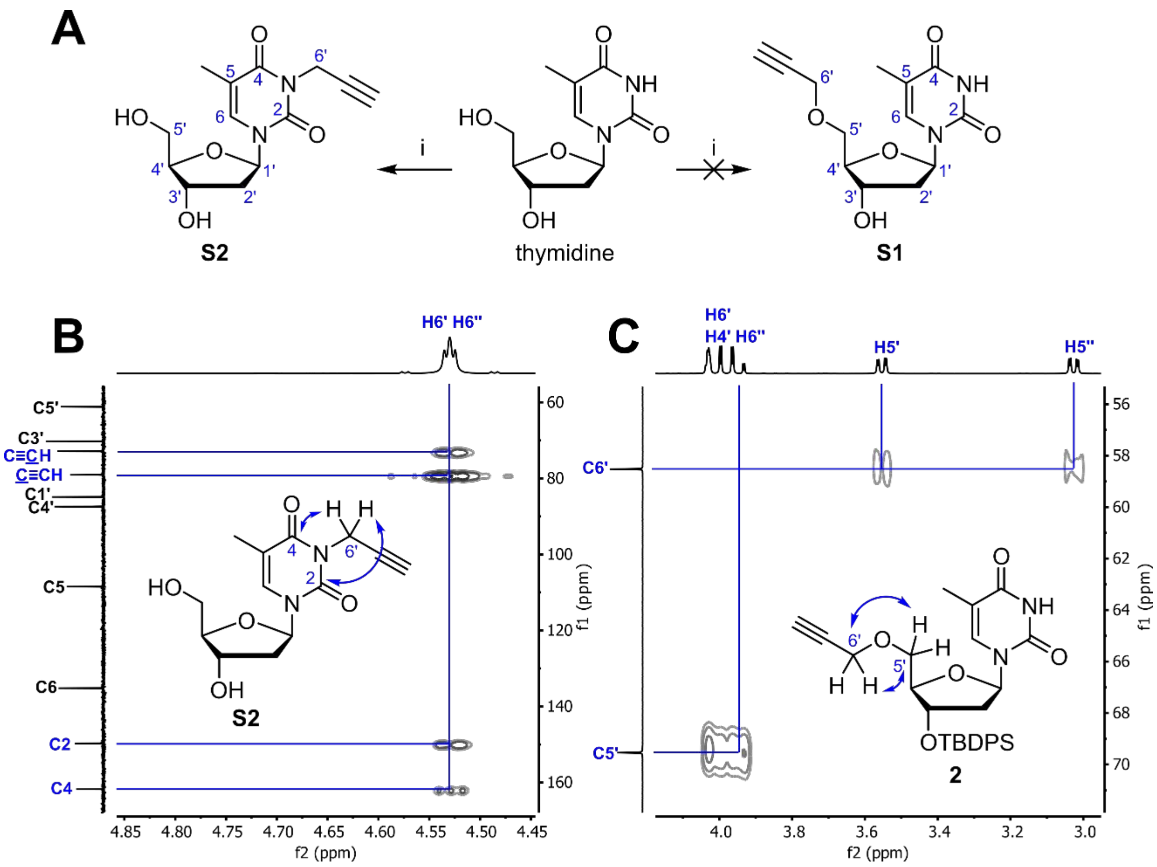

Figure S1. Determination of the propargylation site by heteronuclear multiple bond correlation (HMBC) spectroscopy. (A) Propargylation of unprotected thymidine in a single step. Reagents and conditions: i) 1,8-Diazabicyclo[5.4.0]undec-7-ene (DBU), propargyl bromide, *N*,*N*-dimethyl formamide (DMF), 60 °C, 16 h, 86% (**S2**).[1] (B) HMBC spectrum of *N*3-propargylated thymidine **S2**. (C) HMBC spectrum of 5′-*O*-propargyl-3′-*O*-(*tert*-butyldiphenylsilyl)-thymidine (**2**). ^{1}H - ^{13}C correlations are indicated with blue lines. For full spectra see **Figure S66** and **Figure S39**.

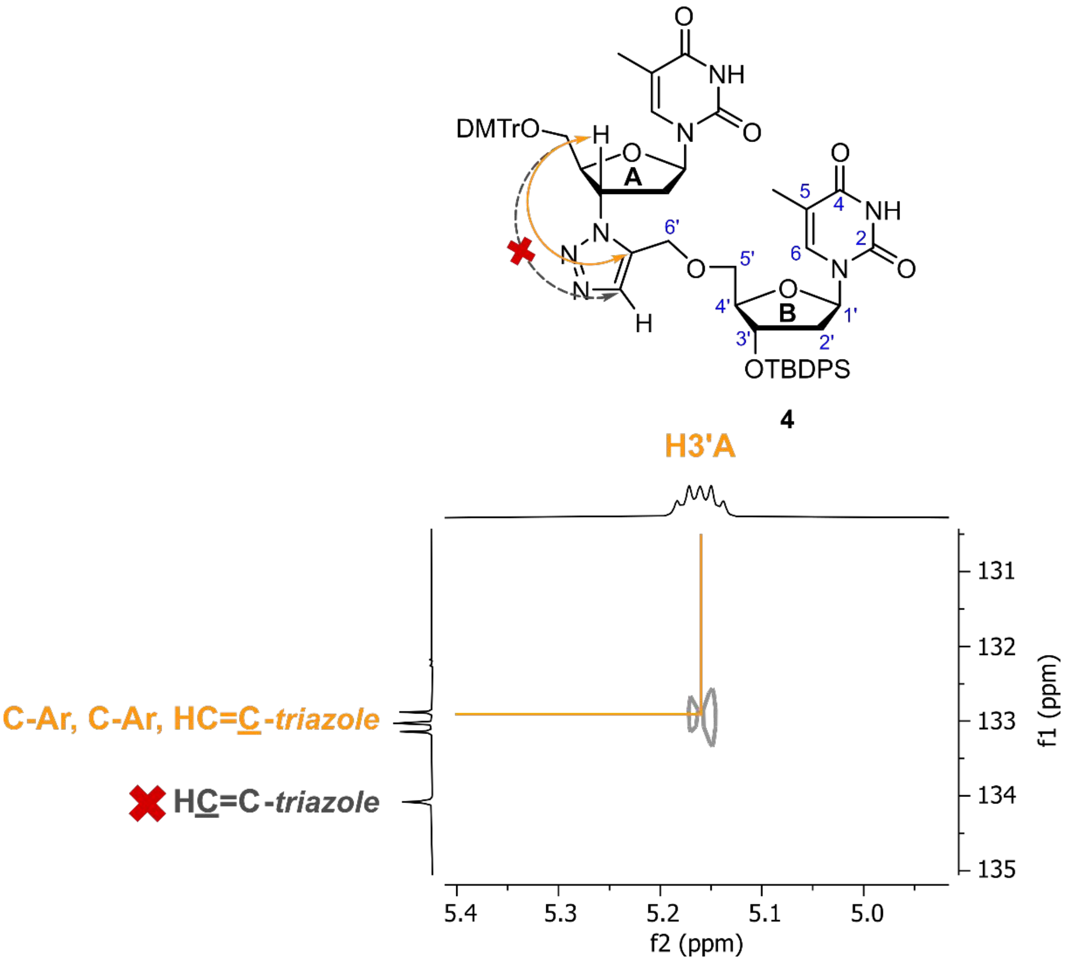

Figure S2. Determination of the 1,5-disubstitution of dimer **4** by HMBC spectroscopy. ^{1}H - ^{13}C correlation between H3′A and HC=<u>C</u>-*triazole* is indicated with orange lines. No ^{1}H - ^{13}C correlation between H3′A and H<u>C</u>=C-*triazole* was observed. For full spectrum see **Figure S44**. Note that ^{1}H - ^{13}C correlation between H3′A and H<u>C</u>=C-*triazole* but not between H3′A and HC=<u>C</u>-*triazole* was previously reported for the 3′-*O*-*tert*-butyldimethylsilyl (TBS)-protected analogue of the 1,4-disubstituted 1,2,3-triazole T-T dimer.[2]

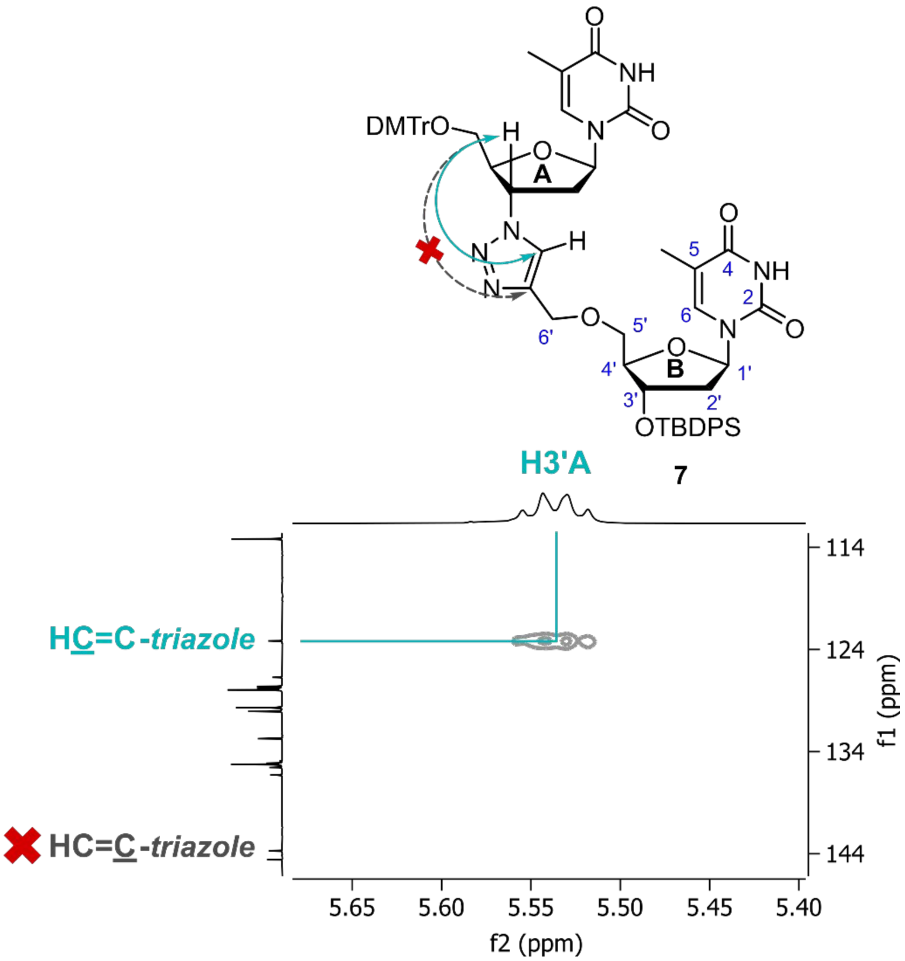

Figure S3. Determination of the 1,4-disubstitution of dimer **7** by HMBC spectroscopy. ^{1}H - ^{13}C correlation between H3′A and HC=C-*triazole* is indicated with blue lines. No ^{1}H - ^{13}C correlation between H3′A and HC=C-*triazole* was observed. For full spectrum see **Figure S55**. Note that a similar ^{1}H - ^{13}C correlation between H3′A and HC=C-*triazole* but not between H3′A and HC=C-*triazole* was previously reported for the 3′-*O*-TBS-protected analogue of the 1,4-disubstituted 1,2,3-triazole T-T dimer.[2]

Additional T_m data

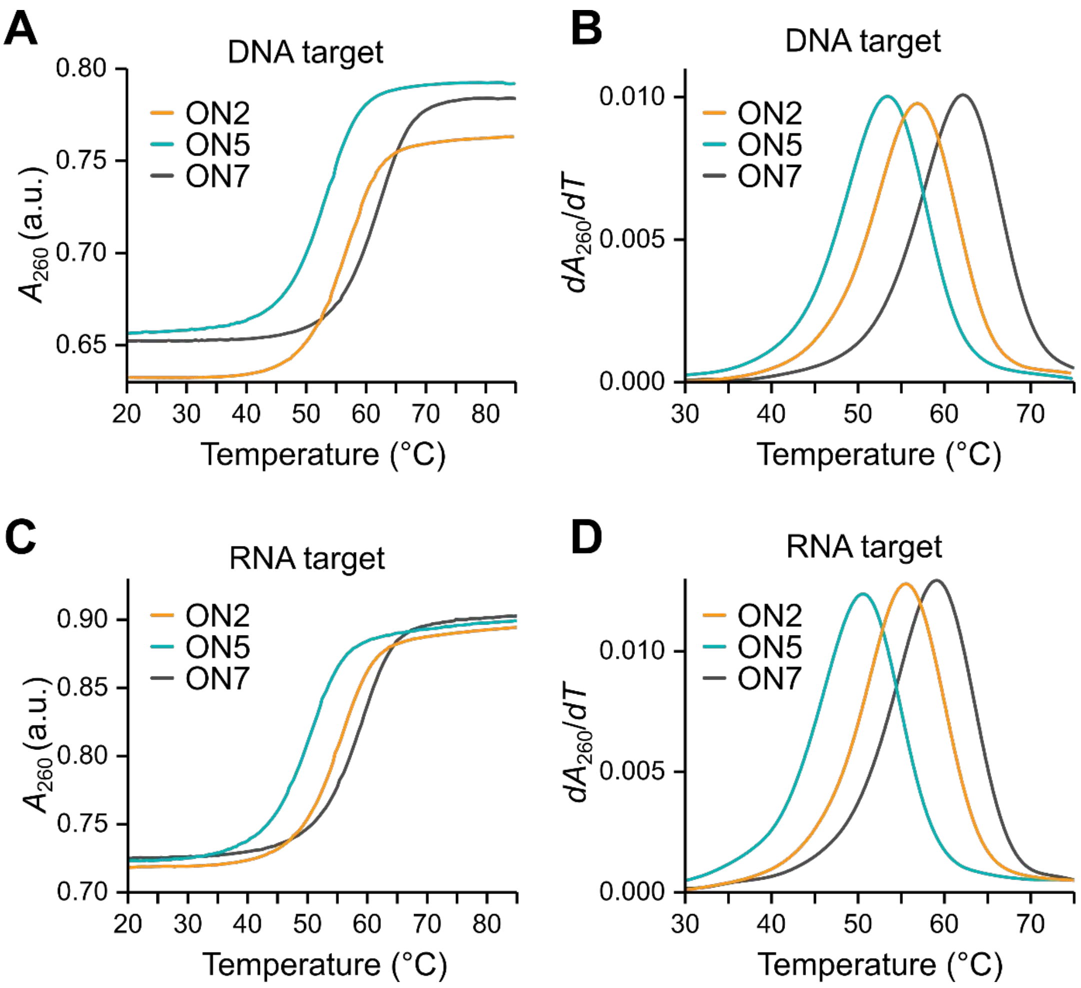

Figure S4. UV melting analysis. (A) Representative UV-melting curves of ON2, ON5 and ON7 against a complementary DNA target containing 3 µM of ON and 3 µM of the DNA target in 10 mM phosphate buffer, 200 mM NaCl, pH 7.0. Data points were taken as an average of six ramps from 20 to 85 °C. DNA target = 5′-GCTGCAAACGTCG-3′. (B) First derivatives of (A). (C) Representative UV-melting curves of ON2, ON5 and ON7 against a complementary RNA target containing 3 µM of ON and 3 µM of the RNA target in 10 mM phosphate buffer, 200 mM NaCl, pH 7.0. Data points were taken as an average of six ramps from 20 to 85 °C. RNA target = 5′-GCUGCAAACGUCG-3′. (D) First derivatives of (C).

List of oligonucleotides

Table S1. List of oligonucleotides (ONs) used in this study with calculated and found mass spectrometry (MS) data. FL = fluorescein, ^{TL} indicates the site where the natural phosphodiester (PO) is replaced by TLs **1**–**7**.

ON	Sequence (5′→3′)	Calcd (Da)	Found (Da)
ON2	CGA CGT $^{\mathbf{TL2}}$TTG CAG C	3951	3953
ON5	CGA CGT $^{\mathbf{TL5}}$TTG CAG C	3951	3953
Template 0 (PO)	GCA TTC GAG CAA CGT AAG ATC CTT ACC ACA CAA TCT CAC ACT CTG GAA TTC ACA CTG ACA ATA CTG CCG ACA CAC ATA ACC	24675	24675
Template 1 (TL**1**)	GCG TAC GAG AAC CGA ATG ATC T$^{\mathbf{TL1}}$TA GCA CAC AAT CTC ACA CTC TGG AAT TCA CAC TGA CAA TAC GTC GCA CAC AAC AAT CC	24423	24424
Template 2 (TL**2**)	GCA TTC GAG CAA CGT AAG ATC CT$^{\mathbf{TL2}}$T ACC ACA CAA TCT CAC ACT CTG GAA TTC ACA CTG ACA ATA CTG CCG ACA CAC ATA ACC	24677	24677
Template 3 (TL**3**)	GCA TTC GAG CAA CGT AAG ATC CT$^{\mathbf{TL3}}$T ACC ACA CAA TCT CAC ACT CTG GAA TTC ACA CTG ACA ATA CTG CCG ACA CAC ATA ACC	24677	24677
Template 4 (TL**4**)	GCA TTC GAG CAA CGT AAG ATC CT$^{\mathbf{TL4}}$T ACC ACA CAA TCT CAC ACT CTG GAA TTC ACA CTG ACA ATA CTG CCG ACA CAC ATA ACC	24663	24663
Template 5 (TL**5**)	GCA TTC GAG CAA CGT AAG ATC CT$^{\mathbf{TL5}}$T ACC ACA CAA TCT CAC ACT CTG GAA TTC ACA CTG ACA ATA CTG CCG ACA CAC ATA ACC	24677	24677
Template 6 (TL**6**)	GCT TAC GAC GAA GAA CGG ATC T$^{\mathbf{TL6}}$TA GCA CAC AAT CTC ACA CTC	24453	24455

	TGG AAT TCA CAC TGA CAA TAC CTC GGC CAA TAC ACA CA		
Template 7 (TL**7**)	GCA TTC GAG CAA CGT AAG ATC T^{TL7}TA GCA CAC AAT CTC ACA CTC TGG AAT TCA CAC TGA CAA TAC TGC CGA CAC ACA TAA CC	24443	24443
Template 8 (PO)	TCG TAG CAC CTT GCA GCT GAC GTT TAC ATC CAG TCT ATT GAC TAT GCA GTA ATA ACG GCC ACC CTC GCG AAC ACA CCC AAT	24736	24738
Template 9 (TL**2**)	TCG TAG CAC CTT GCA GCT GAC GT^{TL2}T TAC ATC CAG TCT ATT GAC TAT GCA GTA ATA ACG GCC ACC CTC GCG AAC ACA CCC AAT	24738	24739
Template 10 (PO)	GCG TAC GAG AAC CGA ATG ATC TTA GCA CAC AAT CTC ACA CTC TGG AAT TCA CAC TGA CAA TAC GTC GCA CAC AAC AAT CC	24451	24453
Template 11 (PO)	GCT TAC GAC GAA GAA CGG ATC TTA GCA CAC AAT CTC ACA CTC TGG AAT TCA CAC TGA CAA TAC CTC GGC CAA TAC ACA CA	24451	24453
Template 12 (PO)	GCA TTC GAG CAA CGT AAG ATC TTA GCA CAC AAT CTC ACA CTC TGG AAT TCA CAC TGA CAA TAC TGC CGA CAC ACA TAA CC	24426	24427
FL-Primer 1	FL-TGG TTA TGT GTG TCG GCA G	6442	6445
FL-Primer 2	FL-TGG ATT GTT GTG TGC GAC G	6442	6444
FL-Primer 3	FL-TTG TGT GTA TTG GCC GAG G	6442	6444
FL-Primer 4	FL-TAT TGG GTG TGT TCG CGA G	6442	6444
FP1	GCA TTC GAG CAA CGT AAG	5533	5534
FP2	GCG TAC GAG AAC CGA ATG	5558	5559
FP3	GCT TAC GAC GAA GAA CGG	5558	5559
RP1	GGT TAT GTG TGT CGG CAG	5602	5603

RP2	GGA TTG TTG TGT GCG ACG	5602	5603
RP3	TGT GTG TAT TGG CCG AGG	5602	5603
LIC-FP	AGA AGG AGA TAT AAC TGC ATT CGA GCA ACG TAA G	10557	10558
LIC-RP	GGA GAT GGG AAG TCA GGT TAT GTG TGT CGG CAG	10370	10371
Seq-FP	TAA TAC GAC TCA CTA TAG GG	6125	6126

Mass spectrometry analysis of primer extension products

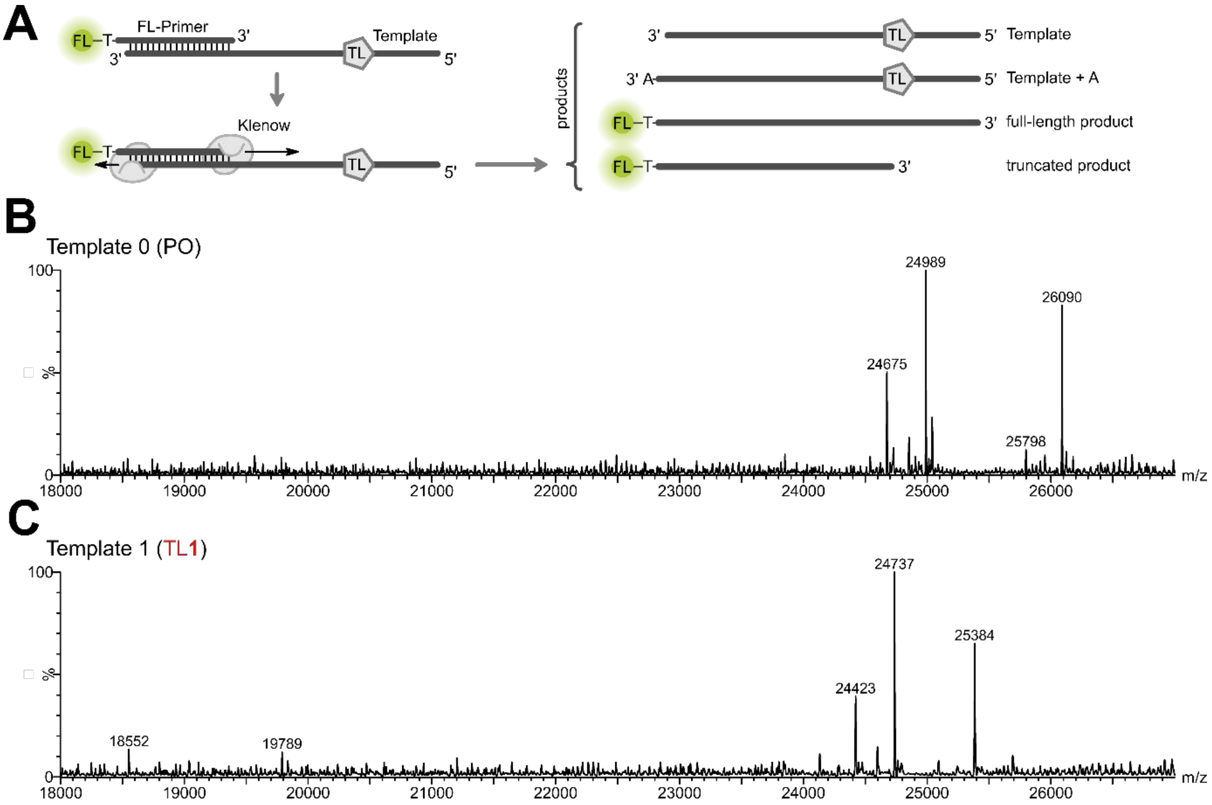

Figure S5. MS analysis of primer extension assay with Klenow fragment at 37 °C for 7.5 min. (A) Schematic overview of 3′-extension with Klenow fragment and the resulting products. Note that the single nucleotide overhang (T) of FL-primers at the 5′-end produces an addition of an A-nucleotide to the templates during the assay. (B, C) MS analyses of ONs after extension of FL-primers with Klenow fragment using (B) Template 0 (PO), FL-Primer 1 or (C) Template 1 (TL**1**), FL-Primer 2. Sequences can be found in **Table S1**. Calculated and found masses and their assignments are summarized in **Table S2**.

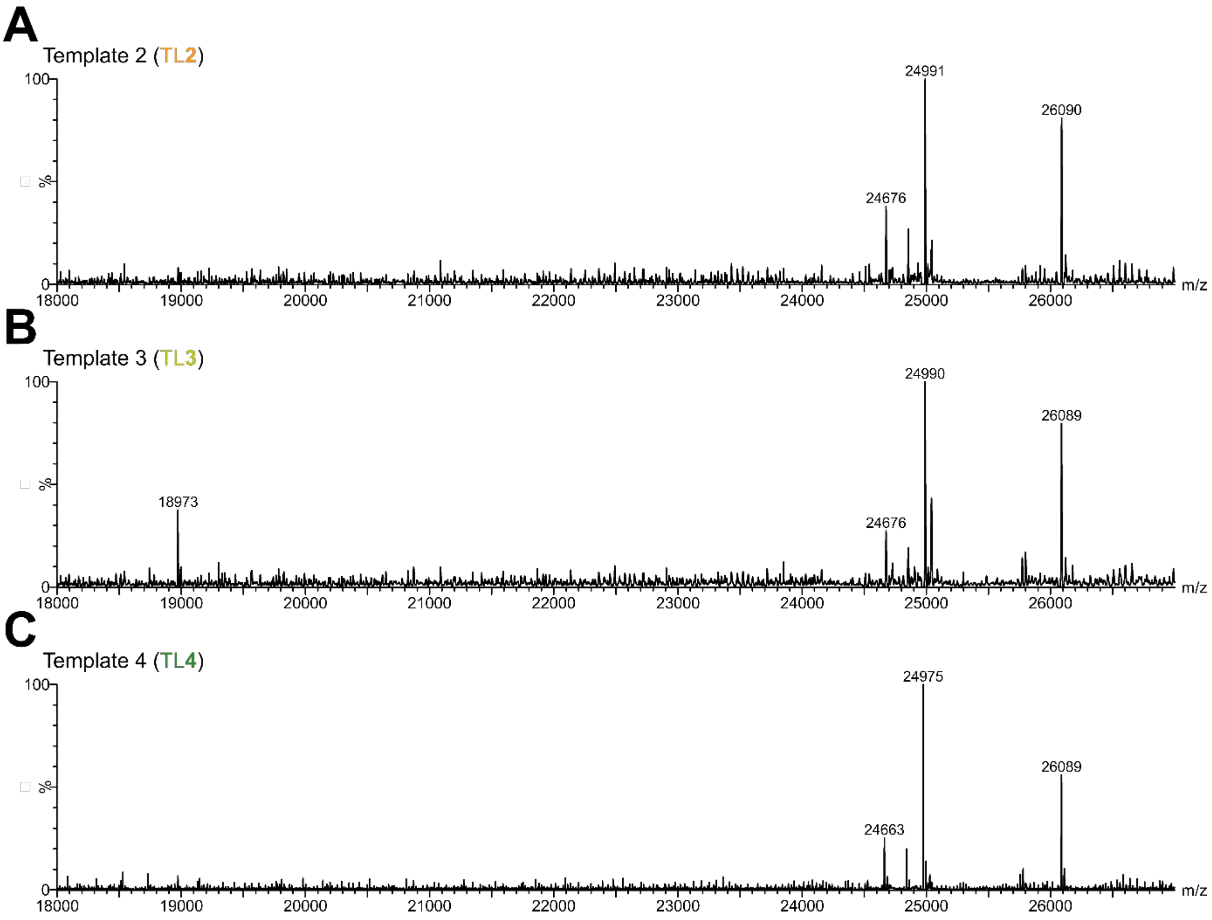

Figure S6. MS analysis of primer extension assay with Klenow fragment at 37 °C for 7.5 min. (A)–(C) MS analyses of ONs after extension of FL-primers with Klenow fragment using (A) Template 2 (TL**2**), FL-Primer 1; (B) Template 3 (TL**3**), FL-Primer 1 or (C) Template 4 (TL**4**), FL-Primer 1. Sequences can be found in **Table S1**. Calculated and found masses and their assignments are summarized in **Table S2**.

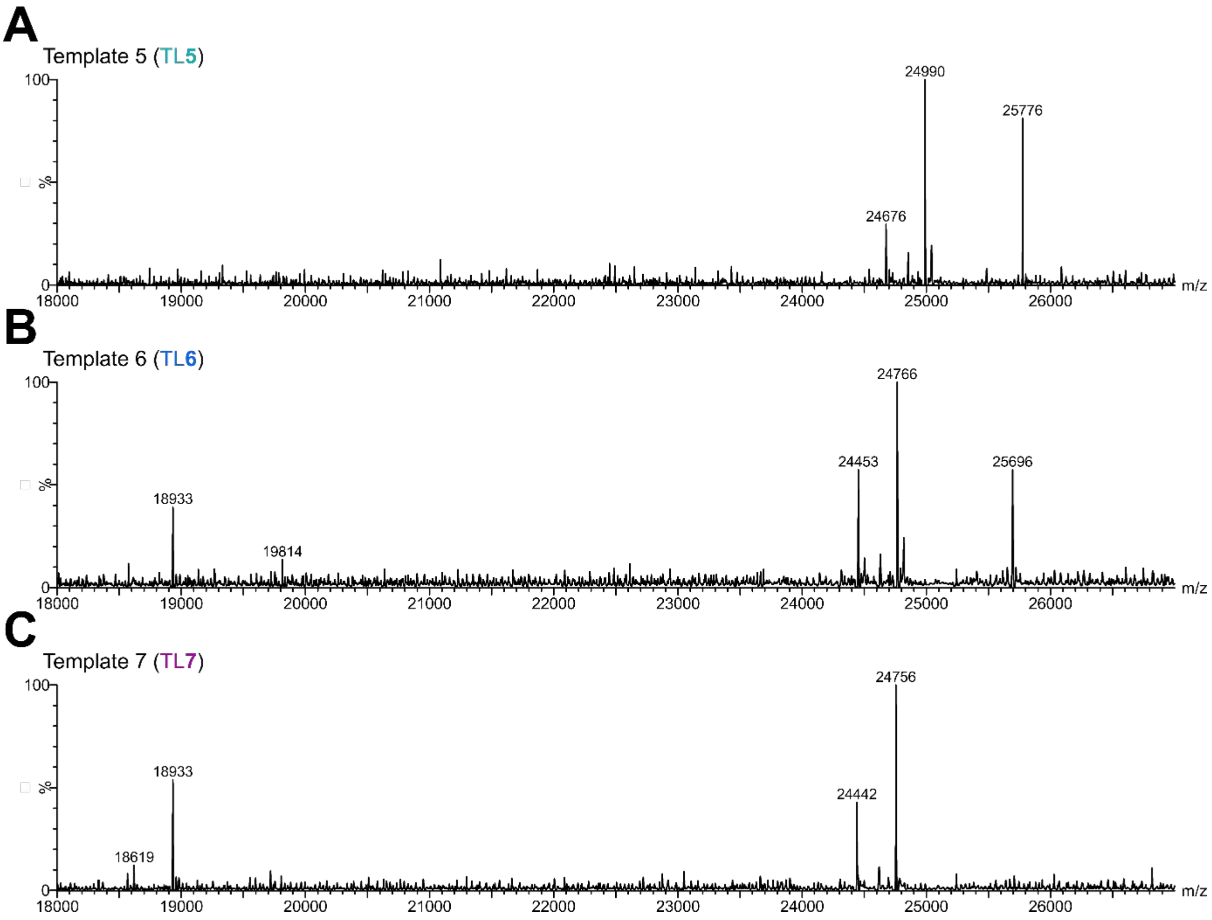

Figure S7. MS analysis of primer extension assay with Klenow fragment at 37 °C for 7.5 min. (A)–(C) MS analyses of ONs after extension of FL-primers with Klenow fragment using (A) Template 5 (TL**5**), FL-Primer 1; (B) Template 6 (TL**6**), FL-Primer 3 or (D) Template 7 (TL**7**), FL-Primer 1. Sequences can be found in **Table S1**. Calculated and found masses and their assignments are summarized in **Table S2**.

Table S2. List of calculated and found ON masses after primer extension with Klenow fragment at 37 °C for 7.5 min.

Template	Calcd (Da)	Found (Da)	Comment
Template 0 (PO)	26089	26090	Full-length product
	24988	24989	Template 0 + A
	24675	24675	Template 0
Template 1 (TL**1**)	25382	25384	Full-length product with –A deletion
	24736	24737	Template 1 + A
	24423	24423	Template 1
Template 2 (TL**2**)	26089	26090	Full-length product
	24990	24991	Template 2 + A
	24677	24676	Template 2
Template 3 (TL**3**)	26089	26089	Full-length product
	24990	24990	Template 3 + A
	24677	24676	Template 3
	18972	18973	Truncated product; stopped directly before TL**3**
Template 4 (TL**4**)	26089	26089	Full-length product
	24976	24975	Template 4 + A
	24663	24663	Template 4
Template 5 (TL**5**)	25776	25776	Full-length product with –A deletion
	24990	24990	Template 5 + A
	24677	24676	Template 5
Template 6 (TL**6**)	25695	25696	Full-length product
	24766	24766	Template 6 + A
	24453	24453	Template 6
	18932	18933	Truncated product; stopped directly before TL**6**
Template 7 (TL**7**)	24756	24756	Template 7 + A
	24443	24442	Template 7
	18932	18933	Truncated product; stopped directly before TL**7**
	18619	18619	Truncated product; stopped one nucleotide before TL**7**

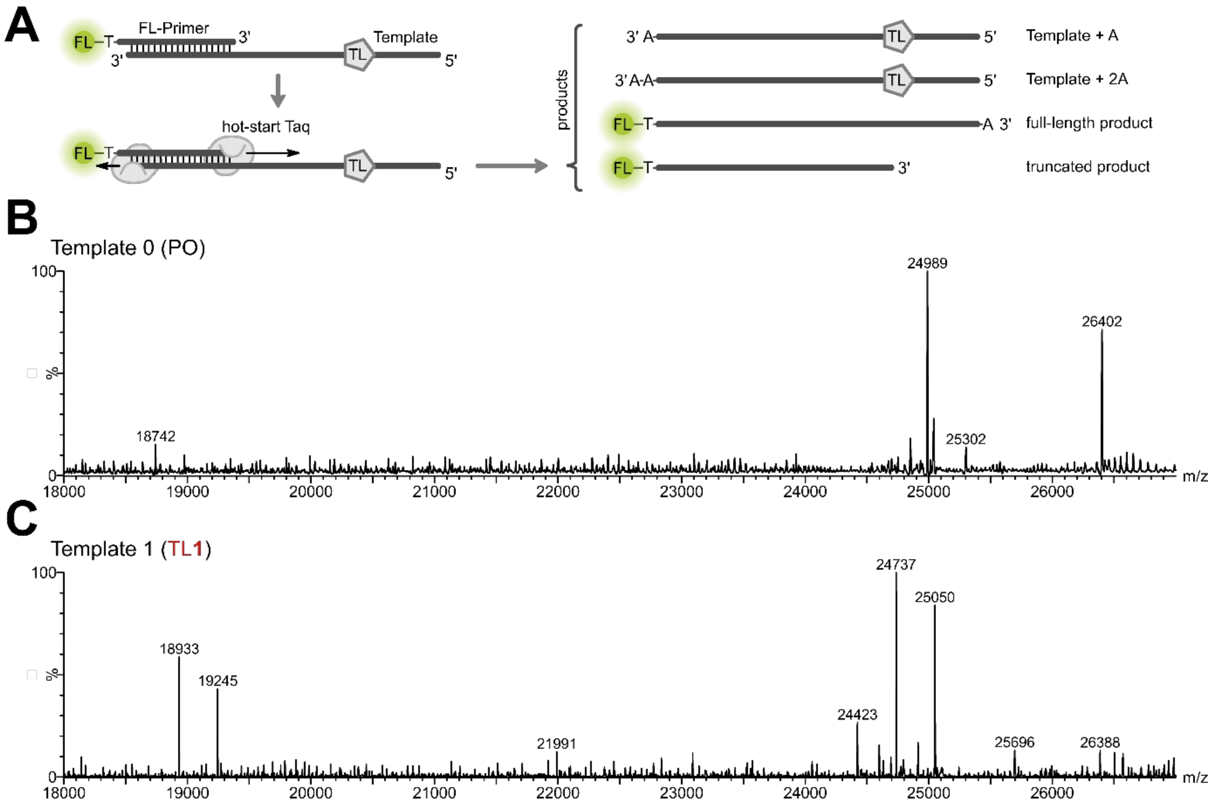

Figure S8. MS analysis of primer extension assay with hot-start Taq polymerase at 60 °C for 160 min. (A) Schematic overview of 3′-extension with hot-start Taq polymerase and the resulting products. Note that the single nucleotide overhang (T) of FL-primers at the 5′-end produces an addition of an A-nucleotide to the templates during the assay. The formation of a 3′-A overhang by hot-start Taq is included in the products. (B, C) MS analyses of ONs after extension of FL-primers with hot-start Taq polymerase using (B) Template 0 (PO), FL-Primer 1 or (C) Template 1 (TL**1**), FL-Primer 2. Sequences can be found in **Table S1**. Calculated and found masses and their assignments are summarized in **Table S3**.

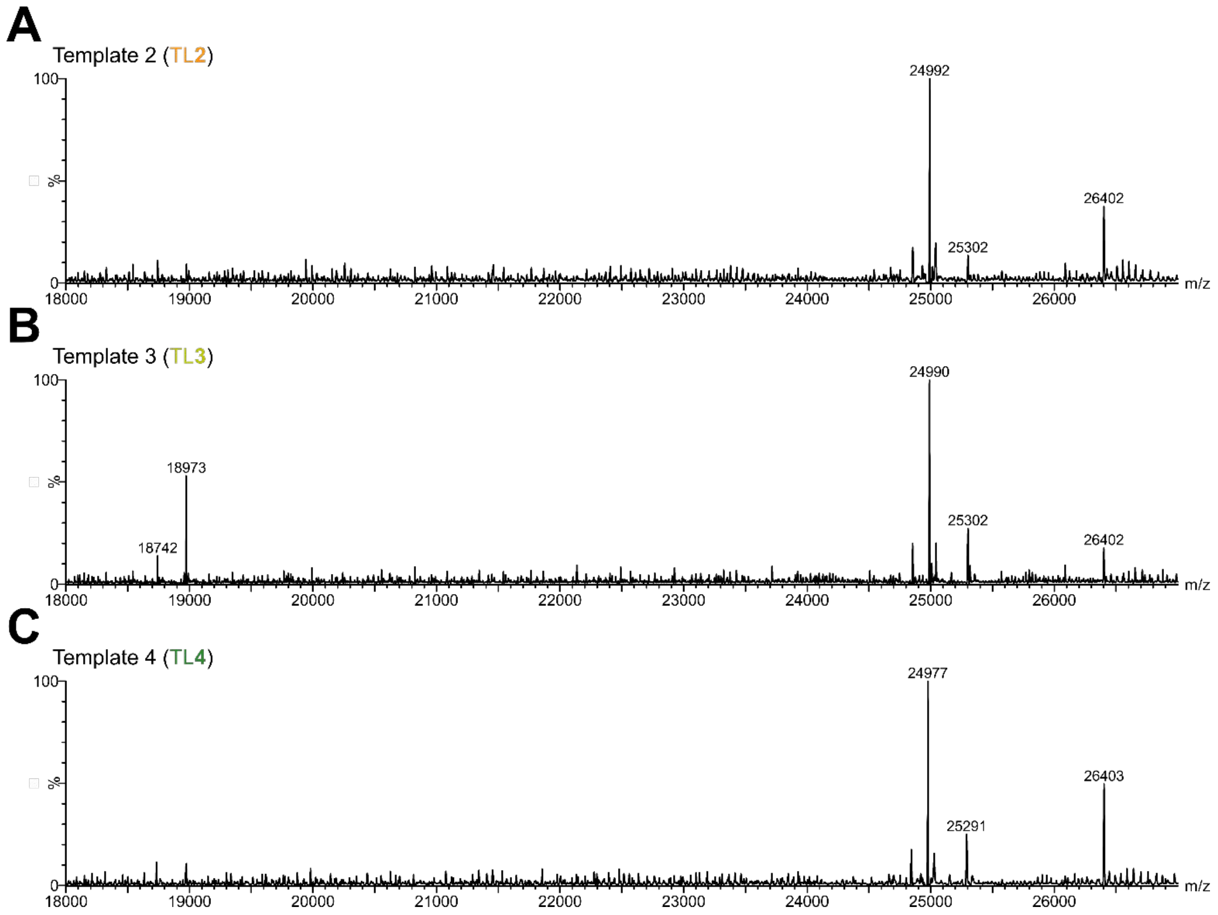

Figure S9. MS analysis of primer extension assay with hot-start Taq polymerase at 60 °C for 160 min. (A)–(C) MS analyses of ONs after extension of FL-primers with hot-start Taq polymerase using (A) Template 2 (TL**2**), FL-Primer 1; (B) Template 3 (TL**3**), FL-Primer 1 or (C) Template 4 (TL**4**), FL-Primer 1. Sequences can be found in **Table S1**. Calculated and found masses and their assignments are summarized in **Table S3**.

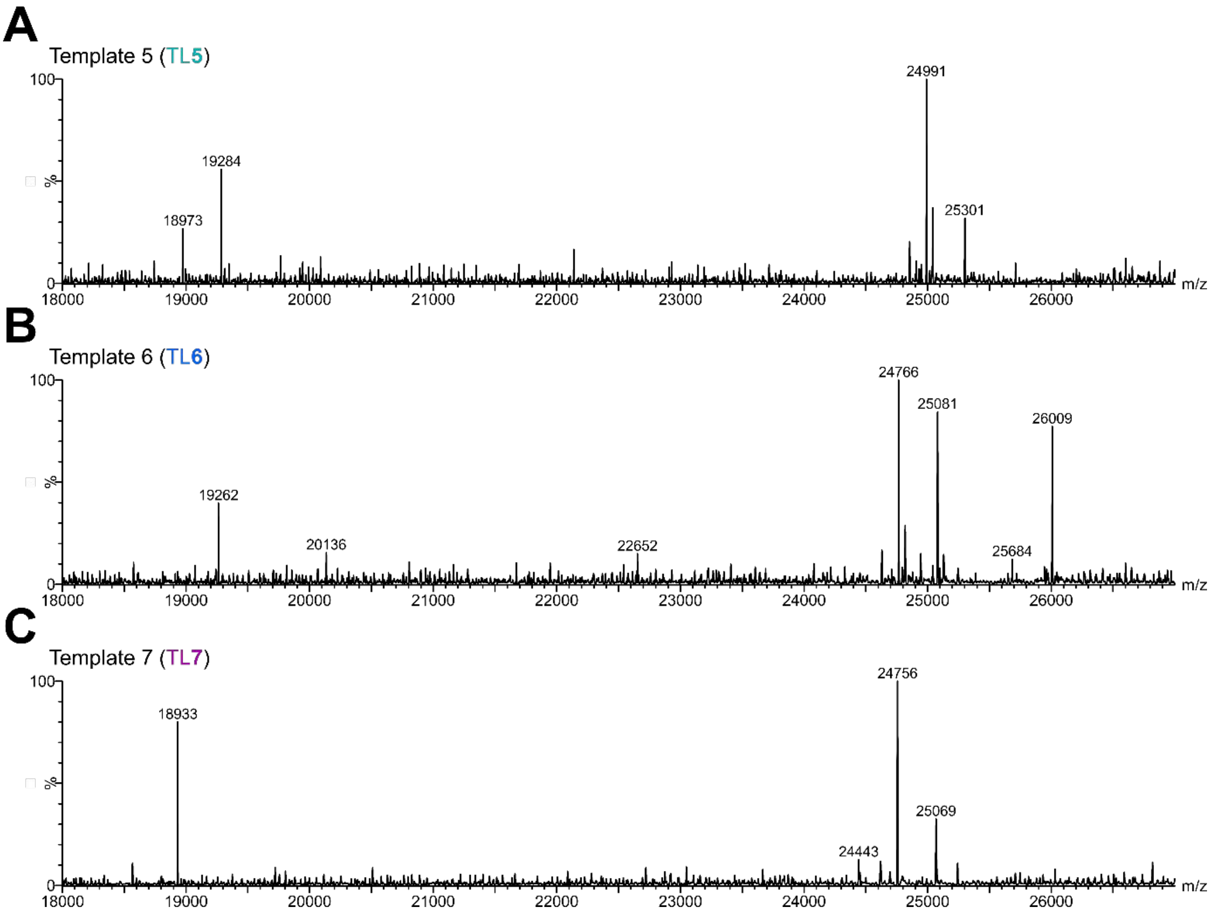

Figure S10. MS analysis of primer extension assay with hot-start Taq polymerase at 60 °C for 160 min. (A)–(C) MS analyses of ONs after extension of FL-primers with hot-start Taq polymerase using (A) Template 5 (TL**5**), FL-Primer 1; (B) Template 6 (TL**6**), FL-Primer 3 or (D) Template 7 (TL**7**), FL-Primer 1. Sequences can be found in **Table S1**. Calculated and found masses and their assignments are summarized in **Table S3**.

Table S3. List of calculated and found ON masses after primer extension with hot-start Taq polymerase at 60 °C for 160 min.

Template	Calcd (Da)	Found (Da)	Comment
Template 0 (PO)	26402	26402	Full-length product (including + 3′-A overhang adduct)
	25302	25302	Template 0 + 2 × A
	24988	24989	Template 0 + A
Template 1 (TL**1**)	25695	25696	Full-length product (without 3′-A overhang adduct or with – A deletion)
	25049	25050	Template 1 + 2 × A
	24736	24737	Template 1 + A
	24423	24423	Template 1
	19245	19245	Truncated product; stopped directly after TL**1**
	18932	18933	Truncated product; stopped directly before TL**1**
Template 2 (TL**2**)	26402	26402	Full-length product (including + 3′-A overhang adduct)
	25304	25302	Template 2 + 2 × A
	24990	24992	Template 2 + A
Template 3 (TL**3**)	26402	26402	Full-length product (including + 3′-A overhang adduct)
	25304	25302	Template 3 + 2 × A
	24990	24990	Template 3 + A
	18972	18973	Truncated product; stopped directly before TL**3**
Template 4 (TL**4**)	26402	26403	Full-length product (including + 3′-A overhang adduct)
	25290	25291	Template 4 + 2 × A
	24976	24977	Template 4 + A
Template 5 (TL**5**)	25304	25301	Template 5 + 2 × A
	24990	24991	Template 5 + A
	19285	19284	Truncated product; stopped directly after TL**5**
	18972	18973	Truncated product; stopped directly before TL**5**
Template 6 (TL**6**)	26009	26009	Full-length product (including + 3′-A overhang adduct)
	25080	25081	Template 6 + 2 × A
	24766	24766	Template 6 + A
	19261	19262	Truncated product; stopped directly after TL**6** but with A→G mutation
Template 7 (TL**7**)	25070	25069	Template 7 + 2 × A
	24756	24756	Template 7 + A
	24443	24443	Template 7
	18932	18933	Truncated product; stopped directly before TL**7**

Additional primer extension experiments

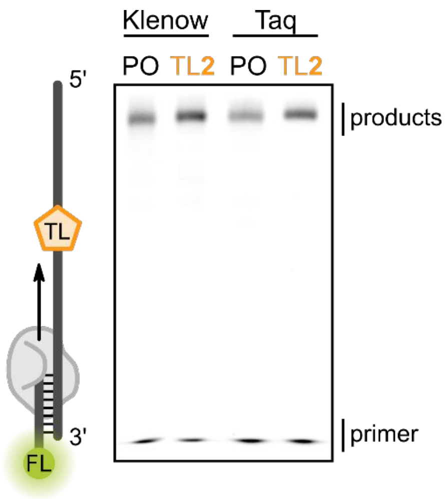

Figure S11. Primer extension experiments with alternative templates. Polymerase-mediated extension of FL-Primer 4 using either unmodified Template 8 (PO) or modified Template 9 (TL**2**). Primer extension by Klenow fragment (left) was performed at 37 °C for 7.5 min while primer extension by hot-start Taq polymerase (right) was performed at 60 °C for 160 min. Extension products were separated by 8% denaturing polyacrylamide gel electrophoresis (PAGE) and the FL-labelled ONs were visualized (λ_{ex} = 460 nm, λ_{em} = 516–600 nm). Sequences can be found in **Table S1**.

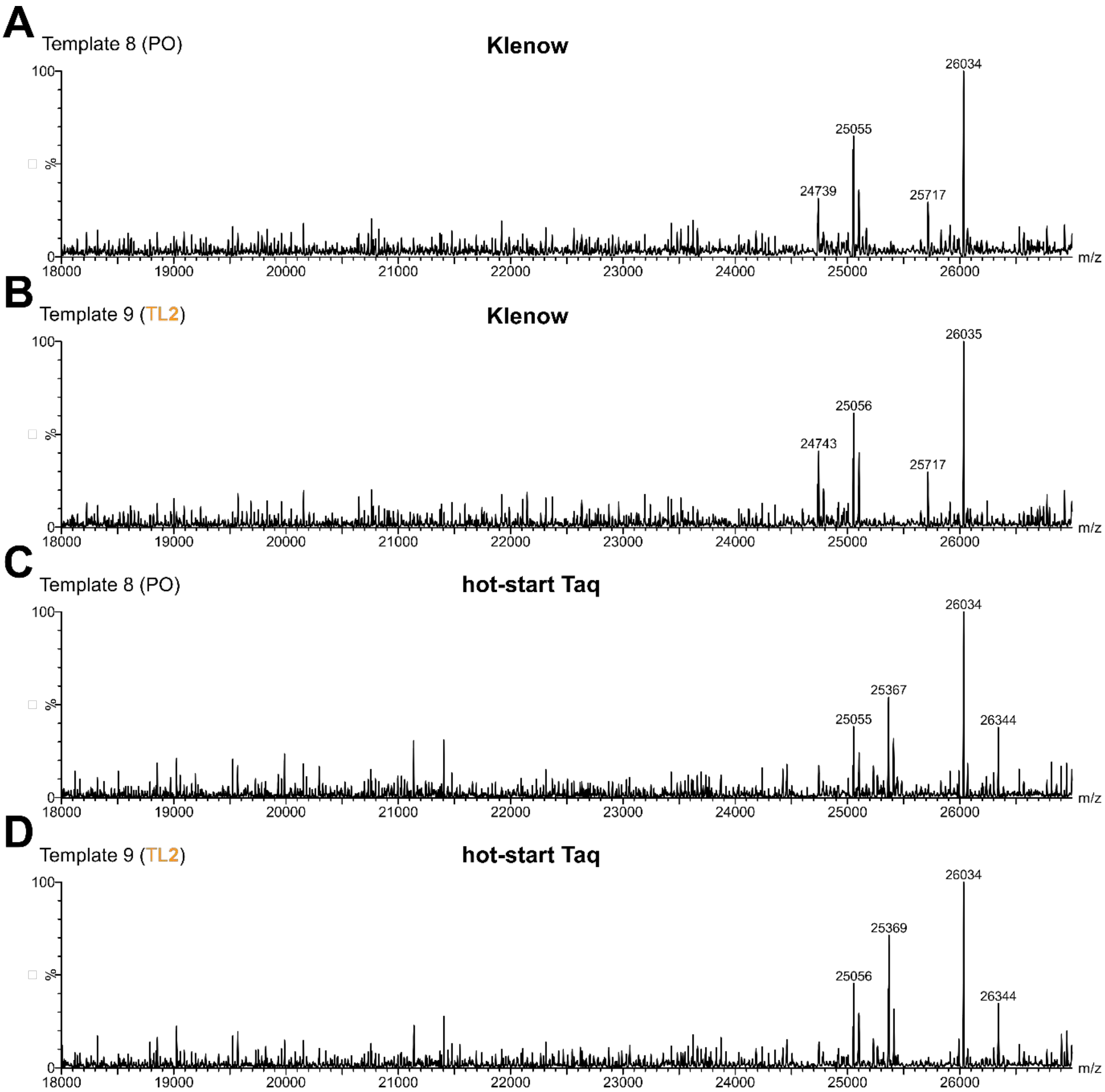

Figure S12. MS analyses of ONs after extension of FL-Primer 4 using alternative templates: Template 8 (PO) or Template 9 (TL**2**). (A, B) Klenow fragment at 37 °C for 7.5 min. (C, D) hot-start Taq polymerase at 60 °C for 160 min. Sequences can be found in **Table S1**. Calculated and found masses and their assignments are summarized in **Table S4**.

Table S4. List of calculated and found ON masses after primer extension with Klenow fragment at 37 °C for 7.5 min or hot-start Taq polymerase at 60 °C for 160 min.

	Template	Calcd (Da)	Found (Da)	Comment
Klenow	Template 8 (PO)	26031	26034	Full-length product
		25717	25717	Full-length product – A[a]
		25049	25055	Template 8 + A
		24736	24739	Template 8
	Template 9 (TL**2**)	26031	26035	Full-length product
		25717	25717	Full-length product – A[a]
		25051	25056	Template 9 + A
		24738	24743	Template 9
hot-start Taq	Template 8 (PO)	26344	26344	Full-length product (including + 3′-A overhang adduct)
		26031	26034	Full-length product (without the 3′-A overhang adduct)[b]
		25363	25367	Template 8 + 2 × A
		25049	25055	Template 8 + A
	Template 9 (TL**2**)	26344	26344	Full-length product (including + 3′-A overhang adduct)
		26031	26034	Full-length product (without the 3′-A overhang adduct)[b]
		25365	25369	Template 9 + 2 × A
		25051	25056	Template 9 + A

[a] distinct peaks with a missing A-nucleotide were detected for the unmodified Template 8 and isosequential TL**2**-modified Template 9, both having a 5′-T. This suggests that the missing A-nucleotide in the replicon results in both cases from incomplete replication without addition of the 3′-terminal A-nucleotide (complementary to the 5′-T of the templates) and is thus determined by the template sequence and not by the TL**2** modification.

[b] major peaks without the Taq-characteristic 3′-A overhang were detected for the unmodified Template 8 and isosequential TL**2**-modified Template 9. This suggests that formation of a 3′-A overhang is suppressed in both cases and not specific to the TL**2** modification.

qPCR Experiments

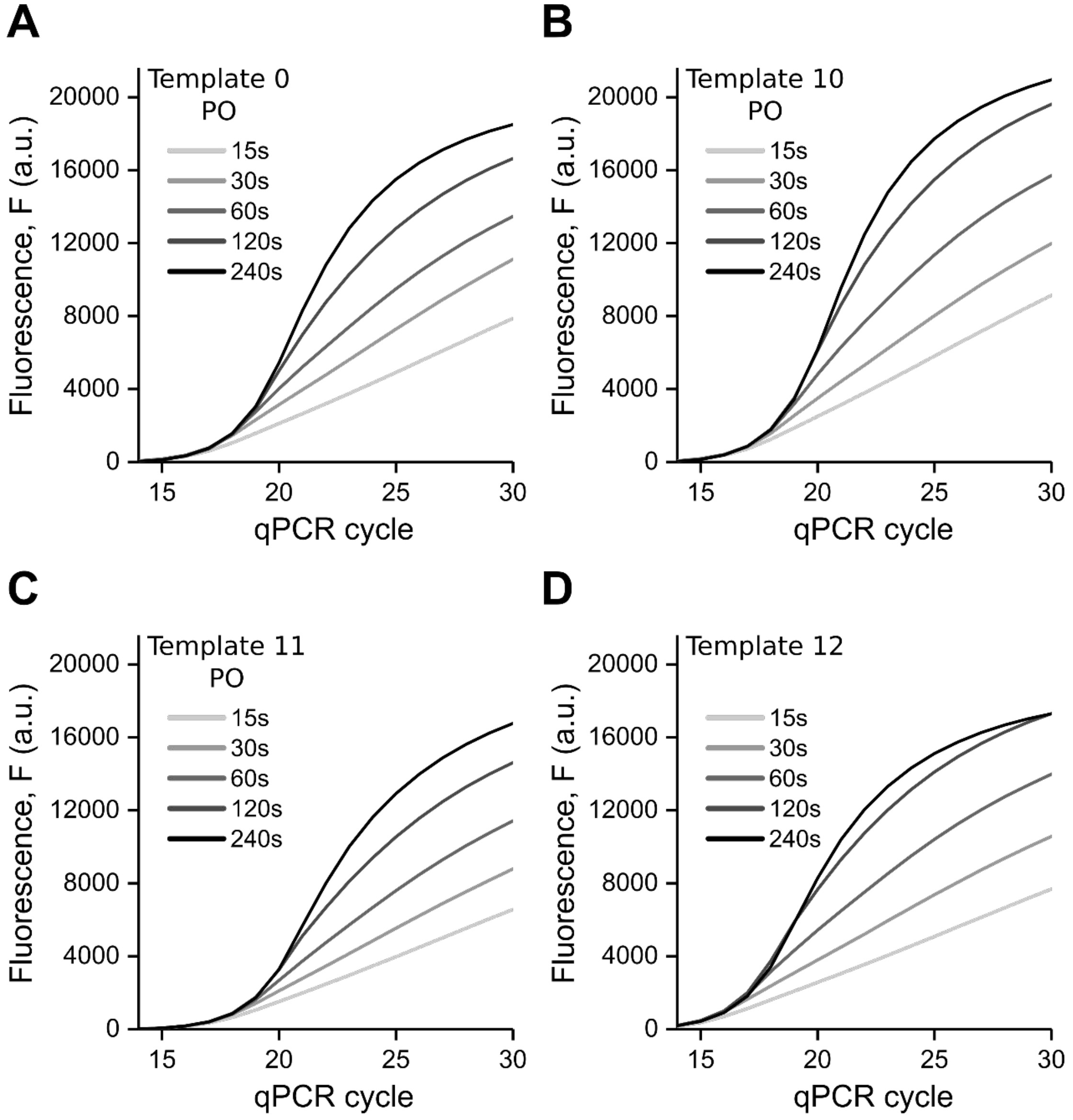

Figure S13. Representative qPCR amplifications of Template 0 and Templates 10–12 by hot-start Taq polymerase with different extension times. Fluorescence emission was monitored at the end of each amplification cycle. The extension time during the qPCR cycle was varied from 15–240 s. Templates, forward and reverse primers (FP, RP) were the following: (A) Template 0 (PO), FP1, RP1; (B) Template 10 (PO), FP2, RP2; (C) Template 11 (PO), FP3, RP3 and (D) Template 12 (PO), FP1, RP1. Sequences can be found in **Table S1**. Enlarged sections for each panel showing the threshold used for the C_t calculations are shown in **Figure S16**. Formation of a single product was demonstrated by T_m measurements for each experiment and are shown in **Figure S19**.

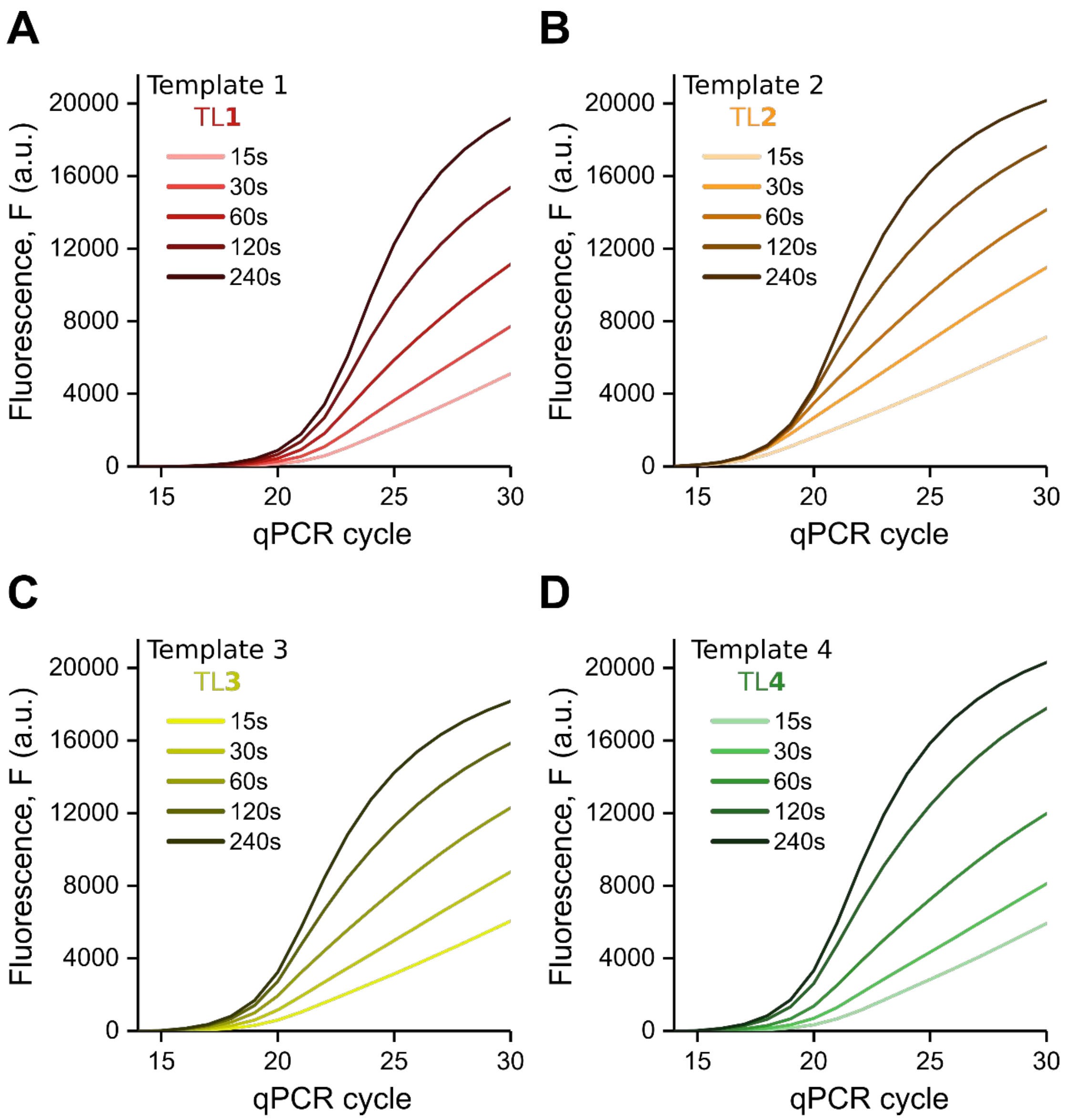

Figure S14. Representative qPCR amplifications of Templates 1–4 by hot-start Taq polymerase with different extension times. Fluorescence emission was monitored at the end of each amplification cycle. The extension time during the qPCR cycle was varied from 15–240 s. Templates, FP and RP were the following: (A) Template 1 (TL**1**), FP2, RP2; (B) Template 2 (TL**2**), FP1, RP1; (C) Template 3 (TL**3**), FP1, RP1 and (D) Template 4 (TL**4**), FP1, RP1. Sequences can be found in **Table S1**. Enlarged sections for each panel showing the threshold used for the C_t calculations are shown in **Figure S17**. Formation of a single product was demonstrated by T_m measurements for each experiment and are shown in **Figure S20**.

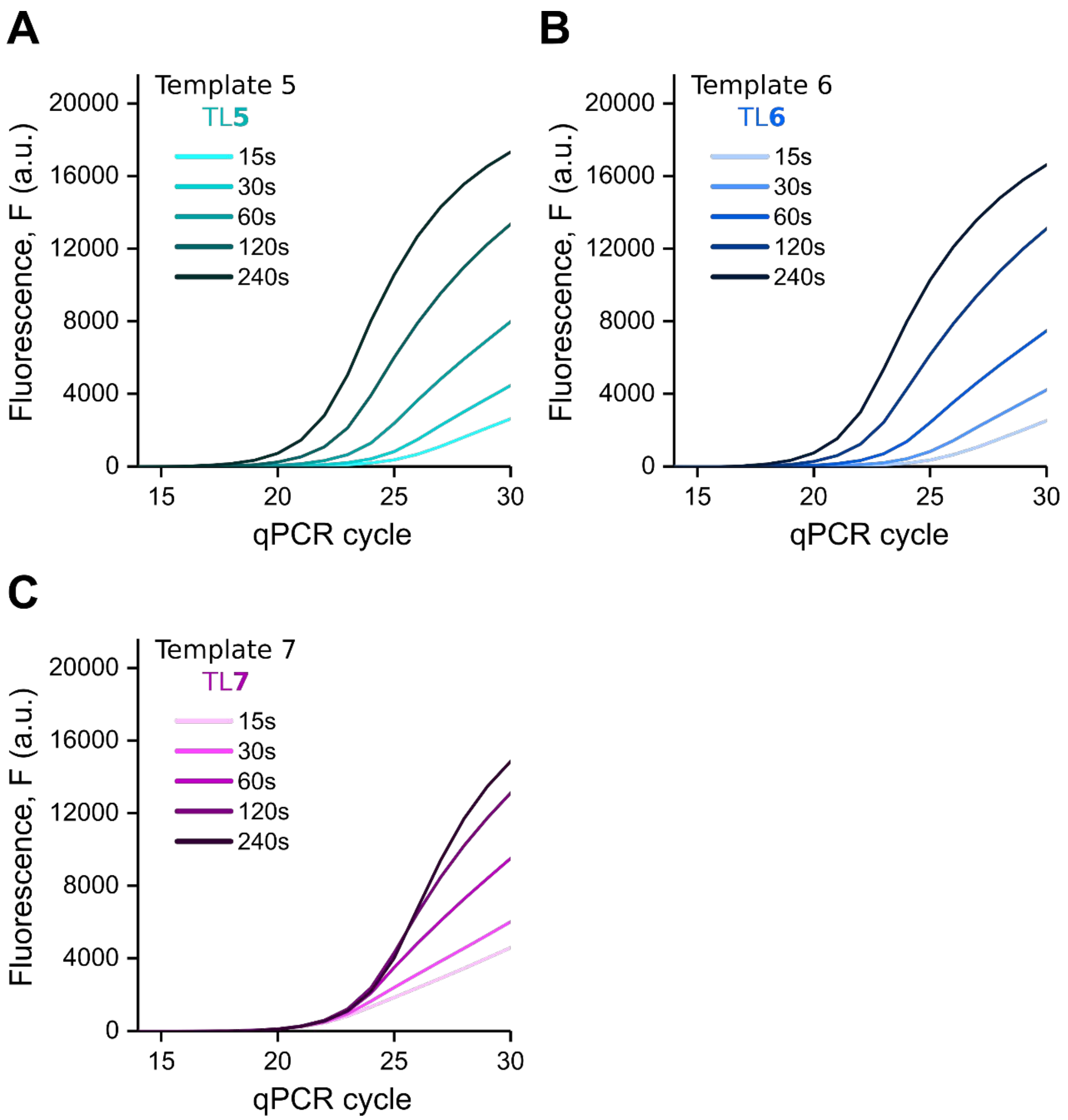

Figure S15. Representative qPCR amplifications of Templates 5–7 by hot-start Taq polymerase with different extension times. Fluorescence emission was monitored at the end of each amplification cycle. The extension time during the qPCR cycle was varied from 15–240 s. Templates, FP and RP were the following: (A) Template 5 (TL**5**), FP1, RP1; (B) Template 6 (TL**6**), FP3, RP3 and (C) Template 7 (TL**7**), FP1, RP1. Sequences can be found in **Table S1**. Enlarged sections for each panel showing the threshold used for the C_t calculations are shown in **Figure S18**. Formation of a single product was demonstrated by T_m measurements for each experiment and are shown in **Figure S21**.

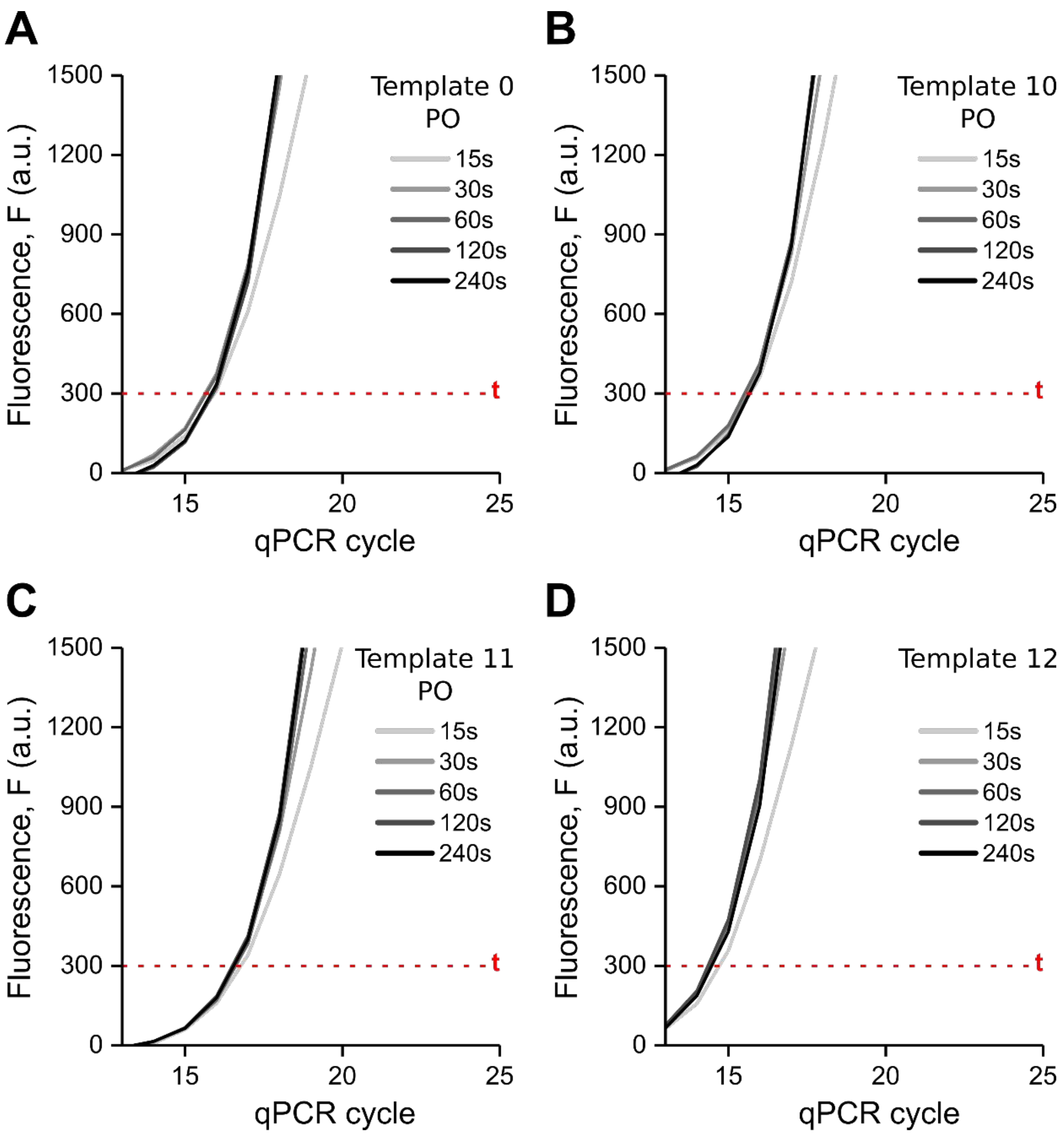

Figure S16. Enlarged, representative sections of qPCR amplifications of Template 0 and Templates 10–12 by hot-start Taq polymerase with different extension times, showing the threshold (t) at F = 300 (a.u.) as a red dotted line. (A)–(D) correspond to qPCR samples from (A)–(D) in **Figure S13**.

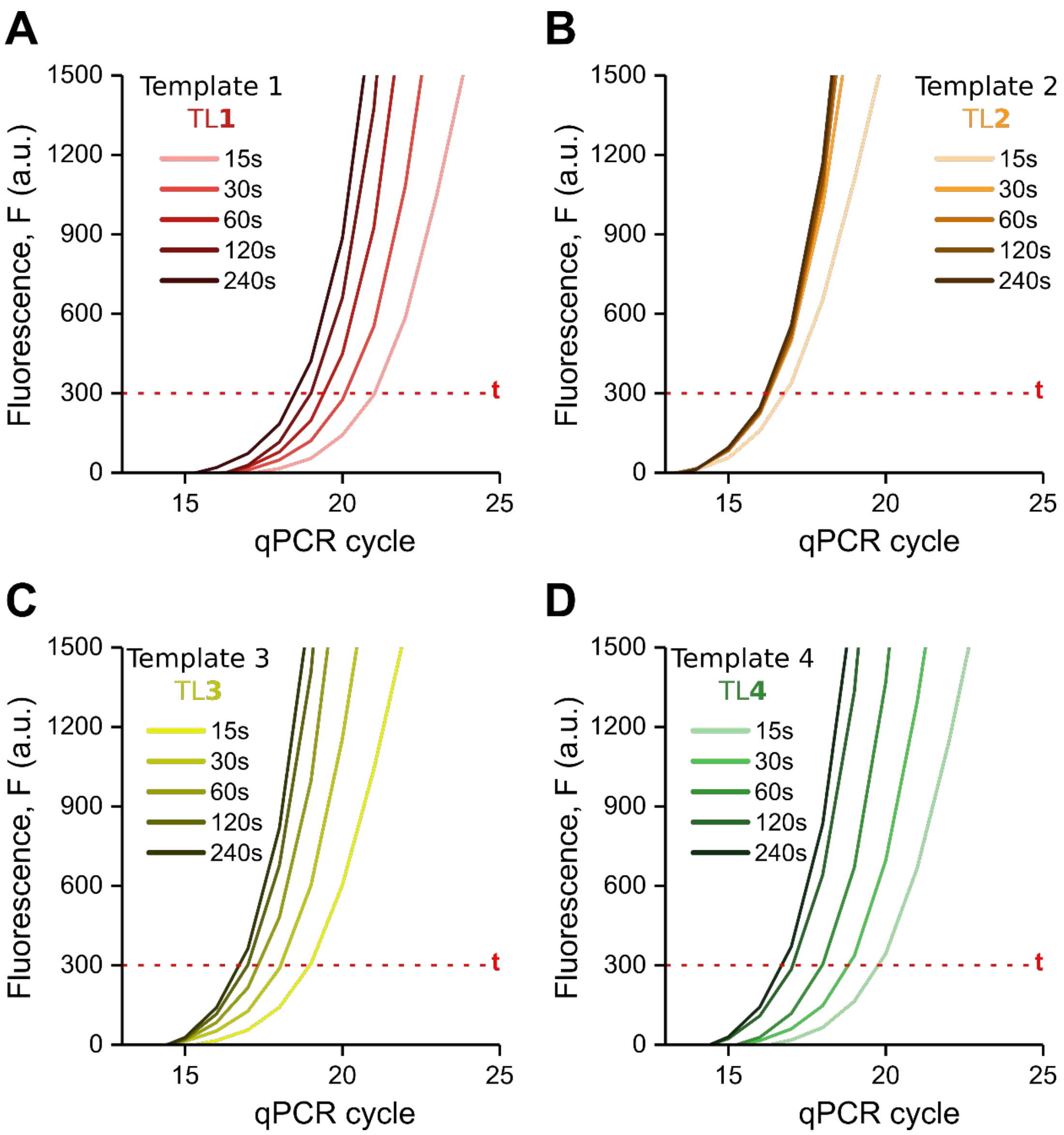

Figure S17. Enlarged, representative sections of qPCR amplifications of Templates 1–4 by hot-start Taq polymerase with different extension times, showing the threshold (t) at F = 300 (a.u.) as a red dotted line. (A)–(D) correspond to qPCR samples from (A)–(D) in **Figure S14**.

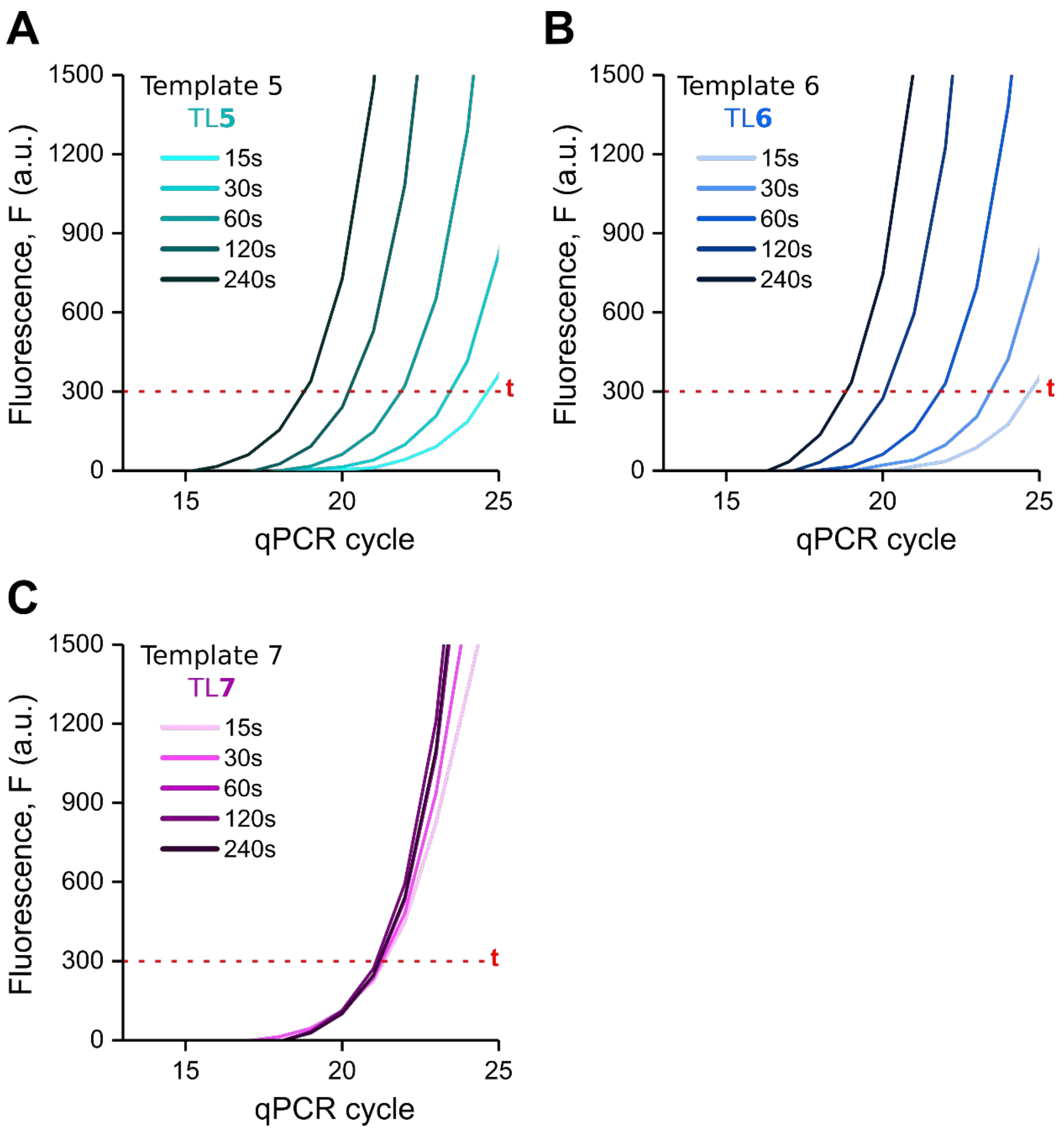

Figure S18. Enlarged, representative sections of qPCR amplifications of Templates 5–7 by hot-start Taq polymerase with different extension times, showing the threshold (t) at F = 300 (a.u.) as a red dotted line. (A)–(D) correspond to qPCR samples from (A)–(D) in **Figure S15**.

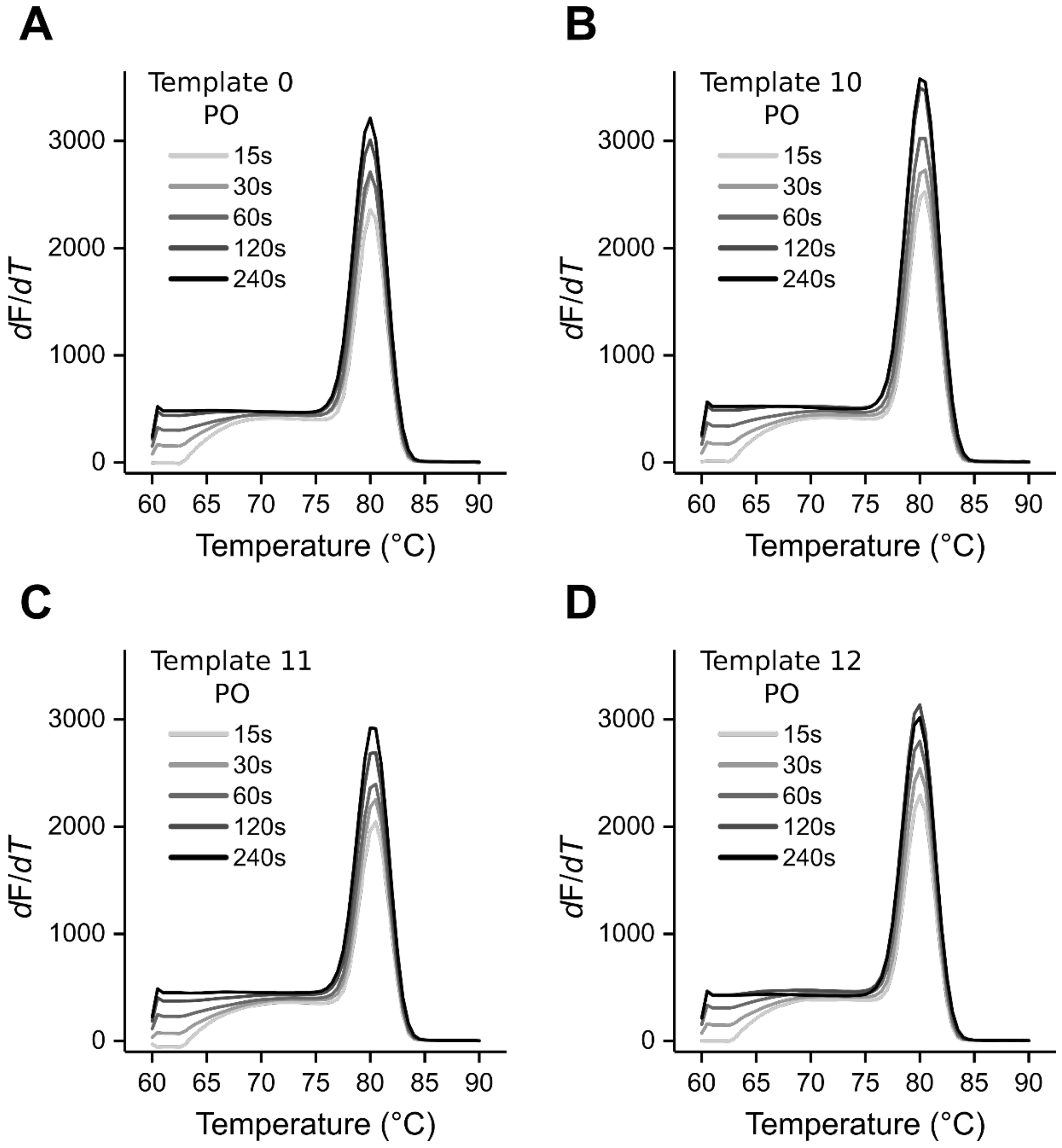

Figure S19. Melting curves of qPCR products from **Figure S13**. The first derivatives (dF/dT) of fluorescence emissions from post-qPCR samples are shown as a function of T. Samples were taken after qPCRs with hot-start Taq polymerase and fluorescence emission was measured while heating the samples from 60 to 90 °C with a ramp of 6 °C/min (fluorescence measurement in 0.5 °C intervals). Single peaks confirm formation of a single product. (A)–(D) correspond to qPCR samples from (A)–(D) in **Figure S13**.

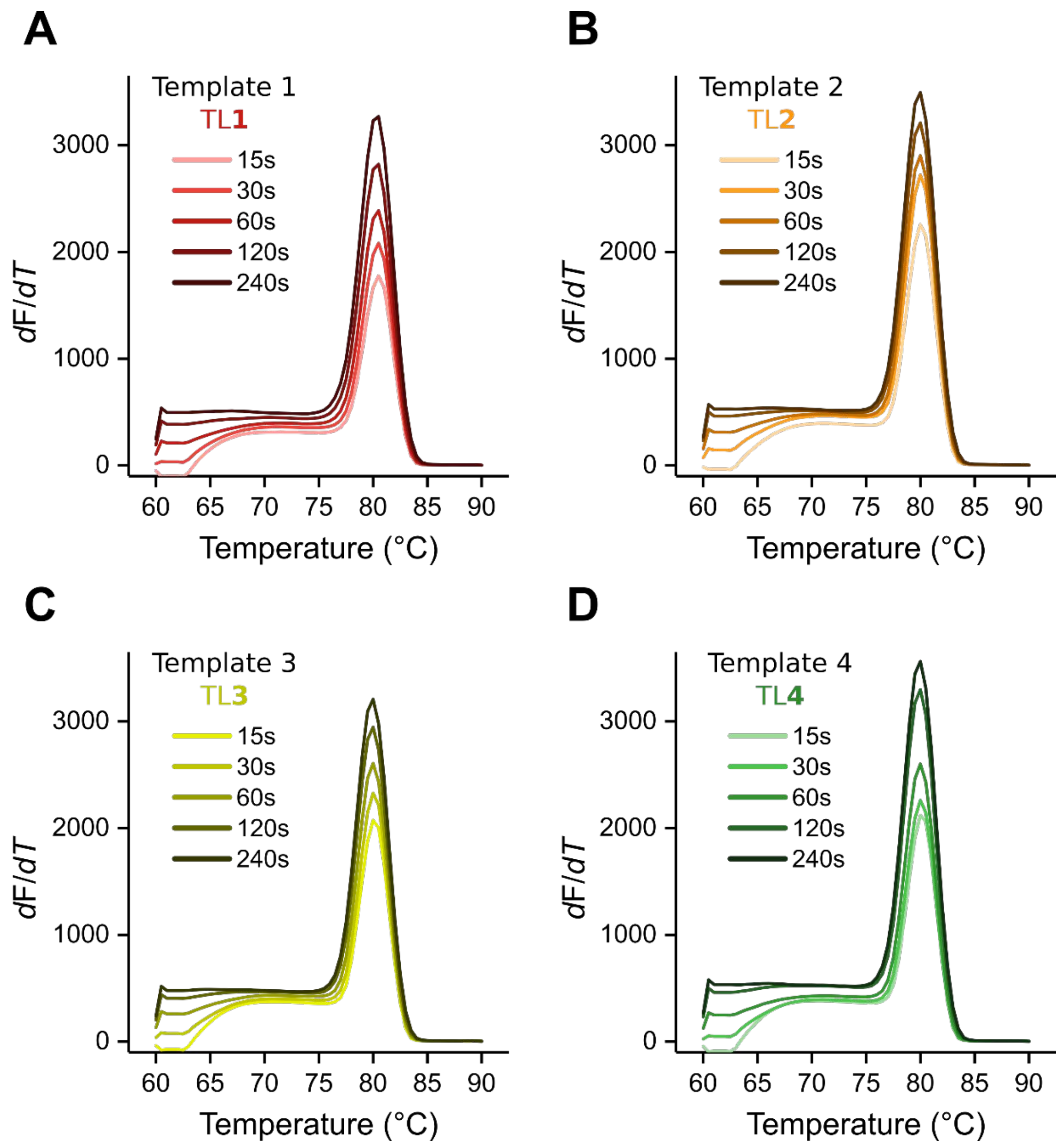

Figure S20. Melting curves of qPCR products from **Figure S14**. The first derivatives (*d*F/*dT*) of fluorescence emissions from post-qPCR samples are shown as a function of *T*. Samples were taken after qPCRs with hot-start Taq polymerase and fluorescence emission was measured while heating the samples from 60 to 90 °C with a ramp of 6 °C/min (fluorescence measurement in 0.5 °C intervals). Single peaks confirm formation of a single product. (A)–(D) correspond to qPCR samples from (A)–(D) in **Figure S14**.

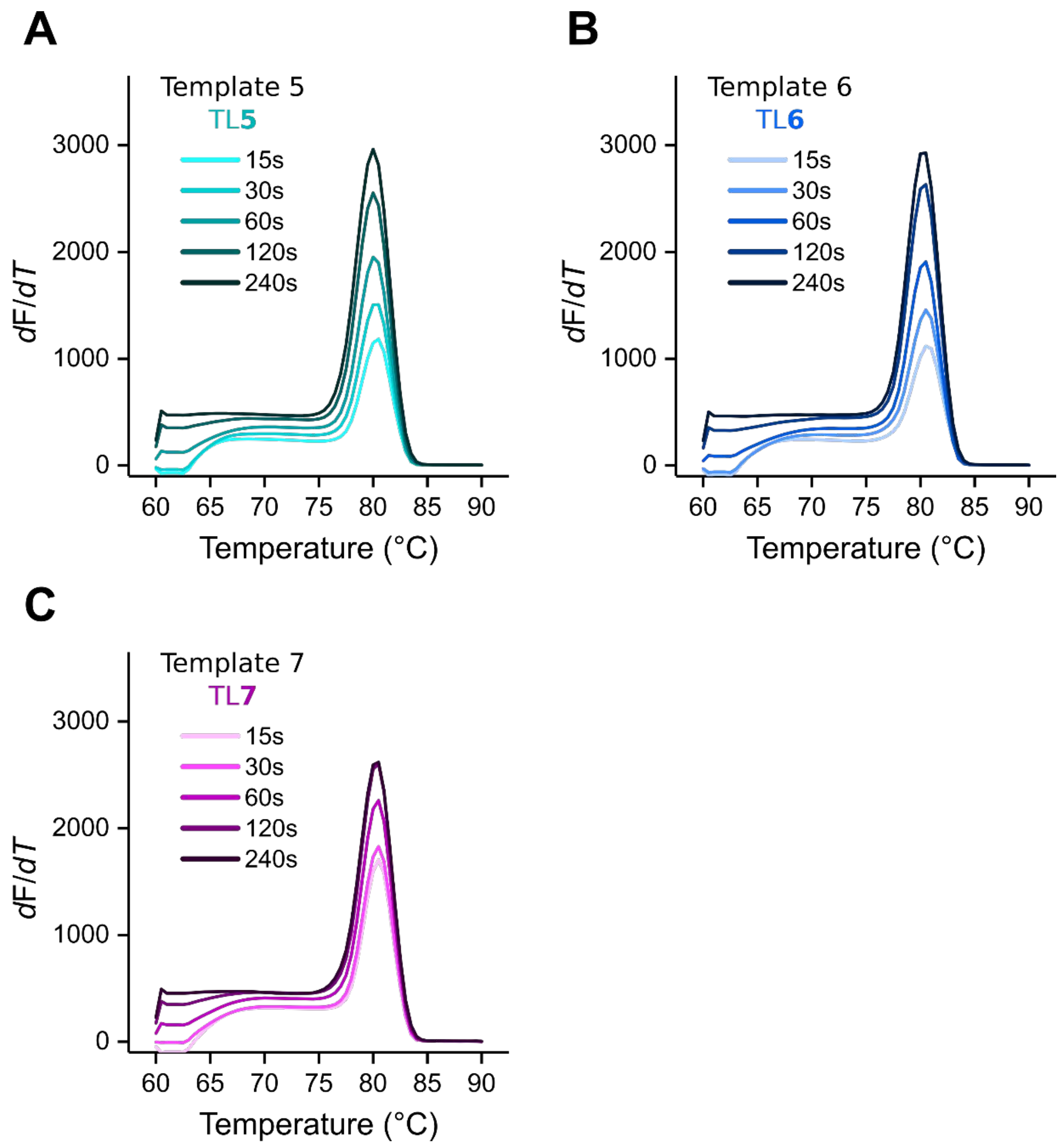

Figure S21. Melting curves of qPCR products from **Figure S15**. The first derivatives (dF/dT) of fluorescence emissions from post-qPCR samples are shown as a function of T. Samples were taken after qPCRs with hot-start Taq polymerase and fluorescence emission was measured while heating the samples from 60 to 90 °C with a ramp of 6 °C/min (fluorescence measurement in 0.5 °C intervals). Single peaks confirm formation of a single product. (A)–(C) correspond to qPCR samples from (A)–(C) in **Figure S15**.

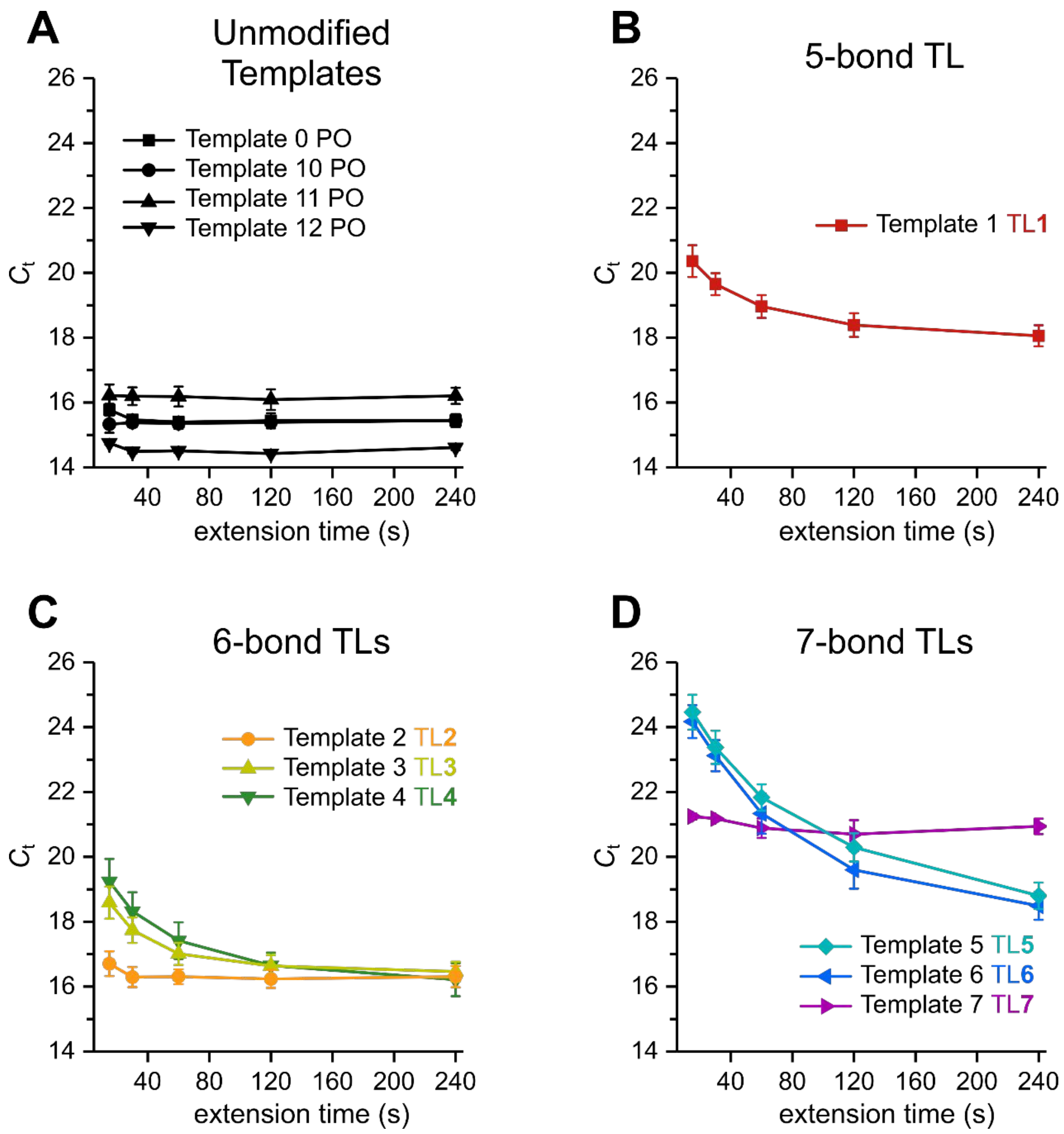

Figure S22. Threshold cycles (C_ts) from qPCRs with hot-start Taq polymerase with different extension times from 15–240 s. C_ts were determined from qPCRs with a threshold fluorescence set to F = 300 (a.u.). Data is presented as the average from triplicates ± standard error of the mean. (A) Unmodified templates: Template 0 (PO), FP1, RP1; Template 10 (PO), FP2, RP2; Template 11 (PO), FP3, RP3 and Template 12 (PO), FP1, RP1. (B) 5-Bond TL-modified template: Template 1 (TL**1**), FP2, RP2. (C) 6-Bond TL-modified templates: Template 2 (TL**2**), FP1, RP1; Template 3 (TL**3**), FP1, RP1 and Template 4 (TL**4**), FP1, RP1. (D) 7-Bond TL-modified templates: Template 5 (TL**5**), FP1, RP1; Template 6 (TL**6**), FP3, RP3 and Template 7 (TL**7**), FP1, RP1.

DNA sequencing

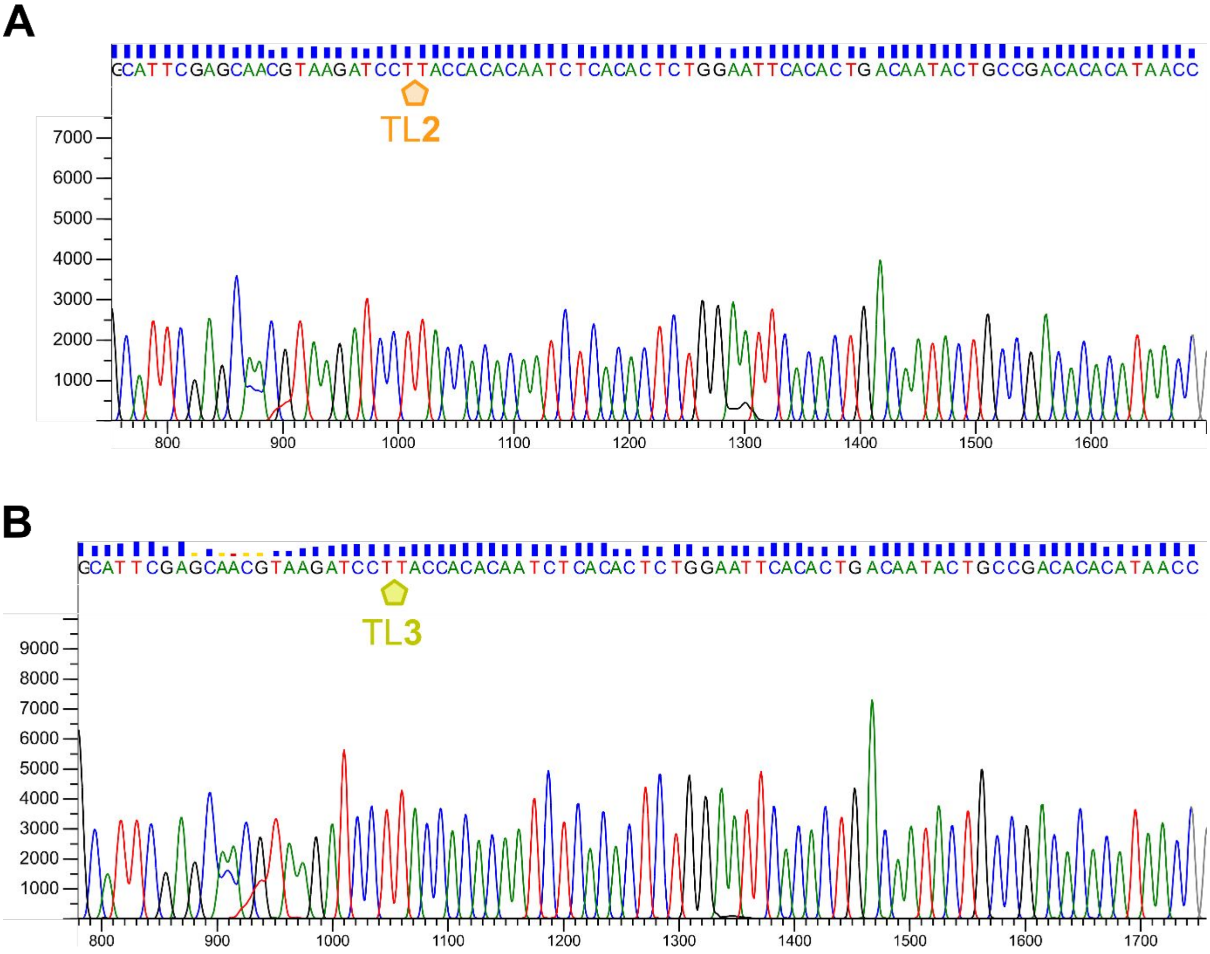

Figure S23. Representative Sanger sequencing results for PCR products of the TL-containing templates using tailed primers (LIC-FP and LIC-RP; **Table S1**) and hot-start Taq polymerase. Amplicons were cloned into a vector and the insertion sites were sequenced by Sanger sequencing. The original positions of the TLs from the corresponding template sequences are indicated as pentagons. Templates used: (A) Template 2 (TL**2**), n = 4. (B) Template 3 (TL**3**), n = 4.

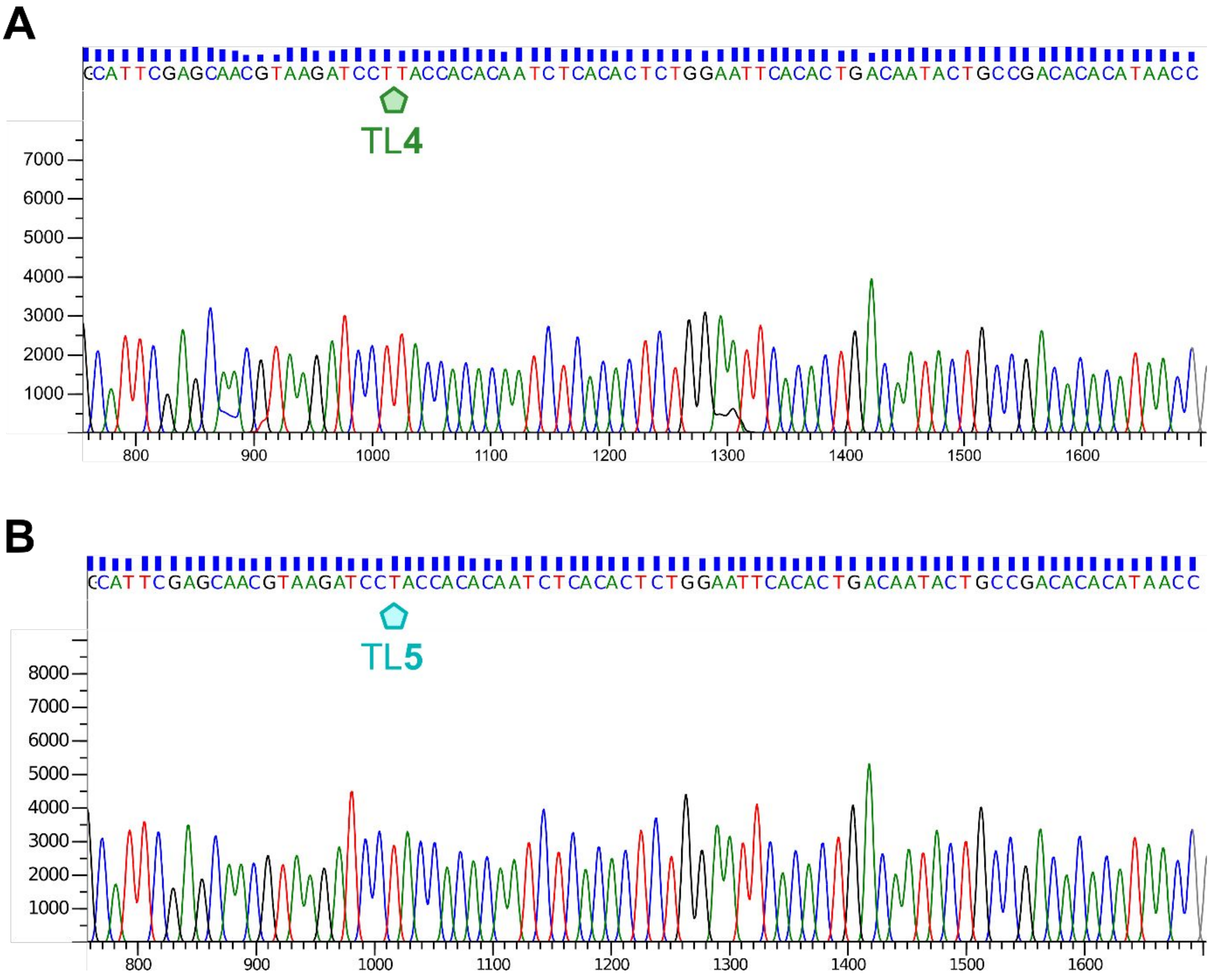

Figure S24. Representative Sanger sequencing results for PCR products using tailed primers (LIC-FP and LIC-RP; **Table S1**) and hot-start Taq polymerase. Amplicons were cloned into a vector and insertion sites were sequenced by Sanger sequencing. The original positions of the TLs from the corresponding template sequences are indicated as pentagons. Templates used: (A) Template 4 (TL**4**), n = 4. (B) Template 5 (TL**5**), n = 5. Note that TL**5** causes a deletion mutation around the triazole for which the original $T^{TL\mathbf{5}}T$ motif has been read as a single T.

Protocols and procedures

UV melting studies

UV DNA melting curves were recorded on a Cary 4000 Scan UV-Visible Spectrophotometer using 3 μM of each ON in a 10 mM phosphate buffer containing 200 mM NaCl at pH 7.0. Samples were annealed by heating to 85 °C (10 °C/min) and then slowly cooling to 20 °C (1 °C/min). Six successive cycles (heating and cooling) were then performed at a gradient of 0.5 °C/min and the change in UV absorbance at 260 nm was recorded. The T_ms for each experiment were calculated from the average of the 1st derivatives of the melting curves using in built software. The T_ms are reported as the average from two independent experiments.

Circular dichroism studies

Circular dichroism (CD) was recorded for duplexes formed from 3 μM ON2, ON5 or ON7 and 3 μM of a complementary DNA or RNA target in 10 mM phosphate buffer with 200 mM NaCl (pH 7.0). CD was performed on a Chirascan Plus spectrometer using a quartz cuvette with a pathlength of 1 mm. Scans were taken at rt from 200–340 nm with 0.5 a step and 1.0 s time point intervals. The average of four scans was taken and smoothed to 20 points using a third order polynomial (Savitzky-Golay, Origin 2017). The spectra were then baseline corrected to the θ-value at 340 nm.

Linear copying assay

Primer extension reactions were performed using the large fragment of DNA polymerase I (Klenow fragment) (NEB, cat. no. M0210S) or hot-start Taq polymerase (NEB, cat. no. M0495S) using a Bio-Rad T100 PCR thermal cycler. Used FL-primers and templates are listed in **Table S1**. For Klenow fragment, a master-mix composed of NEB buffer 2 (10×, 1 μL), dNTPs (10 mM, 0.2 μL), polymerase (5 U/μL, 0.2 μL) and water (3.6 μL) was prepared. This mix (5 μL) was then added to a solution of FL-primer (10 μM, 1.5 μL), template (10 μM, 2.3 μL) and water (1.2 μL). Practically, ON solutions were mixed with the master mix and immediately incubated on a pre-warmed thermocycler at 37 °C for 7.5 min. Reactions were quenched at 37 °C using a stop solution [1 mM EDTA-Na_2/formamide, 1/1 (v/v), 20 μL] before storage at –20 °C in the dark. For hot-start Taq polymerase, a master-mix composed of standard Taq buffer (10×, 1 μL), dNTPs (10 mM, 0.2 μL), polymerase (5 U/μL, 0.5 μL) and water (3.3 μL) were prepared. This mix (5 μL) was then added to a solution of FL-primer (10 μM, 1.5 μL), template (10 μM, 2.3 μL) and water (1.2 μL). Practically, ON solutions were mixed with the master mix and immediately heat activated at 95 °C for 2 min before primer extension at 60 °C for 160 min. Reactions were quenched using a stop solution [1 mM EDTA-Na_2/formamide, 1/1 (v/v), 20 μL] before storage at –20 °C in the dark.

The samples were separated by 8% denaturing PAGE (400 V) and imaged using a G:Box (*Syngene*) with an excitation wavelength of 460 nm and an emission filter of 516–600 nm for detection of the FL-labelled ONs.

Linear copying for mass spectrometry

Primer extension reactions were performed as described in the "*Linear copying assay*" section but scaled up four times. After the specified extension times, Klenow fragment reactions were heated to 75 °C for 20 min to inactivate the polymerase. Hot-start Taq polymerase reactions were inactivated by addition of EDTA-Na_2 (160 mM, 3.33 μL). Each reaction was then mixed with PhOH/$CHCl_3$ (40 μL, ThermoFisher Scientific, cat. no. 15593031), vortexed for 30 s and

centrifuged (1000 rpm, 5 min). The aqueous layer was then transferred to a new tube, mixed with NaOAc (4 µL, 3 M, pH 5.2) and EtOH (132 µL), before incubating at –80 °C overnight. The samples were then centrifuged (13,000 rpm, 20 min), the supernatant removed and the pellet re-dissolved in water (10 µL). These reaction mixtures were analyzed by Ultra Performance Liquid Chromatography-Mass Spectrometry (UPLC-MS) as described in section '*Mass spectrometry*'.

qPCR kinetics

qPCR reactions were performed using hot-start Taq polymerase (NEB, cat. no. M0495S) on a Bio-Rad CFX96 real-time PCR detection system. Template and primers can be found in **Table S1**. Master-mixes composed of standard Taq buffer (10×, 2 µL), EvaGreen (20×, 1 µL), dNTPs (10 mM, 0.4 µL), polymerase (5 U/µL, 0.1 µL) and water (13.5 µL) were prepared. This mix (17 µL) was then added to a solution of FP (10 µM, 1 µL), RP (10 µM, 1 µL) and template (18.7 pM, 1 µL). Samples for all qPCR extension times were prepared together and stored at 4 °C before sequential use. PCR thermal cycling conditions consisted of thermal activation (120 s, 95 °C), and 31 cycles of denaturation (15 s, 95 °C) and annealing/extension (8, 15, 30, 45, 60, 120 or 240 s, 60 °C), with emission recorded at the end of each extension step. Samples were excited at 450–490 nm and emission monitored at 510–530 nm. For melt curves analysis, samples were heated from 60 to 90 °C after qPCR with emission recorded every 0.5 °C using a ramp rate of 6 °C/min. Single products were confirmed by single peaks in dF/dT vs. T plots. Threshold cycles were determined at a fluorescence set to F = 300 (a.u.).

DNA sequencing

TL-containing templates [Template 2 (TL**2**), Template 3 (TL**3**), Template 4 (TL**4**) and Template 5 (TL**5**)] were amplified using hot-start Taq polymerase (NEB, cat. no. M0495S) and tailed primers (LIC-FP, LIC-RP; **Table S1**) for cloning using an aLICator LIC cloning and expression kit (Thermo Fisher, cat. no. K1241). Master-mixes composed of standard Taq buffer (10×, 2 µL), dNTPs (10 mM, 0.4 µL), polymerase (5 U/µL, 0.1 µL) and water (14.5 µL) were prepared. This mix (17 µL) was then added to a solution of LIC-FP (10 µM, 1 µL), LIC-RP (10 µM, 1 µL) and template (18.7 pM, 1 µL). PCR thermal cycling conditions consisted of thermal activation (120 s, 95 °C), and 31 cycles of denaturation (15 s, 95 °C) and annealing/extension (45 s, 60 °C) and a final extension time of 7 min at 60 °C. Samples were then purified using a QIAquick PCR purification kit (QIAGEN, cat. no. 28104) and successful amplification was confirmed by 2% agarose gel electrophoresis with 5 µL of the purified samples. The purified PCR products were annealed to a pLATE11 vector according to the aLICator LIC cloning and expression protocol. The annealing mixture (2 µL) was used for transformation of 20 µL of NEB 5-alpha competent *E. coli* cells (C2987I) according to the vendor's High Efficiency Transformation Protocol. Cells were spread onto an LB agar selection plate supplemented with ampicillin (100 µg/mL). Following incubation at 37 °C for 16 h, colonies were picked and grown in a small-scale culture (10 mL) containing LB media and 100 µg/mL ampicillin at 37 °C for 16 h with 250 rpm. The plasmid DNA was extracted from the bacterial cells using the QIAprep Spin Miniprep kit according to the manufacturer's protocol (QIAGEN, cat. no. 27104). The plasmid was eluted with 50 µL water and the concentration was measured using a NanoDrop™ 2000/2000c spectrophotometer (Thermo Fisher Scientific). Plasmids were sent for Sanger sequencing with a suitable primer (Seq-FP, **Table S1**) according to the sample preparation requirements.

Mass spectrometry

ON MS was recorded on a UPLC-MS Waters XEVO G2-QTOF (ESI$^-$) using an ACQUITY UPLC ON BEH C18 column, 130 Å (1.7 µm, 2.1 mm × 50 mm). A gradient of CH_3OH in Et_3N and hexafluoroisopropanol (HFIP) was used (buffer A, 8.60 mM Et_3N, 200 mM HFIP in 5% CH_3OH/H_2O (v/v); buffer B, 20% buffer A in CH_3OH). Buffer B was increased from 0–70% over 8 min, at a flow rate of 0.2 mL·min^{-1}. Raw data were processed and deconvoluted using the Waters MassLynx v4.1 software package.

Synthesis and purification of oligonucleotides

Synthesis of DNA oligonucleotides

Standard DNA phosphoramidites, solid supports and reagents were purchased from Link Technologies and Applied Biosystems. 5′-Fluorescein-CE phosphoramidite (6-FAM) was purchased from Link Technologies (cat. No. 2134). Automated solid phase synthesis of ONs (trityl off) was performed on an Applied Biosystems 394 synthesizer. Synthesis was performed on a 1.0 µmol scale involving cycles of acid-catalyzed detritylation, coupling, capping, and iodine oxidation. Standard DNA phosphoramidites were coupled for 60 s and chemically modified phosphoramidites for 10 min. Coupling efficiencies and overall synthesis yields were determined by the inbuilt automated trityl cation conductivity monitoring facility. The ONs were then cleaved from the solid support and protecting groups of the nucleobase and backbone were removed by exposure to concentrated aq NH_3 for 60 min at room temperature followed by heating in a sealed glass vial at 55 °C for 5 h.

Synthesis of RNA oligonucleotides

2′-*O*-*tert*-Butyldimethylsilyl (2′-TBS) protected RNA phosphoramidite monomers with *tert*-butylphenoxyacetyl protection of the A, G and C nucleobases were used to assemble RNA ONs. Benzylthiotetrazole (BTT) was used as the coupling agent, *tert*-butylphenoxyacetic anhydride as the capping agent and 0.1 M iodine as the oxidizing agent (*Sigma-Aldrich*). Coupling time of 10 min was used and coupling efficiencies of >97% were obtained. Cleavage of ONs from the solid support and protecting groups of the nucleobase and backbone were removed by exposure to concentrated aq NH_3/EtOH [3/1 (v/v), 4 mL] for 2 h at room temperature followed by heating in a sealed glass vial for 2 h at 55°C.

Removal of 2′-TBS protection of RNA oligonucleotides

After cleavage from the solid support and removal of the protecting groups from the nucleobases and phosphodiesters in concentrated aq NH_3/EtOH [3/1 (v/v), 4 mL] as described above, ONs were concentrated to a small volume under reduced pressure, transferred to 15 mL plastic tubes and freeze dried (lyophilized). The residue was dissolved in dimethylsulfoxide (DMSO) (300 µL) and $Et_3N·3HF$ (300 µL) was added after which the reaction mixtures were kept at 65 °C for 2.5 h. NaOAc (3 M, 50 µL) and *n*-BuOH (3 mL) were added with vortexing and the samples were kept at −80 °C for 30 min before centrifugation at 13,000 rpm at 4 °C for 10 min. The supernatant was decanted and the precipitate was washed twice with 70% EtOH (0.75 mL) then dried *in vacuo*.

HPLC purification of oligonucleotides (DNA or RNA)

The fully deprotected ONs were purified by RP-HPLC on a Gilson system using a Luna 10 µm C8(2) 100 Å pore Phenomenex column (250 × 10 mm) with a gradient of MeCN in aq

triethylammonium bicarbonate (TEAB) (buffer A, 0.1 M TEAB, pH 7.5; buffer B, 50% buffer A in MeCN). Buffer B was increased 20–50% over 20 min at a flow rate of 4 $mL \cdot min^{-1}$ and elution was monitored by UV absorption between 260–295 nm.

Oligonucleotide purification by gel electrophoresis

The samples were separated by 8% denaturing PAGE (400 V, ca. 1 nmol of ON per lane) and the gel was visualized under UV. Bands of interest were excised, crushed and incubated in a ThermoMixer (*Eppendorf*) at 37 °C, 950 rpm in water (ca. 1 mL per band) for 16 h. The polyacrylamide was then filtered off and the filtrate was concentrated under reduced pressure. After desalting using illustra™ NAP-10 and NAP-25 columns (GE Healthcare) consecutively, extracted ONs were analyzed by MS as described in the above section.

Synthetic Procedures

General

All reagents for synthesis were purchased from Sigma Aldrich and used without further purification. Anhydrous solvents (CH_2Cl_2, THF, 1,4-dioxane) were obtained using an MBraun SPS Bench Top solvent purification system (SPS). All air/moisture sensitive reactions were carried out under inert atmosphere (argon) in oven-dried glassware. Solvents used for phosphitylation reactions and purification were also degassed by bubbling the solvents with argon for 20 min. Reactions were monitored by thin-layer chromatography using Merck Kieselgel 60 F24 silica gel plates (0.22 mm thickness, aluminum backed), visualized by UV irradiation at 254/265 nm and by staining with *p*-anisaldehyde followed by gentle heating. Column chromatography was carried out under pressure (Biotage® SP4) using Biotage® Sfär columns. ^{1}H NMR (400 MHz, 500 MHz or 600 MHz), ^{13}C NMR (101 MHz, 126 MHz or 151 MHz) and ^{31}P NMR (162 MHz) spectra were measured on a Bruker AVIIIHD 400, a Bruker AVII 500 (with a ^{13}C cryoprobe) or a Bruker AVIII 600 (with a prodigy N_2 broadband cryoprobe) spectrometer. ^{1}H and ^{13}C NMR spectra were referenced to the appropriate deuterated solvent signal and chemical shifts are given in ppm and all coupling constants (*J*) are quoted in Hertz (Hz). Assignment of the compounds was aided by correlation spectroscopy (COSY; ^{1}H - ^{1}H), heteronuclear single quantum correlation (HSQC; ^{1}H - ^{13}C) and HMBC (^{1}H - ^{13}C) experiments when feasible. High-resolution mass spectra (HRMS) were recorded by the Departmental Mass Spectrometry Service, University of Oxford on a Thermo Scientific Exactive Mass Spectrometer (using a Waters Equity autosampler and pump) for electrospray ionization (ESI) and an Agilent 7200 Accurate Mass QTOF GCMS (using a SIM Direct Insertion Probe) for electron ionization (EI) and chemical ionization (CI). High-resolution values are calculated to 4 decimal places from the molecular formula, and all values are within a tolerance of 5 ppm.

5′-*O*-Propargyl-3′-*O*-(*tert*-butyldiphenylsilyl)-thymidine (**2**)

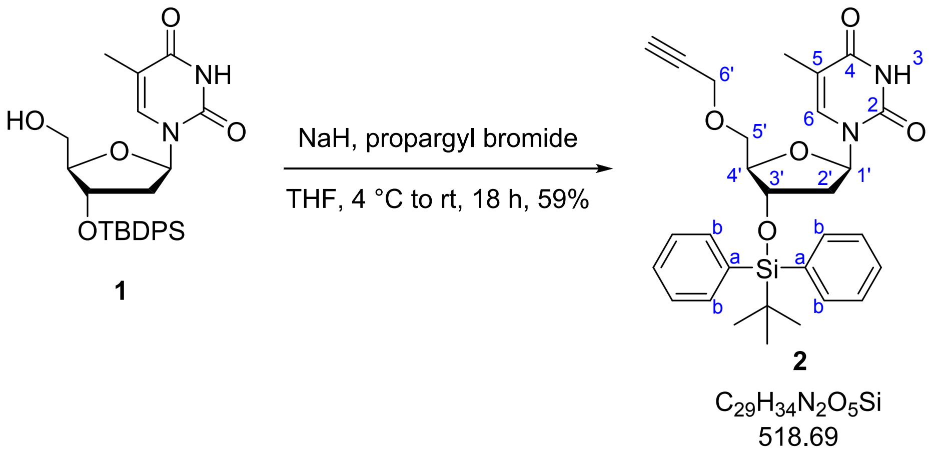

A solution of alcohol **1**[3] (5.31 g, 11.0 mmol) in anhyd THF (35 mL) was stirred at 4 °C. A 60% dispersion of NaH in mineral oil (1.33 g, 33.1 mmol) was added and the mixture was stirred at 4 °C for 20 min. Propargyl bromide (0.83 mL, 11.0 mmol) was added at 4 °C over 1 h. The mixture was allowed to warm up to rt and stirred for 18 h. The reaction was neutralized to pH 7 with 1 N aq AcOH and diluted with Et_2O (75 mL). The organic layer was washed with brine (2 × 75 mL), dried over Na_2SO_4, filtered and evaporated. The crude was purified by column chromatography (petroleum ether 40–60/EtOAc, 0–50%) to give alkyne **2** as a white solid (3.35 g, 6.46 mmol, 59%).

R

$\boldsymbol{R_f}$ 0.69 (petroleum ether 40–60/EtOAc, 1/1);

^{1}H NMR (500 MHz, $CDCl_3$) δ 8.52 (s, 1H, NH), 7.68 – 7.60 (m, 4H, H-Ar), 7.52 (d, *J* = 1.3 Hz, 1H, H6), 7.49 – 7.43 (m, 2H, H-Ar), 7.43 – 7.36 (m, 4H, H-Ar), 6.48 (dd, *J* = 8.1, 5.8 Hz, 1H, H1′), 4.43 (dt, *J* = 5.8, 2.3 Hz, 1H, H3′), 4.06 – 3.98 (m, 2H, H4′ *and* H6′), 3.95 (dd, *J* = 15.7, 2.4 Hz, 1H, H6″), 3.55 (dd, *J* = 10.4, 2.4 Hz, 1H, H5′), 3.03 (dd, *J* = 10.4, 2.4 Hz, 1H, H5″), 2.36 (t, *J* = 2.4 Hz, 1H, C≡CH), 2.32 (ddd, *J* = 13.5, 5.8, 2.3 Hz, 1H, H2′), 1.94 (ddd, *J* = 13.5, 8.1, 5.8 Hz, 1H, H2″), 1.88 (d, *J* = 1.3 Hz, 3H, CH_3-*thymine*), 1.08 (s, 9H, 3 × CH_3-*tert butyl*);

^{13}C NMR (126 MHz, $CDCl_3$) δ 163.8 (C4), 150.4 (C2), 136.1 (C6), 135.9 (^{b}CH-Ar), 135.9 (^{b}CH-Ar), 133.4 (^{a}C-Ar), 133.2 (^{a}C-Ar), 130.2 (CH-Ar), 128.0 (CH-Ar), 111.0 (C5), 86.6 (C4′), 85.2 (C1′), 78.9 (<u>C</u>≡CH), 75.3 (C≡<u>C</u>H), 74.0 (C3′), 69.5 (C5′), 58.5 (C6′), 41.2 (C2′), 27.0 (C(<u>C</u>H_3)$_3$), 19.1 (<u>C</u>(CH_3)$_3$), 12.7 (CH_3-*thymine*); ^{a,b}Note that the marked carbons can resolve in distinct peaks due to different environments.

HRMS–ESI (*m/z*): $[M + H]^+$ calcd for $C_{29}H_{35}N_2O_5Si$, 519.2310; found, 519.2307.

5′-*O*-(4,4′-Dimethoxytrityl)-thymidine-1,5-triazole-3′-*O*-(*tert*-butyldiphenylsilyl)-thymidine dimer (**4**)

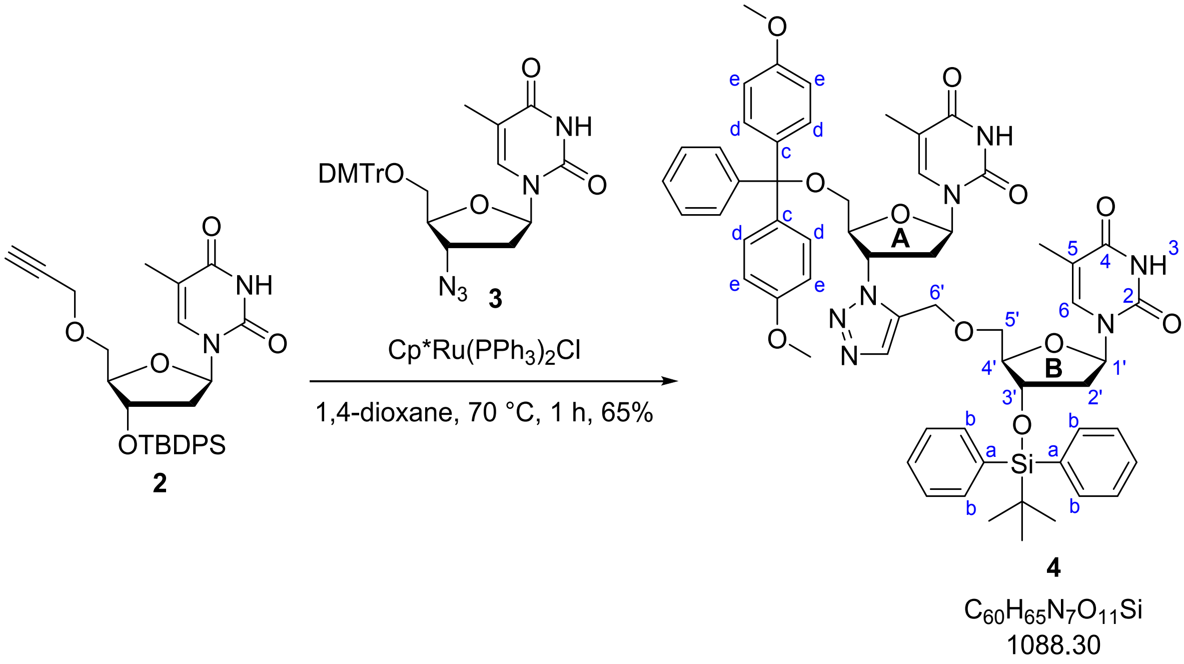

Azide **3** (1.27 g, 2.23 mmol) and alkyne **2** (1.85 g, 3.57 mmol) were co-evaporated with anhyd 1,4-dioxane (2 × 10 mL) and dissolved in anhyd 1,4-dioxane (17 mL). Cp*RuCl$(PPh_3)_2$ (355 mg, 0.45 mmol) was added and the reaction was stirred at 70 °C for 1 h. The solvent was evaporated under reduced pressure and the residue re-dissolved in EtOAc (150 mL) and consecutively washed with sat. aq EDTA-Na_2 (150 mL), sat. aq $NaHCO_3$ (150 mL) and brine (150 mL). The separated organic layer was dried over Na_2SO_4, filtered and evaporated. Column chromatography of the crude (petroleum ether 40–60/EtOAc, 30–100%) gave dimer **4** (1.59 g, 1.46 mmol, 65%).

***R*$_f$** 0.45 (EtOAc);

^{1}H NMR (400 MHz, $CDCl_3$) δ 8.98 (2 × s, 2H, 2 × NH), 7.65 – 7.52 (m, 5H, H6 *and* 5 × H-Ar), 7.46 (s, 1H, H-*triazole*), 7.44 – 7.39 (m, 2H, H-Ar), 7.38 – 7.29 (m, 6H, H-Ar), 7.28 – 7.24 (m, 2H, H-Ar *and* $CDCl_3$), 7.24 – 7.17 (m, 5H, H-Ar), 6.88 (d, *J* = 1.3 Hz, 1H, H6), 6.85 – 6.74 (m, 4H, H-Ar), 6.49 (t, *J* = 6.5 Hz, 1H, H1′A), 6.24 (dd, *J* = 7.4, 6.3 Hz, 1H, H1′B), 5.16 (dt, *J* = 8.7, 4.8 Hz, 1H, H3′A), 4.38 (ddd, *J* = 4.8, 3.6, 3.0 Hz, 1H, H4′A), 4.34 – 4.23 (m, 2H, H6′ *and* H6″), 4.20 (dt, *J* = 6.8, 3.4 Hz, 1H, H3′B), 3.96 – 3.90 (m, 1H, H4′B), 3.76 (2 × s, 6H, 2 × OCH_3), 3.47 (dd, *J* = 10.8, 3.6 Hz, 1H, H5′A), 3.26 (dd, *J* = 10.8, 3.0 Hz, 1H, H5″A), 3.16 (dd, *J* = 10.5, 2.9 Hz, 1H, H5′B), 3.06 – 2.93 (m, 2H, H2′A *and* H5″B), 2.64 (ddd, *J* = 13.7, 8.7, 6.5 Hz, 1H, H2″A), 2.28 (ddd, *J* = 13.4, 6.3, 3.4 Hz, 1H, H2′B), 1.93 – 1.77 (m, 1H, H2″B), 1.66 (d, *J* = 1.3 Hz, 3H, CH_3-*thymine*), 1.60 (d, *J* = 1.3 Hz, 3H, CH_3-*thymine*), 1.04 (s, 9H, CH_3-*tert butyl*);

^{13}C NMR (126 MHz, $CDCl_3$) δ 163.9 (C4), 163.6 (C4), 158.9 (C-Ar), 150.3 (C2), 150.2 (C2), 144.3 (C-Ar), 136.2 (C6), 135.8 (^{b}CH-Ar), 135.7 (^{b}CH-Ar), 135.4 (C6), 135.2 (^{c}C-Ar), 135.1 (^{c}C-Ar), 134.1 (H<u>C</u>=C-*triazole*), 133.1 (^{a}C-Ar *or* HC=<u>C</u>-*triazole*), 133.0 (^{a}C-Ar *or* HC=<u>C</u>-*triazole*), 132.9 (^{a}C-Ar *or* HC=<u>C</u>-*triazole*), 130.3 (CH-Ar), 130.0 (^{d}CH-Ar), 130.0 (^{d}CH-Ar), 128.2 (CH-Ar), 128.1 (CH-Ar), 128.0 (CH-Ar), 127.4 (CH-Ar), 113.5 (^{e}CH-Ar), 113.4 (^{e}CH-

Ar), 111.4 (C5), 111.3 (C5), 87.2 (C_{quat}-*DMTr*), 86.6 (C1′A), 85.5 (C4′B), 85.2 (C1′B), 83.8 (C4′A), 73.1 (C3′B), 70.1 (C5′B), 63.2 (C5′A), 61.0 (C6′), 58.4 (C3′A), 55.4 (OCH_3), 40.3 (C2′B), 38.6 (C2′A), 26.9 (C($\underline{C}H_3$)$_3$), 19.1 ($\underline{C}$(CH_3)$_3$), 12.6 (CH_3-*thymine*), 12.1 (CH_3-*thymine*); [a,b,c,d,e]Note that the marked carbons can resolve in distinct peaks due to different environments.

HRMS–ESI (*m/z*): $[M + Na]^+$ calcd for $C_{60}H_{65}N_7O_{11}SiNa$, 1110.4404; found, 1110.4397.

5′-*O*-(4,4′-Dimethoxytrityl)-thymidine-1,5-triazole-thymidine dimer (**5**)

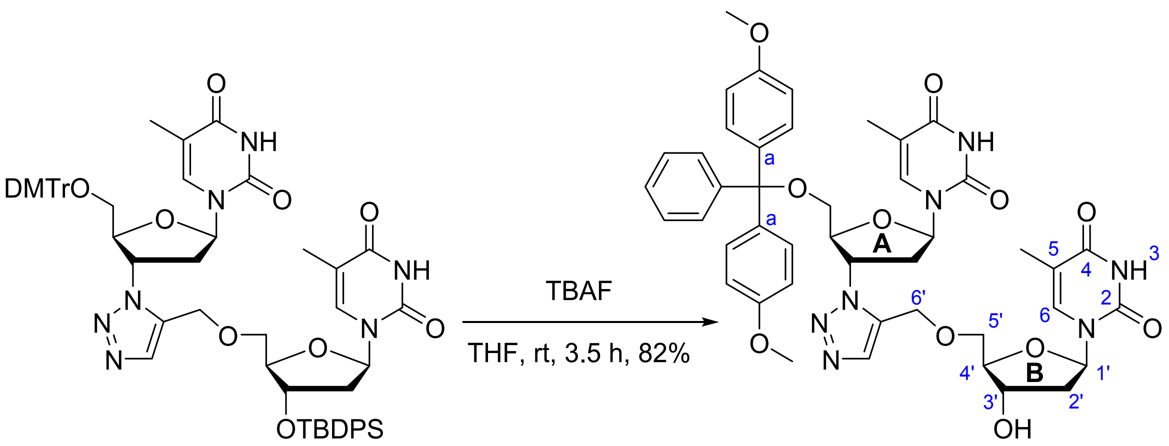

4 **5**

$C_{44}H_{47}N_7O_{11}$
849.90

Tetrabutylammonium fluoride (TBAF, 1 M in THF, 2.05 mL, 2.05 mmol) was added to a solution of dimer **4** (1.49 g, 1.37 mmol) in THF (12 mL) and stirred at rt for 3.5 h. The solvent was evaporated and the remaining solid submitted to column chromatography (EtOAc/propan-2-ol, 0–20%) to give dimer **5** as a white foam (0.95 g, 1.12 mmol, 82%).

***R*f** 0.51 (EtOAc/propan-2-ol, 4/1);

^{1}H NMR (500 MHz, $CDCl_3$) δ 10.54 (bs, 1H, NH), 9.96 (s, 1H, NH), 7.60 (s, 1H, H-*triazole*), 7.44 – 7.40 (m, 1H, H6), 7.37 – 7.32 (m, 2H, H-Ar), 7.29 – 7.16 (m, 8H, H6 *and* 7 × H-Ar), 6.84 – 6.77 (m, 4H, H-Ar), 6.15 (t, *J* = 5.9 Hz, 1H, H1′B), 6.08 (dd, *J* = 7.7, 4.4 Hz, 1H, H1′A), 5.57 (q, *J* = 7.8 Hz, 1H, H3′A), 4.58 – 4.51 (m, 2H, H3′B *and* H4′A), 4.48 (bs, 1H, OH), 4.44 (bs, 2H, H6′ *and* H6″), 4.03 – 3.96 (m, 1H, H4′B), 3.75 (2 × s, 6H, 2 × OCH_3), 3.73 – 3.63 (m, 2H, H5′B and H5″B), 3.54 (dd, *J* = 10.8, 3.8 Hz, 1H, H5′A), 3.23 – 3.11 (m, 2H, H2′A *and* H5″A), 2.97 – 2.87 (m, 1H, H2″A), 2.35 (dt, *J* = 13.7, 5.9 Hz, 1H, H2′B), 2.11 – 2.03 (m, 1H, H2″B), 1.71 (d, *J* = 1.1 Hz, 3H, CH_3-*thymine*), 1.49 (d, *J* = 1.2 Hz, 3H, CH_3-*thymine*);

^{13}C NMR (126 MHz, $CDCl_3$) δ 165.0 (C4), 164.2 (C4), 158.8 (C-Ar), 150.9 (C2), 150.6 (C2), 144.2 (C-Ar), 138.5 (C6), 135.7 (C6), 135.4 ([a]C-Ar), 135.2 ([a]C-Ar), 133.8 (H$\underline{C}$=C-*triazole or* HC=$\underline{C}$-*triazole*), 133.7 (H$\underline{C}$=C-*triazole or* HC=$\underline{C}$-*triazole*), 130.0 (CH-Ar), 128.1 (CH-Ar), 128.1 (CH-Ar), 127.3 (CH-Ar), 113.4 (CH-Ar), 111.2 (C5), 111.0 (C5), 88.6 (C1′A), 86.9 (C_{quat}-*DMTr*), 85.2 (C1′B), 84.9 (C4′B), 83.6 (H4′A), 70.6 (H3′B *or* C5′B), 70.5 (H3′B *or* C5′B), 62.3 (C5′A), 60.8 (C6′), 57.7 (C3′A), 55.4 (OCH_3), 40.9 (C2′B), 38.3 (C2′A), 12.3 (CH_3-*thymine*), 12.2 (CH_3-*thymine*); [a]Note that the marked carbons can resolve in distinct peaks due to different environments.

HRMS–ESI (*m/z*): $[M + Na]^+$ calcd for $C_{44}H_{47}N_7O_{11}Na$, 872.3226; found, 872.3234.

5′-*O*-(4,4′-Dimethoxytrityl)-thymidine-1,5-triazole-thymidine dimer phosphoramidite (**6**)

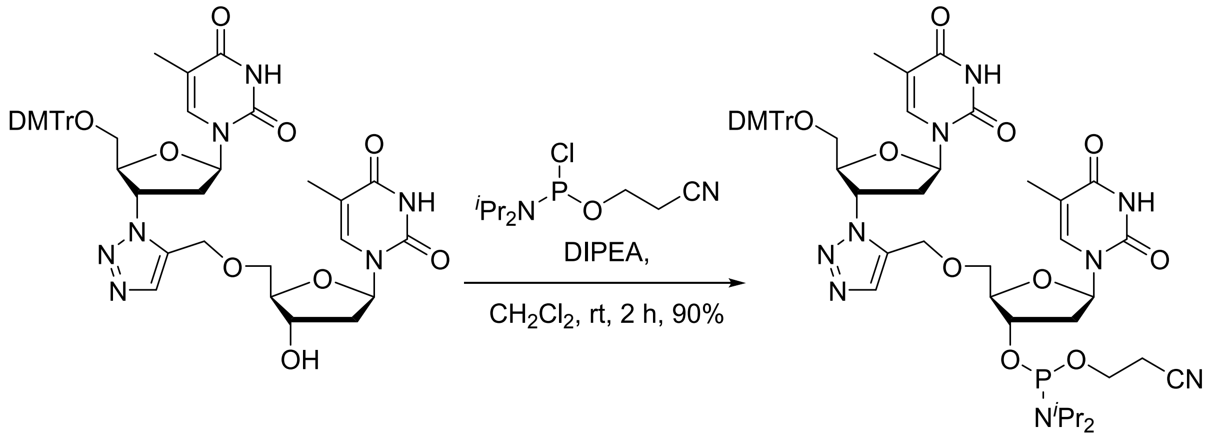

5

6

$C_{53}H_{64}N_9O_{12}P$
1050.12

Degassed *N*,*N*-diisopropylethylamine (DIPEA, 370 µL, 2.13 mmol) and chloro(diisopropylamino)-β-cyanoethoxyphosphine (240 µL, 1.07 mmol) were added to a solution of dimer **5** (400 mg, 0.47 mmol) in degassed, anhyd CH_2Cl_2 (3 mL) and the reaction mixture was stirred at rt for 2 h. The solution was diluted with degassed CH_2Cl_2 (10 mL) and then washed with sat. aq KCl (10 mL). The aq layer was extracted with degassed CH_2Cl_2 (2 × 10 mL) and the combined organic layers were dried over Na_2SO_4, filtered and the solvents evaporated under reduced pressure. Purification by column chromatography (EtOAc) gave phosphoramidite **6** as a white foam (438 mg, 0.42 mmol, 90%).

***R*f** 0.40 (EtOAc);

^{31}P NMR (162 MHz, $CDCl_3$) δ 148.5, 148.1;

HRMS–ESI (*m/z*): $[M + Na]^+$ calcd for $C_{53}H_{64}N_9O_{12}PNa$, 1072.4304; found, 1072.4295.

5′-*O*-(4,4′-Dimethoxytrityl)-thymidine-1,4-triazole-3′-*O*-(*tert*-butyldiphenylsilyl)-thymidine dimer (**7**)

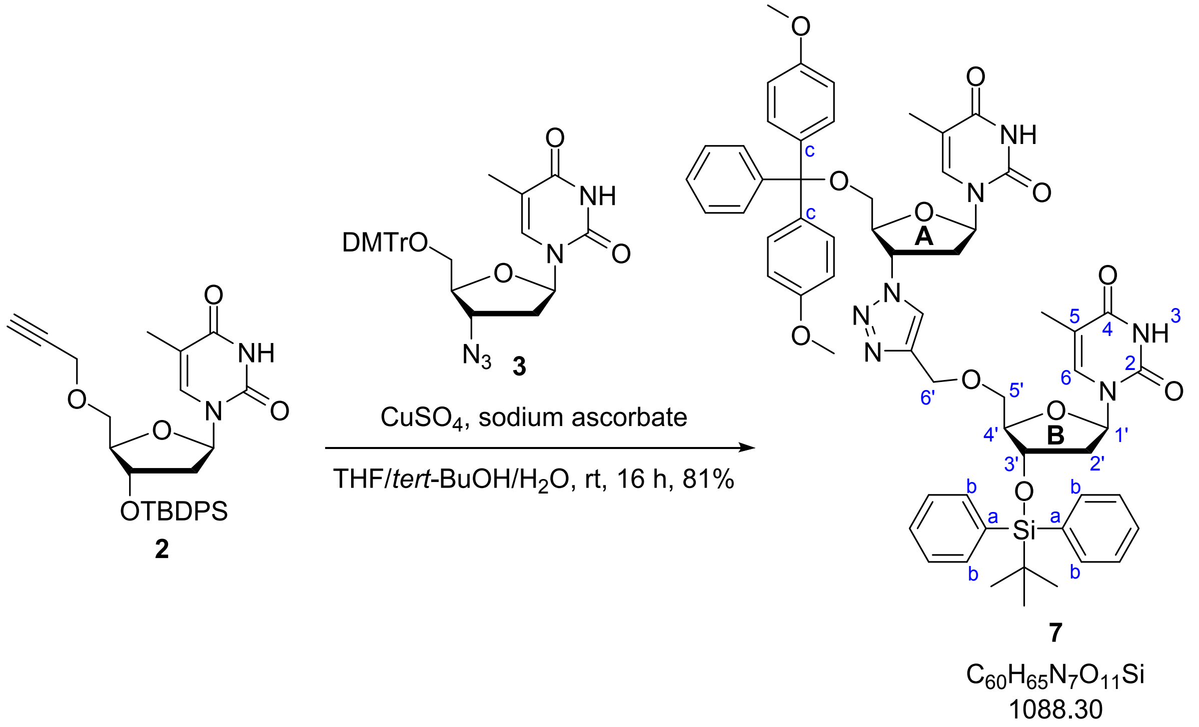

Azide **3** (0.74 g, 1.29 mmol) and alkyne **2** (0.61 g, 1.18 mmol) were dissolved in a solvent mixture of THF/*tert*-BuOH/H_2O [3/1/1 (v/v/v), 10 mL], 2 drops of pyridine were added, and the mixture was stirred at rt. A solution of $CuSO_4 \cdot 5\,H_2O$ (182 mg, 0.73 mmol) in H_2O (2.42 mL) and 1 M aq sodium ascorbate (2.73 mL) were added, the reaction mixture was then degassed with argon, and stirred at rt for 16 h. The reaction was diluted with EtOAc (30 mL) and washed with water, (30 mL), aq EDTA-Na_2 [5% (w/v), 3 × 30 mL) and brine (30 mL). The separated organic layer was dried over Na_2SO_4, filtered and evaporated. The crude product was purified by column chromatography (CH_2Cl_2/CH_3OH, 0–5%, + 1% pyridine) to give dimer **7** as a white solid (1.04 g, 0.96 mmol, 81%).

***R*f** 0.29 (CH_2Cl_2/CH_3OH, 19/1);

^{1}H NMR (600 MHz, DMSO-*d*$_6$) δ 11.40 (s, 1H, NH), 11.29 (s, 1H, NH), 8.18 (s, 1H, H-*triazole*), 7.66 (d, *J* = 1.2 Hz, 1H, H6), 7.63 – 7.56 (m, 4H, H-Ar), 7.49 – 7.38 (m, 7H, H6 *and* 6 × H-Ar), 7.37 – 7.32 (m, 2H, H-Ar), 7.29 – 7.17 (m, 7H, H-Ar), 6.84 (dd, *J* = 8.9, 3.4 Hz, 4H, H-Ar), 6.42 (t, *J* = 6.5 Hz, 1H, H1′A), 6.31 (dd, *J* = 8.2, 5.9 Hz, 1H, H1′B), 5.54 (dt, *J* = 8.7, 6.7 Hz, 1H, H3′A), 4.50 – 4.38 (m, 3H, H6′, H6″ *and* H3′B), 4.30 (dt, *J* = 6.7, 4.1 Hz, 1H, H4′A), 4.02 (q, *J* = 3.3 Hz, 1H, H4′B), 3.71 (2 × s, 6H, 2 × OCH_3), 3.49 (dd, *J* = 10.7, 3.3 Hz, 1H, H5′B), 3.34 – 3.23 (m, 3H, H5′A, H5″A *and* H5″B), 2.81 – 2.70 (m, 2H, H2′A, H2″A), 2.16 – 2.09 (m, 1H, H2′B), 2.07 – 2.01 (m, 1H, H2″B), 1.61 (d, *J* = 1.2 Hz, 3H, CH_3-*thymine*), 1.59 (d, *J* = 1.2 Hz, 3H, CH_3-*thymine*), 1.03 (s, 9H, CH_3-*tert butyl*);

^{13}C NMR (151 MHz DMSO-*d*$_6$) δ 163.7 (C4), 163.6 (C4), 158.1 (C-Ar), 150.4 (C2), 150.4 (C2), 144.6 (C-Ar), 143.7 (HC=<u>C</u>-*triazole*), 136.3 (C6), 135.6 (C6), 135.3 (^{b}CH-Ar), 135.2 (^{b}CH-Ar), 135.2 (^{c}C-Ar), 135.1 (^{c}C-Ar), 132.7 (^{a}C-Ar), 132.7 (^{a}C-Ar), 130.1 (CH-Ar), 129.7 (CH-Ar), 127.9 (CH-Ar), 127.8 (CH-Ar), 127.6 (CH-Ar), 126.7 (CH-Ar), 123.2 (H<u>C</u>=C-*triazole*), 113.2 (CH-Ar), 109.8 (C5), 109.7 (C5), 86.0 (C_{quat}-*DMTr*), 85.4 (C4′B), 84.0 (C1′A),

83.9 (C1′B), 82.3 (C4′A), 73.8 (C3′B), 69.6 (C5′B), 63.8 (C6′), 63.0 (C5′A), 59.3 (C3′A), 55.0 (OCH_3), 55.0 (OCH_3), 39.4 (C2′B *under DMSO-d_6 peaks*), 37.1 (C2′A), 26.6 (C(<u>C</u>H_3)$_3$), 18.6 (<u>C</u>(CH_3)$_3$), 11.9 (CH_3-*thymine*), 11.8 (CH_3-*thymine*); [a,b,c]Note that the marked carbons can resolve in distinct peaks due to different environments.

HRMS–ESI (*m/z*): $[M + Na]^+$ calcd for $C_{60}H_{65}N_7O_{11}SiNa$, 1110.4404; found, 1110.4390.

5′-*O*-(4,4′-Dimethoxytrityl)-thymidine-1,4-triazole-thymidine dimer (**8**)

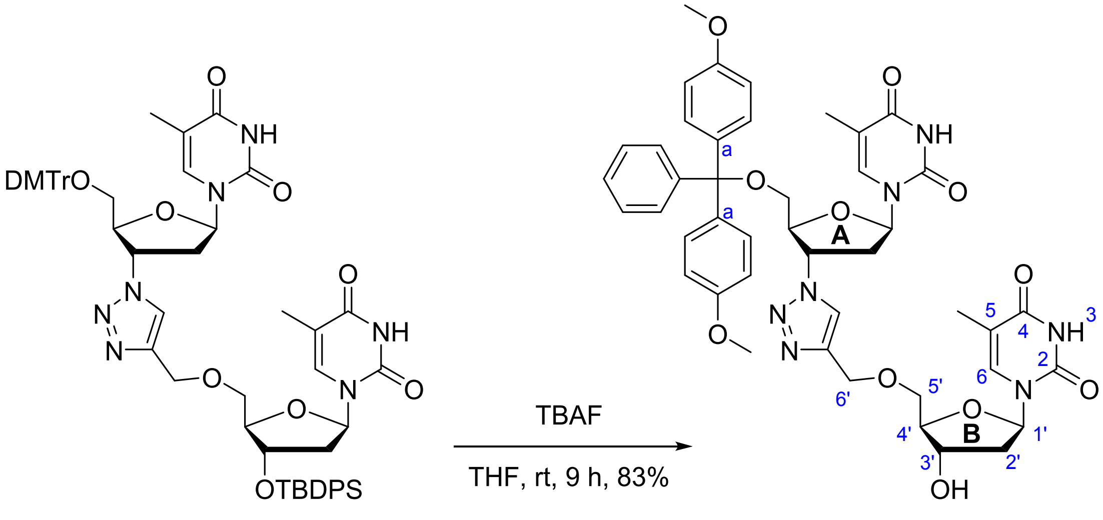

7 **8**

$C_{44}H_{47}N_7O_{11}$
849.90

TBAF (1 M in THF, 0.69 mL, 0.69 mmol) was added to a solution of dimer **7** (500 mg, 0.46 mmol) in THF (4 mL) and stirred at rt for 9 h. The solvent was evaporated and the remaining solid submitted to column chromatography (CH_2Cl_2/CH_3OH, 0–10%) to give dimer **8** as a white foam (327 mg, 0.38 mmol, 83%).

***R*$_f$** 0.24 (CH_2Cl_2/CH_3OH, 9/1);

^{1}H NMR (500 MHz, DMSO-*d*$_6$) δ 11.40 (s, 1H, NH), 11.27 (s, 1H, NH), 8.30 (s, 1H, H-*triazole*), 7.65 (d, *J* = 1.3 Hz, 1H, H6), 7.53 (d, *J* = 1.3 Hz, 1H, H6), 7.36 – 7.31 (m, 2H, H-Ar), 7.31 – 7.25 (m, 2H, H-Ar), 7.24 – 7.19 (m, 5H, H-Ar), 6.90 – 6.82 (m, 4H, H-Ar), 6.41 (t, *J* = 6.5 Hz, 1H, H1′A), 6.19 (dd, *J* = 7.5, 6.3 Hz, 1H, H1′B), 5.55 (q, *J* = 7.4 Hz, 1H, H3′A), 5.32 (d, *J* = 4.3 Hz, 1H, OH), 4.61 (s, 2H, H6′ *and* H6″), 4.36 – 4.29 (m, 1H, H4′A), 4.28 – 4.20 (m, 1H, H3′B), 3.91 – 3.87 (m, 1H, H4′B), 3.72 (s, 6H, 2 × OCH_3), 3.71 – 3.69 (m, 1H, H5′B), 3.64 (dd, *J* = 10.6, 4.2 Hz, 1H, H5″B), 3.32 – 3.25 (m, 2H, H5′A *and* H5″A), 2.80 – 2.73 (m, 2H, H2′A *and* H2″A), 2.17 – 2.02 (m, 2H, H2′B *and* H2″B), 1.61 (d, *J* = 1.3 Hz, 3H, CH_3-*thymine*), 1.59 (d, *J* = 1.3 Hz, 3H, CH_3-*thymine*);

^{13}C NMR (126 MHz, DMSO-*d*$_6$) δ 163.7 (C4), 163.7 (C4), 158.2 (C-Ar), 150.4 (C2), 150.4 (C2), 144.6 (C-Ar), 144.0 (HC=C̲-*triazole*), 136.3 (C6), 135.8 (C6), 135.2 ([a]C-Ar), 135.2 ([a]C-Ar), 129.7 (CH-Ar), 127.9 (CH-Ar), 127.6 (CH-Ar), 126.8 (CH-Ar), 123.3 (HC̲=C-*triazole*), 113.2 (CH-Ar), 109.8 (C5), 109.6 (C5), 86.5 (C_{quat}-*DMTr*), 85.3 (C4′B), 83.9 (C1′A), 83.8 (C1′B), 82.3 (C4′A), 70.7 (C3′B), 70.2 (C5′B), 63.9 (C6′), 63.0 (C5′A), 59.3 (C3′A), 55.9 (OCH_3), 39.5 (C2′B *under DMSO-d$_6$ peaks*), 37.2 (C2′A), 12.0 (CH_3-*thymine*), 11.9 (CH_3-*thymine*); [a]Note that the marked carbons can resolve in distinct peaks due to different environments.

HRMS–ESI (*m/z*): $[M + Na]^+$ calcd for $C_{44}H_{47}N_7O_{11}Na$, 872.3226; found, 872.3223.

5′-*O*-(4,4′-Dimethoxytrityl)-thymidine-1,4-triazole-thymidine dimer phosphoramidite (**9**)

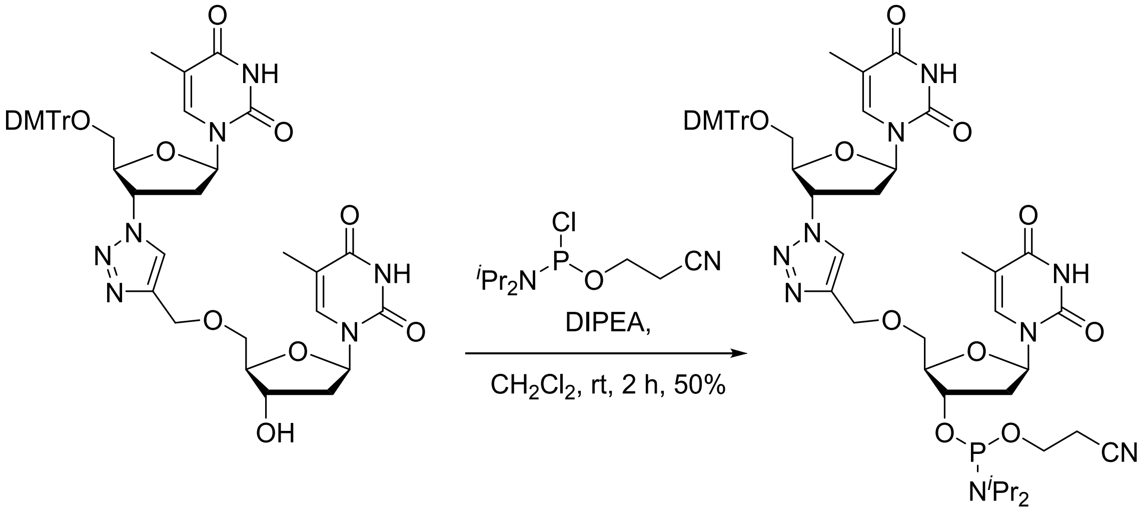

8 **9**

$C_{53}H_{64}N_9O_{12}P$
1050.12

Degassed DIPEA (217 µL, 1.15 mmol) and chloro(diisopropylamino)-β-cyanoethoxyphosphine (186 µL, 0.83 mmol) were added to a solution of dimer **8** (345 mg, 0.41 mmol) in degassed, anhyd CH_2Cl_2 (3 mL) and the reaction mixture was stirred at rt for 2 h. The solution was diluted with degassed CH_2Cl_2 (10 mL) and then washed with sat. aq KCl (10 mL). The aq layer was extracted with degassed CH_2Cl_2 (2 × 10 mL) and the combined organic layers were dried over Na_2SO_4, filtered and the solvents evaporated under reduced pressure. Purification by column chromatography (EtOAc) gave phosphoramidite **9** as a white foam (216 mg, 0.21 mmol, 50%).

***R*$_f$** 0.34 (EtOAc);

^{31}P NMR (162 MHz, $CDCl_3$) δ 148.7, 148.6;

HRMS–ESI (*m/z*): [M + Na]$^+$ calcd for $C_{53}H_{64}N_9O_{12}PNa$, 1072.4304; found, 1072.4298.

UPLC-MS analysis and NMR spectra

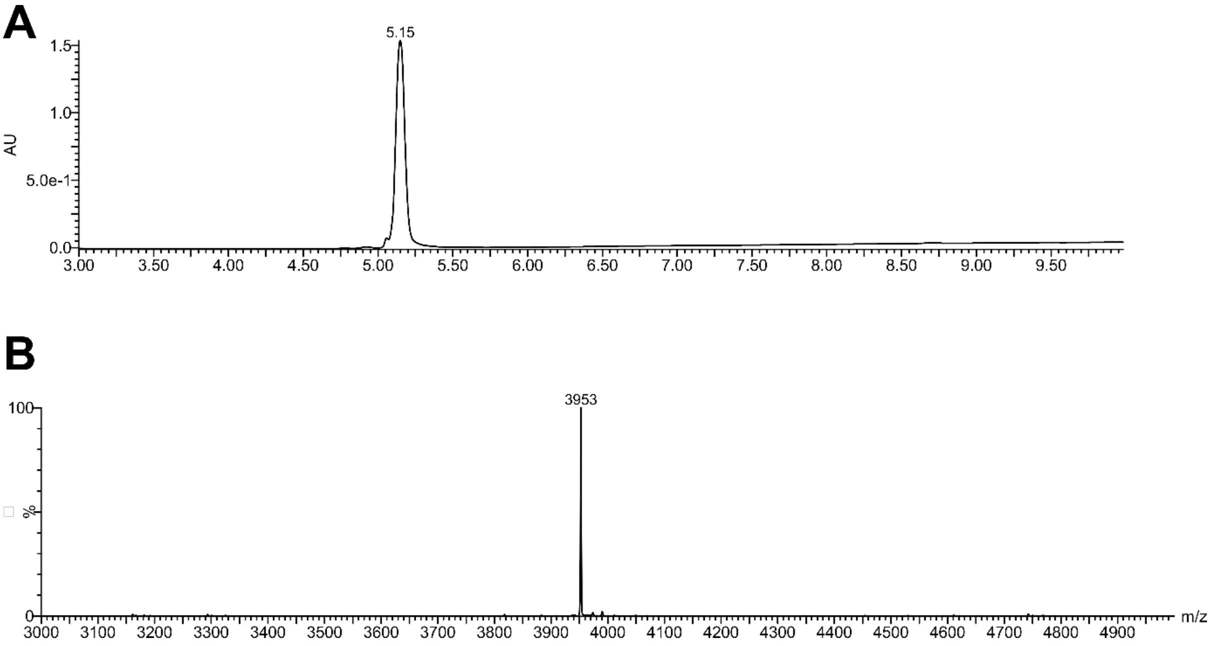

Figure S25. (A) RP-UPLC trace of ON2 (TL**2**). Conditions: BEH C18 column, 130 Å (1.7 µm, 2.1 mm × 50 mm), 0.2 mL·min^{-1}; Method: 0–70% B; 8 min. (B) De-convoluted mass spectrum for ON2 (TL**2**).

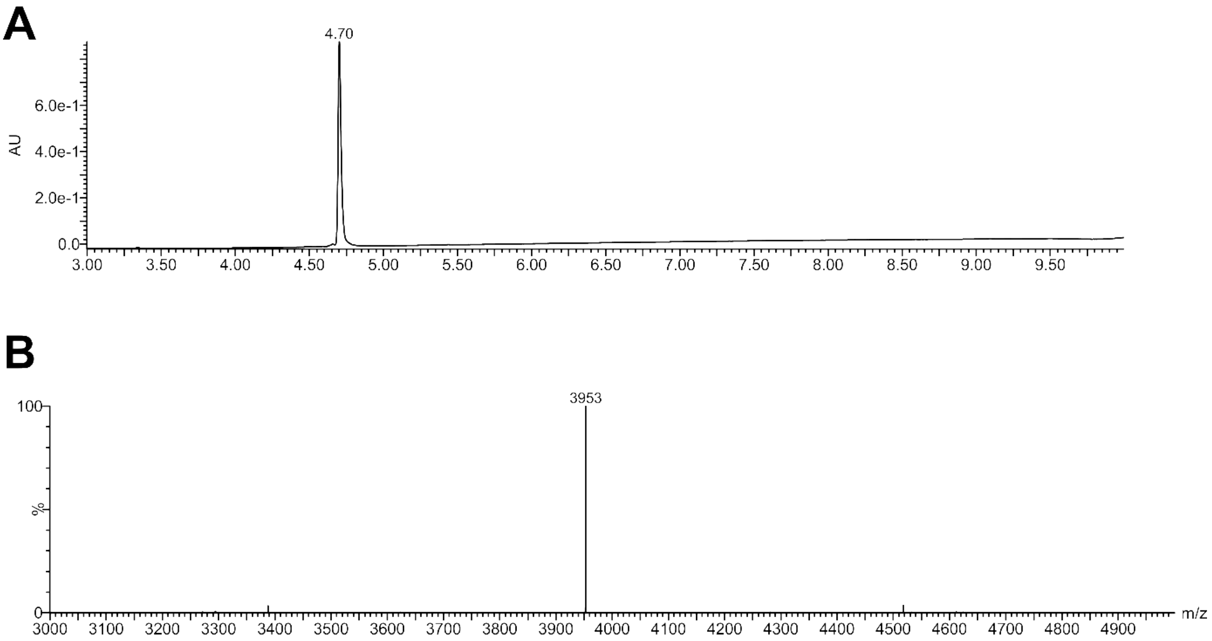

Figure S26. (A) RP-UPLC trace of ON5 (TL**5**). Conditions: BEH C18 column, 130 Å (1.7 µm, 2.1 mm × 50 mm), 0.2 mL·min^{-1}; Method: 0–70% B; 8 min. (B) De-convoluted mass spectrum for ON5 (TL**5**).

A

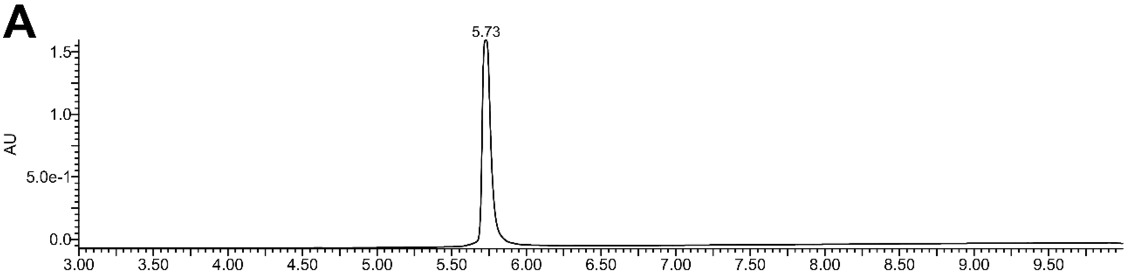

B

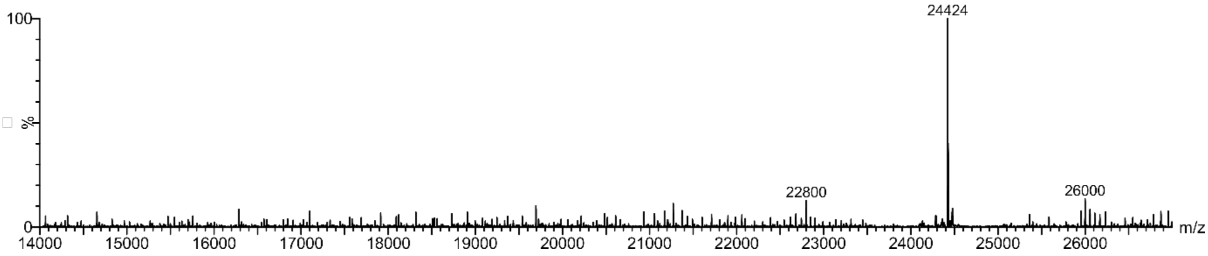

Figure S27. (A) RP-UPLC trace of Template 1 (TL**1**). Conditions: BEH C18 column, 130 Å (1.7 μm, 2.1 mm × 50 mm), 0.2 mL·min^{-1}; Method: 0–70% B; 8 min. (B) De-convoluted mass spectrum for Template 1 (TL**1**).

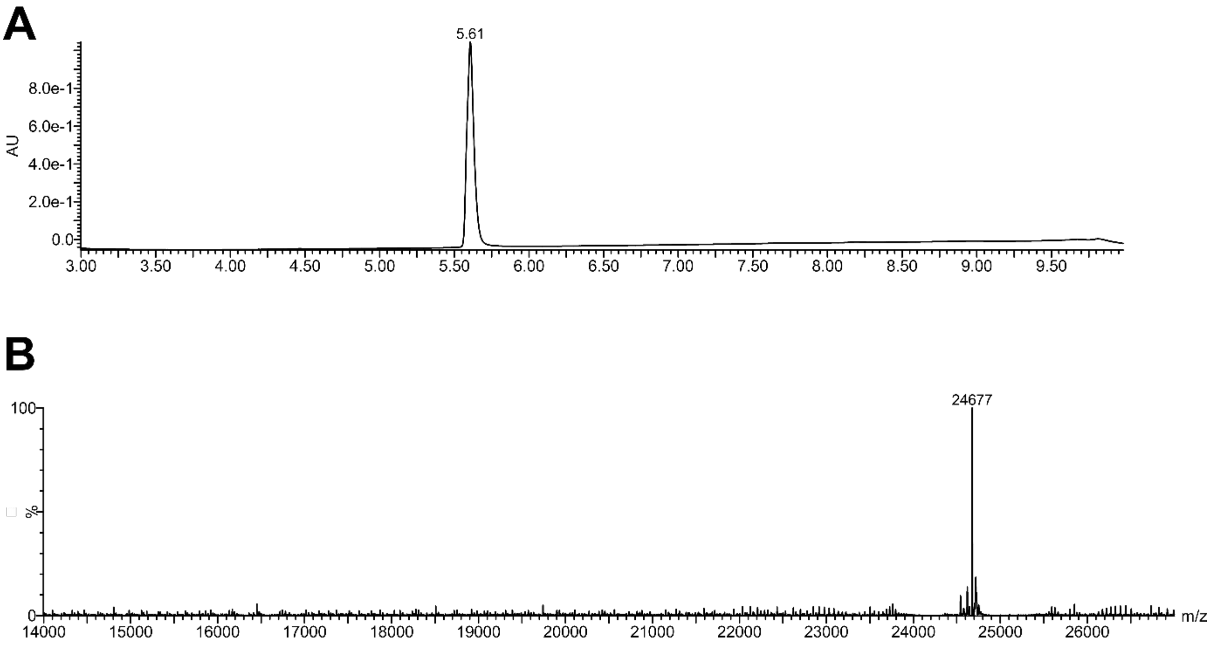

Figure S28. (A) RP-UPLC trace of Template 2 (TL**2**). Conditions: BEH C18 column, 130 Å (1.7 µm, 2.1 mm × 50 mm), 0.2 mL·min^{-1}; Method: 0–70% B; 8 min. (B) De-convoluted mass spectrum for Template 2 (TL**2**).

A

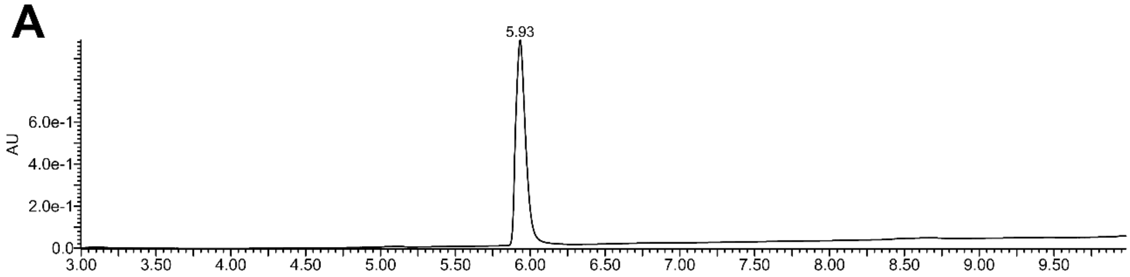

B

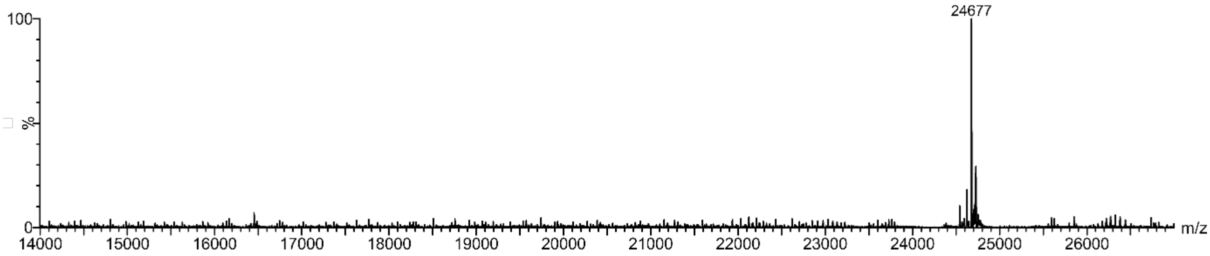

Figure S29. (A) RP-UPLC trace of Template 3 (TL**3**). Conditions: BEH C18 column, 130 Å (1.7 µm, 2.1 mm × 50 mm), 0.2 mL·min^{-1}; Method: 0–70% B; 8 min. (B) De-convoluted mass spectrum for Template 3 (TL**3**).

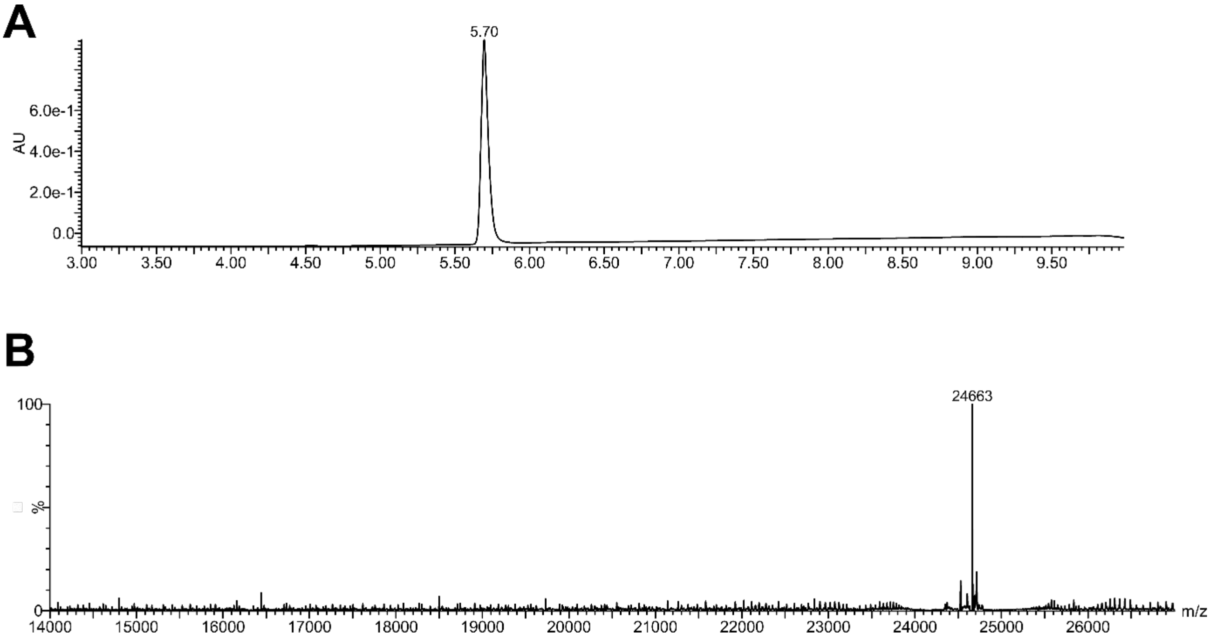

Figure S30. (A) RP-UPLC trace of Template 4 (TL**4**). Conditions: BEH C18 column, 130 Å (1.7 µm, 2.1 mm × 50 mm), 0.2 mL·min^{-1}; Method: 0–70% B; 8 min. (B) De-convoluted mass spectrum for Template 4 (TL**4**).

A

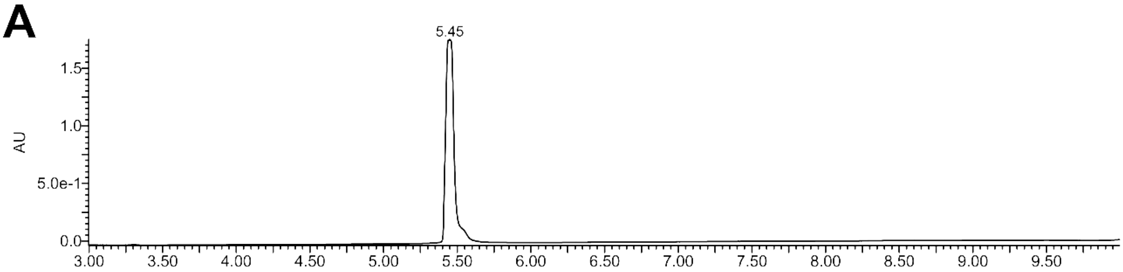

B

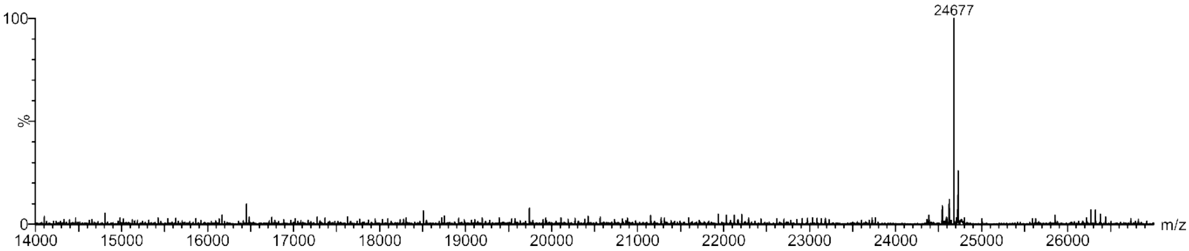

Figure S31. (A) RP-UPLC trace of Template 5 (TL**5**). Conditions: BEH C18 column, 130 Å (1.7 µm, 2.1 mm × 50 mm), 0.2 mL·min^{-1}; Method: 0–70% B; 8 min. (B) De-convoluted mass spectrum for Template 5 (TL**5**).

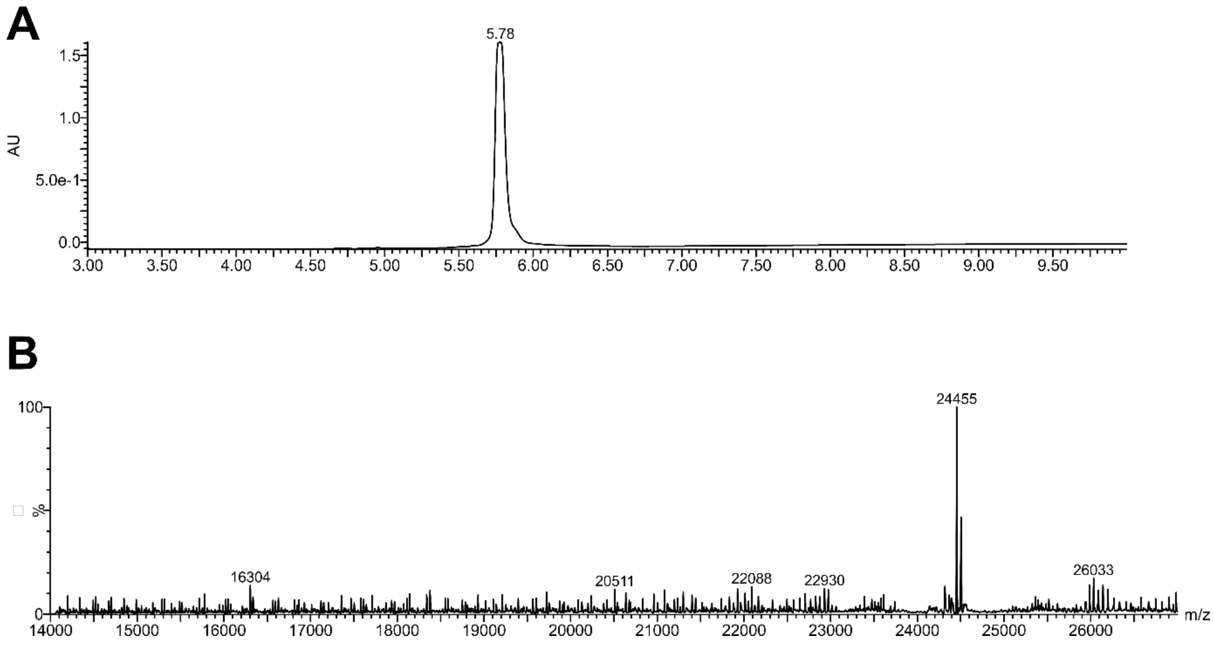

Figure S32. (A) RP-UPLC trace of Template 6 (TL**6**). Conditions: BEH C18 column, 130 Å (1.7 μm, 2.1 mm × 50 mm), 0.2 mL·min^{-1}; Method: 0–70% B; 8 min. (B) De-convoluted mass spectrum for Template 6 (TL**6**).

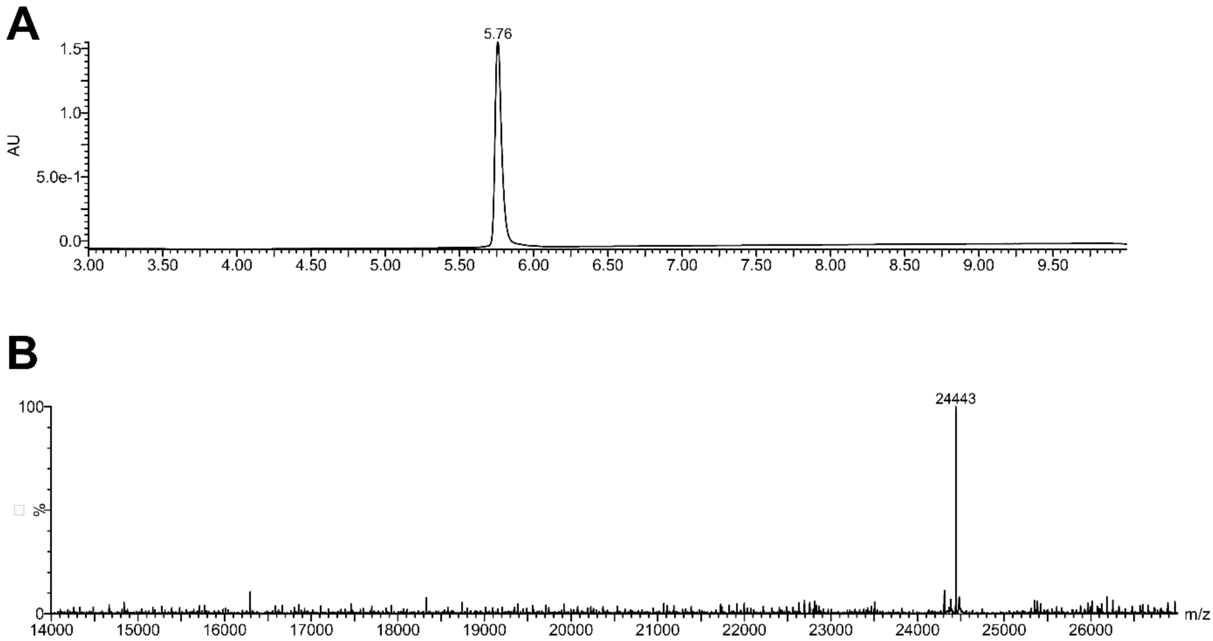

Figure S33. (A) RP-UPLC trace of Template 7 (TL**7**). Conditions: BEH C18 column, 130 Å (1.7 µm, 2.1 mm × 50 mm), 0.2 mL·min^{-1}; Method: 0–70% B; 8 min. (B) De-convoluted mass spectrum for Template 7 (TL**7**).

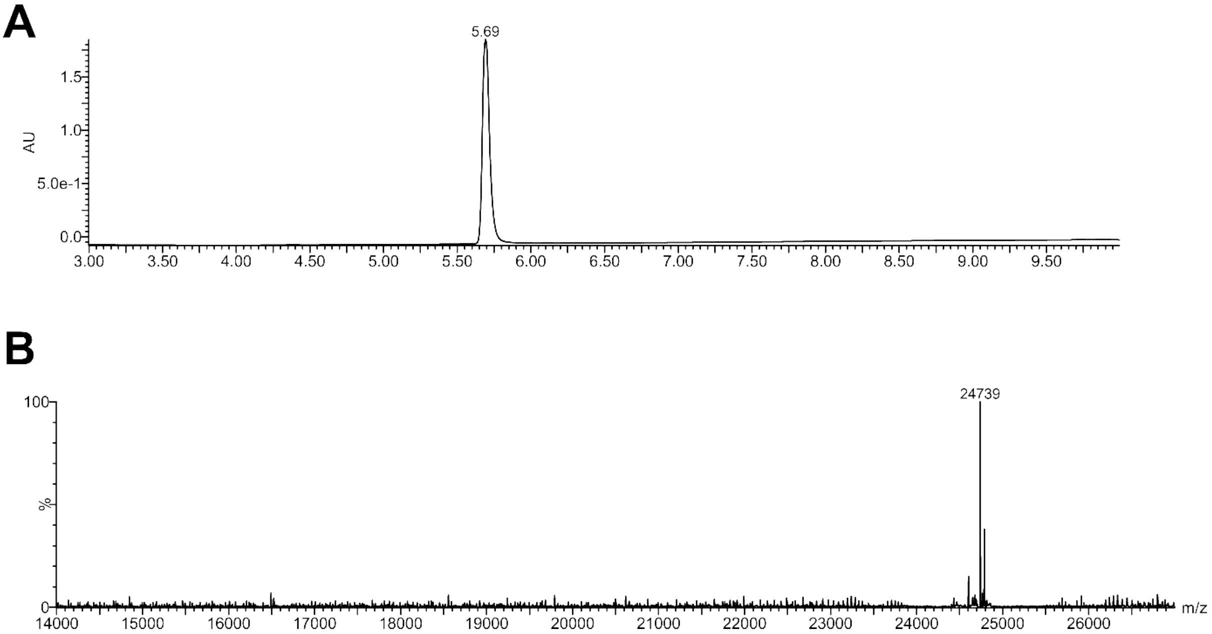

Figure S34. (A) RP-UPLC trace of Template 9 (TL**2**). Conditions: BEH C18 column, 130 Å (1.7 µm, 2.1 mm × 50 mm), 0.2 mL·min^{-1}; Method: 0–70% B; 8 min. (B) De-convoluted mass spectrum for Template 9 (TL**2**).

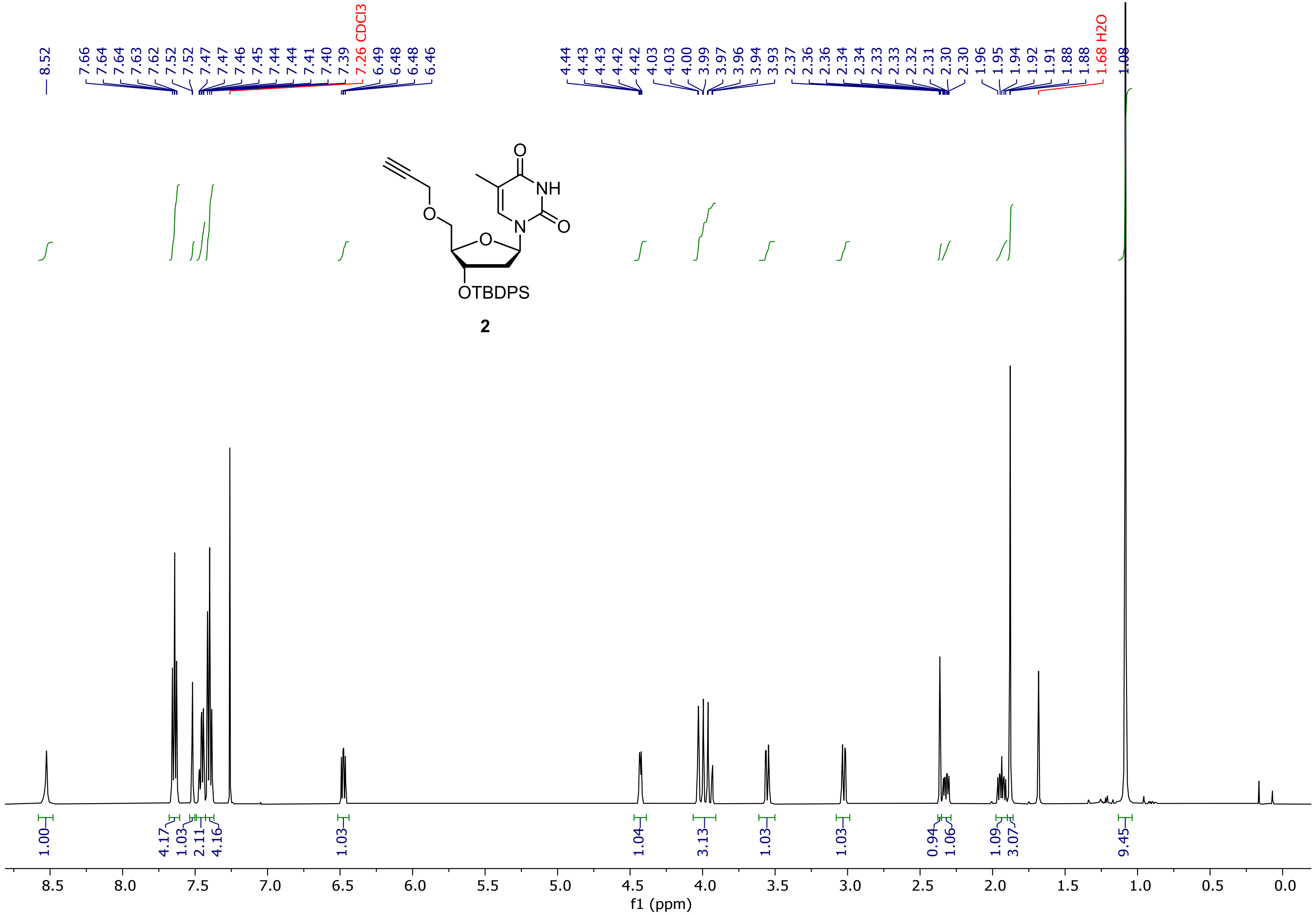

Figure S35. ^{1}H NMR ($CDCl_3$) of **2**.

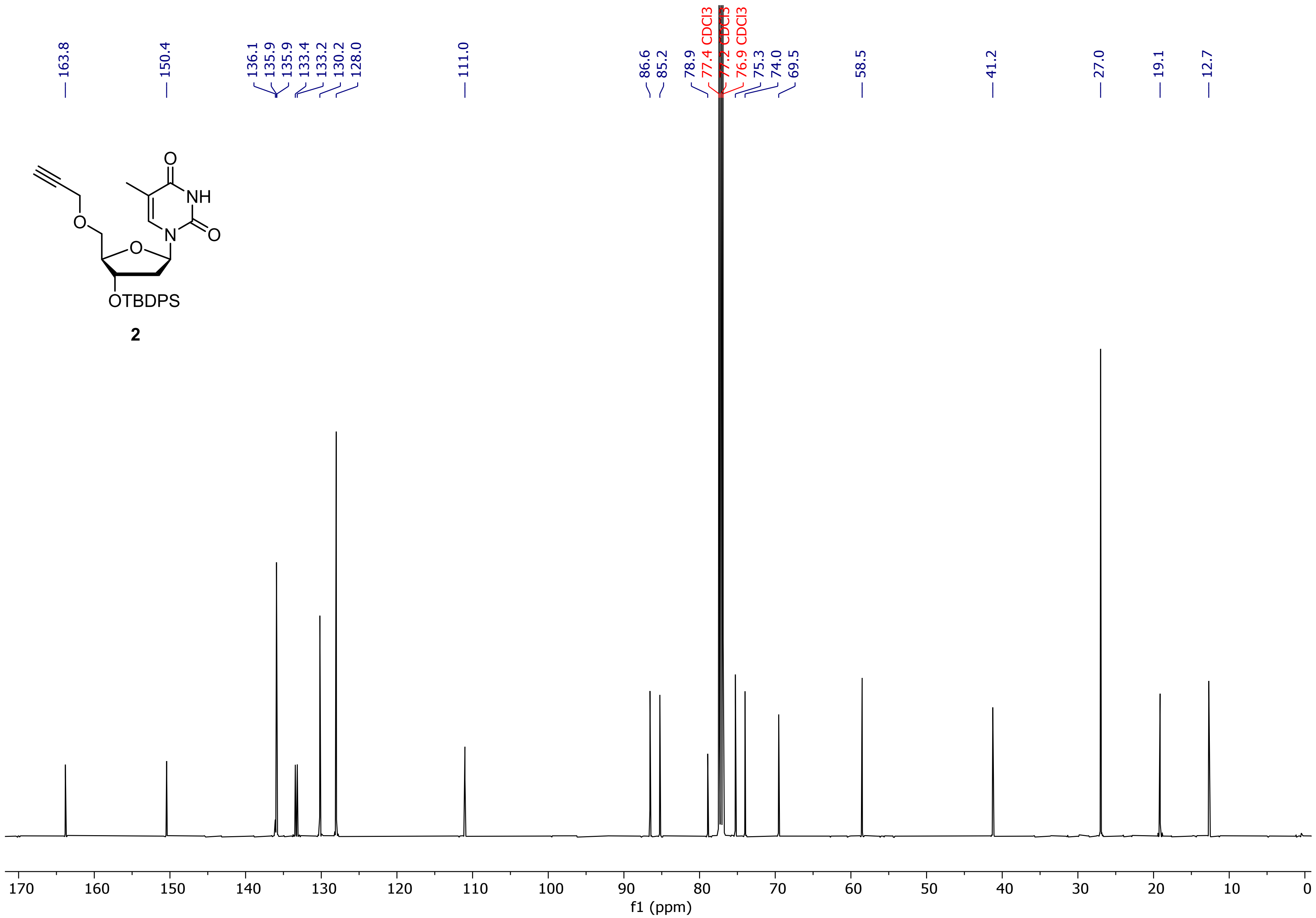

Figure S36. ^{13}C NMR ($CDCl_3$) of **2**.

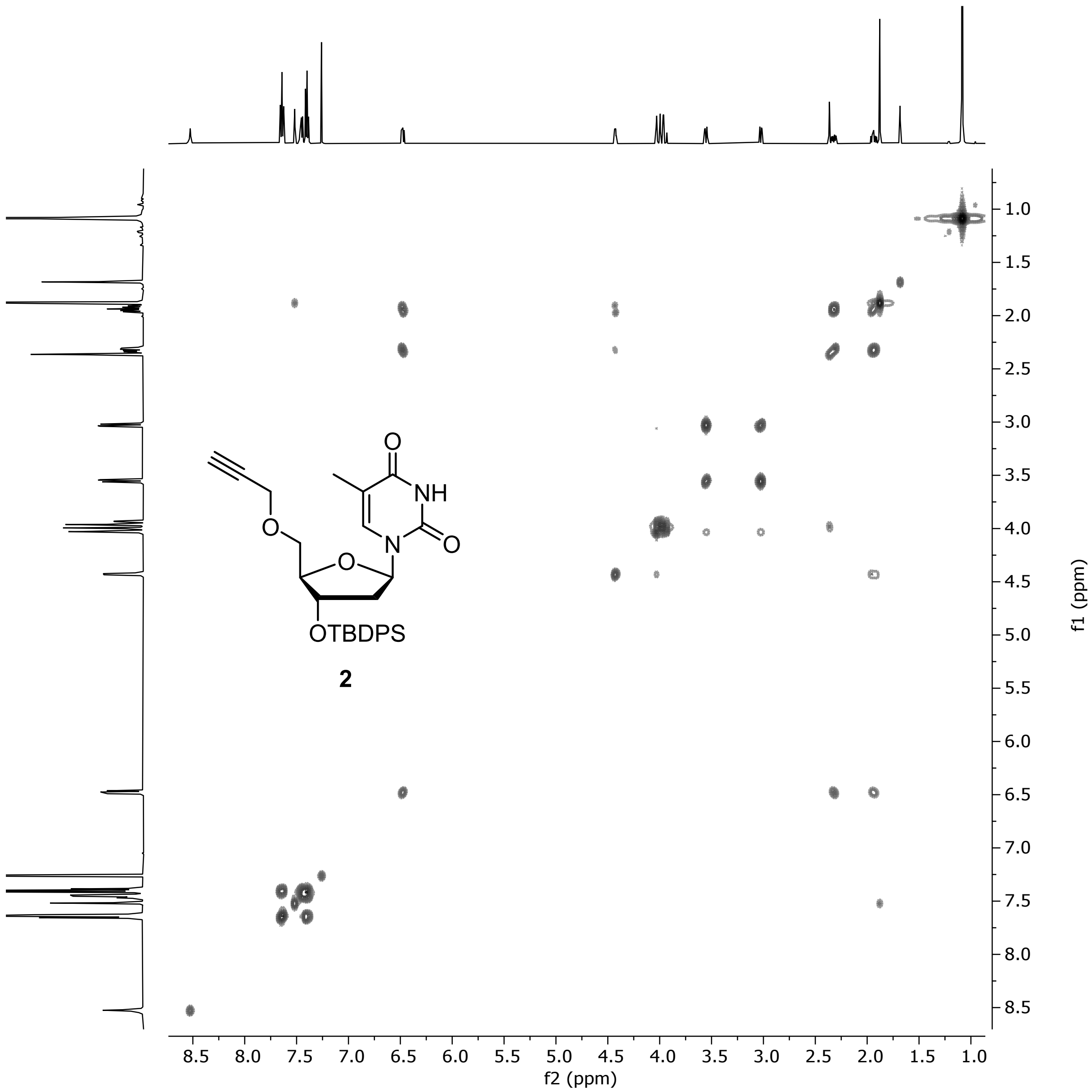

Figure S37. COSY NMR ($CDCl_3$) of **2**.

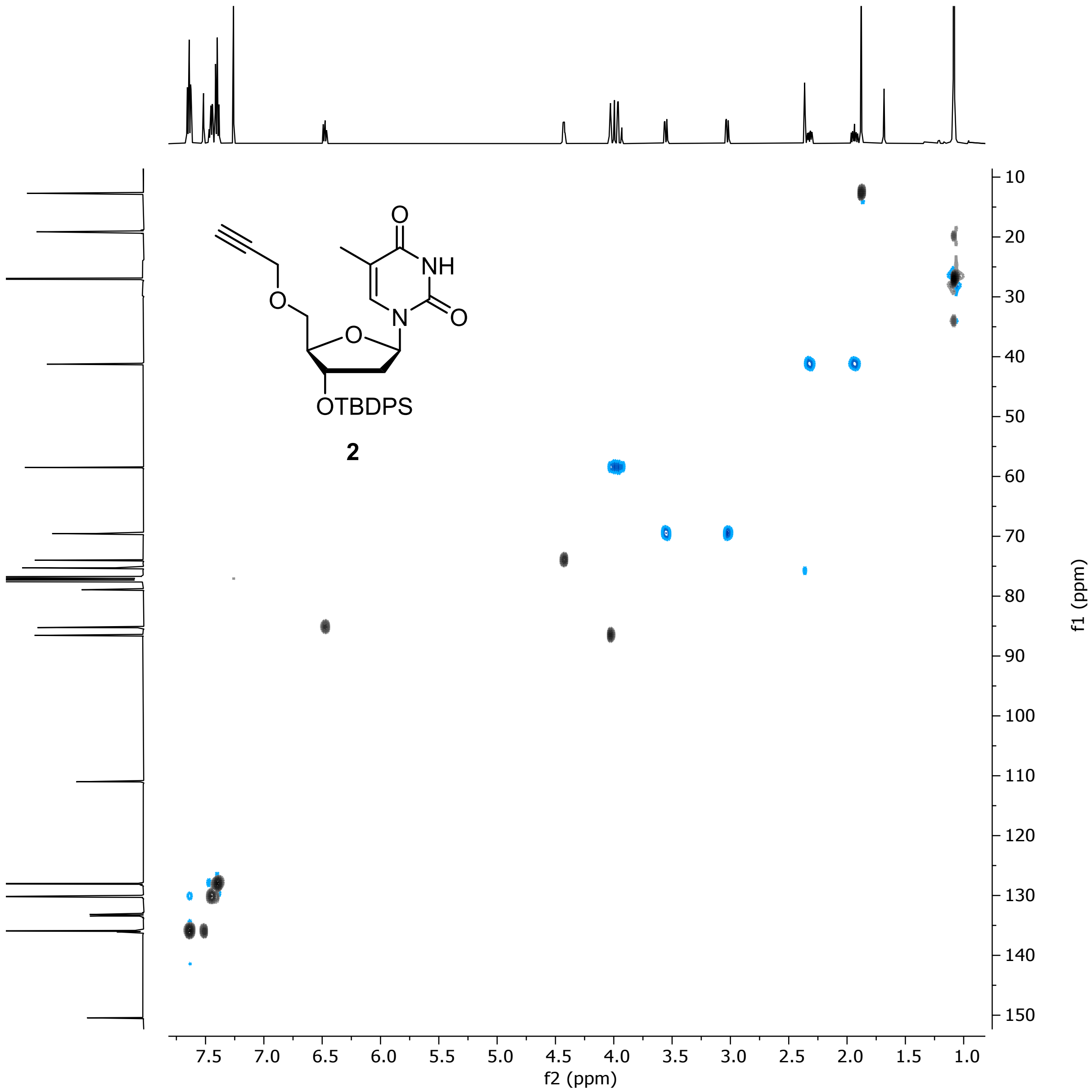

Figure S38. HSQC NMR ($CDCl_3$) of **2**.

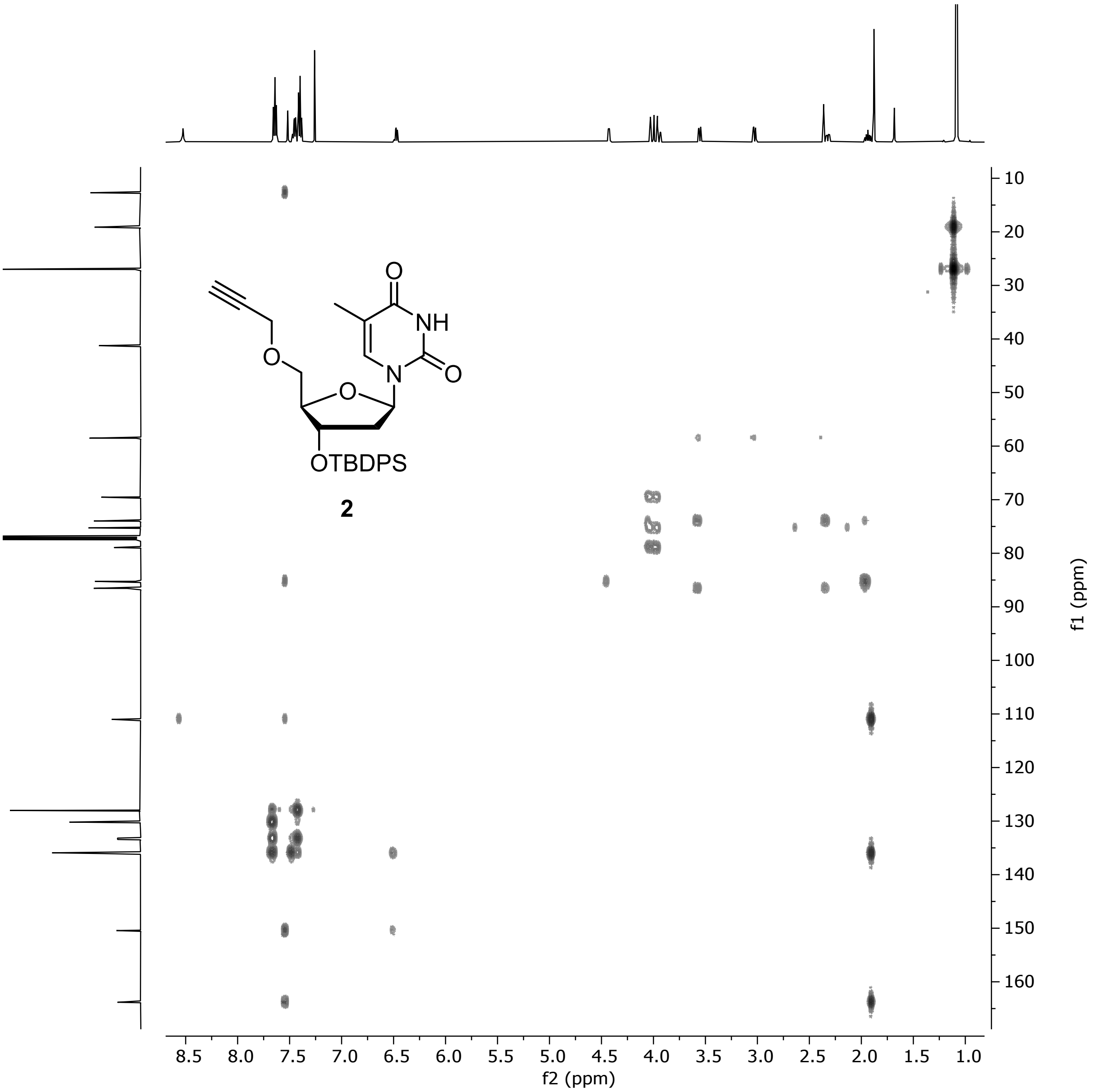

Figure S39. HMBC NMR ($CDCl_3$) of **2**.

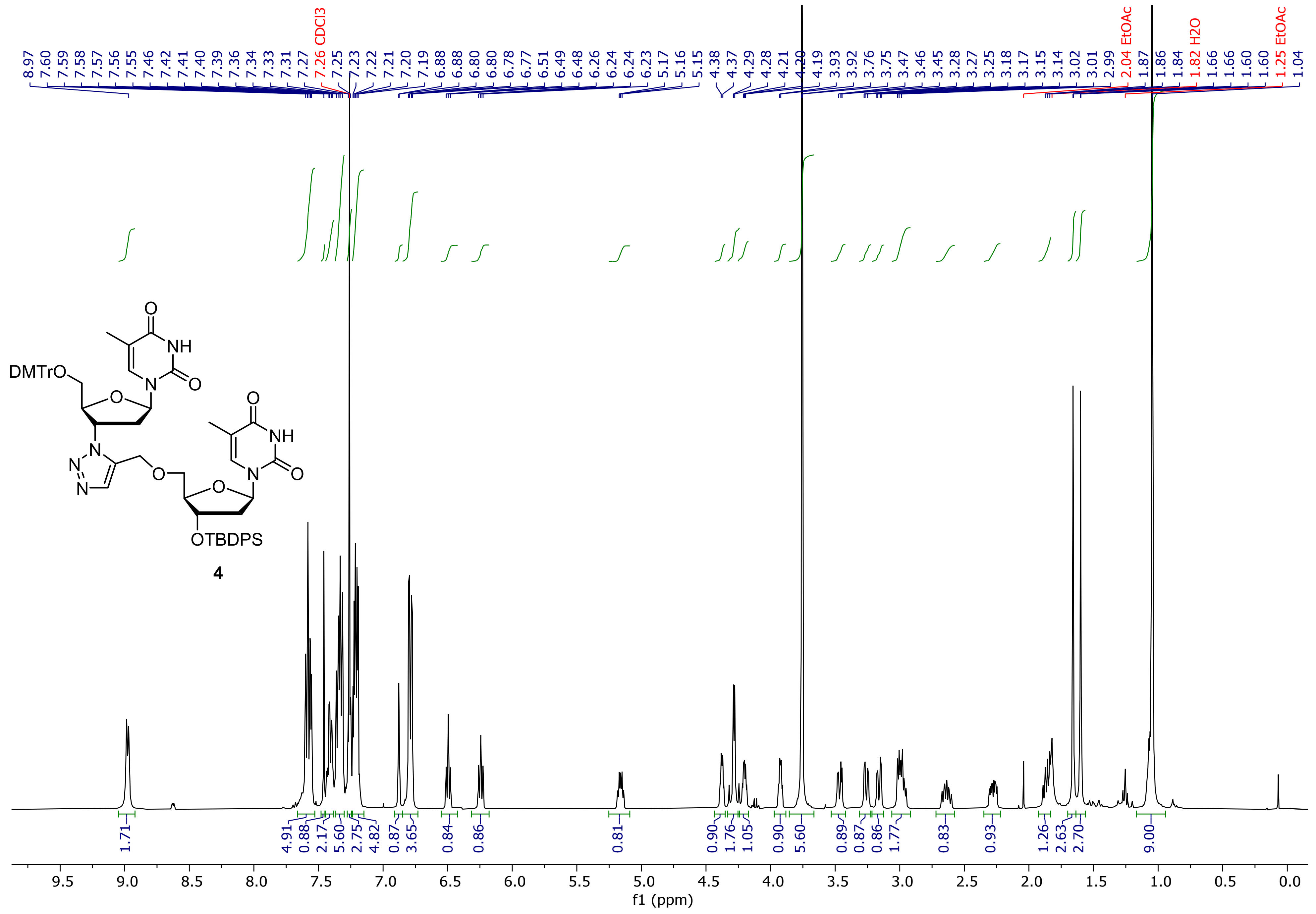

Figure S40. ^{1}H NMR ($CDCl_3$) of **4**.

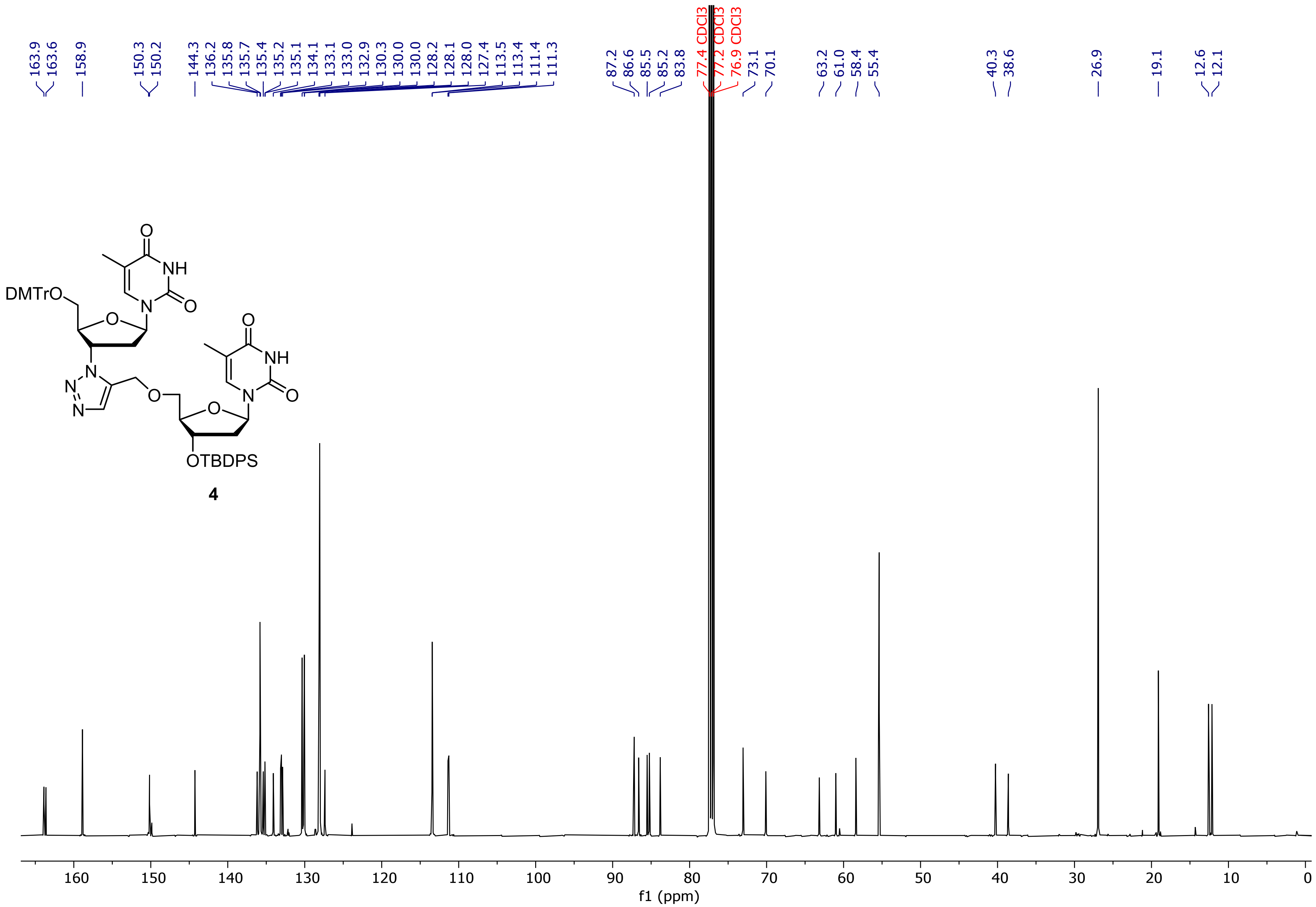

Figure S41. ^{13}C NMR ($CDCl_3$) of **4**.

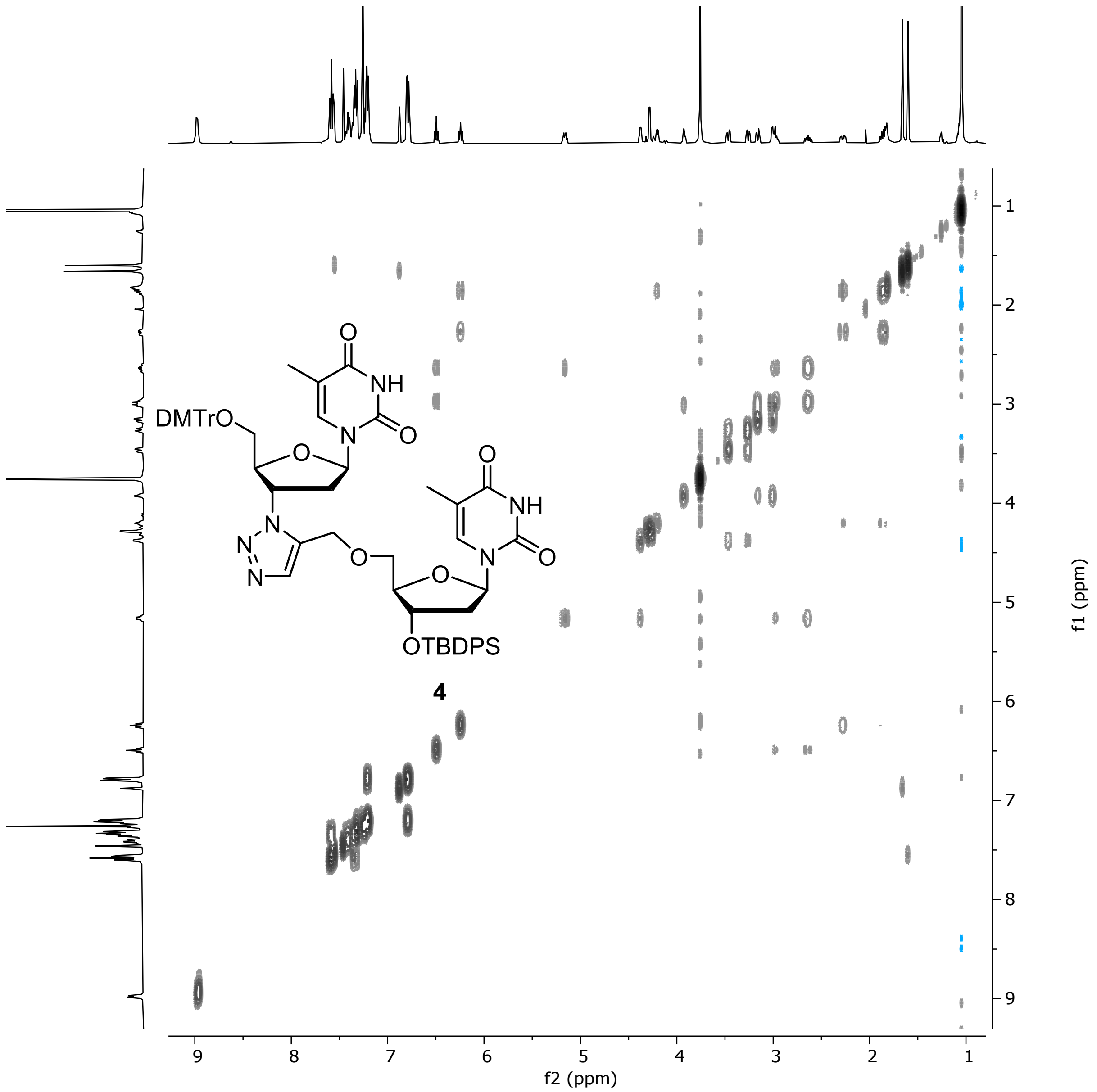

Figure S42. COSY NMR ($CDCl_3$) of **4**.

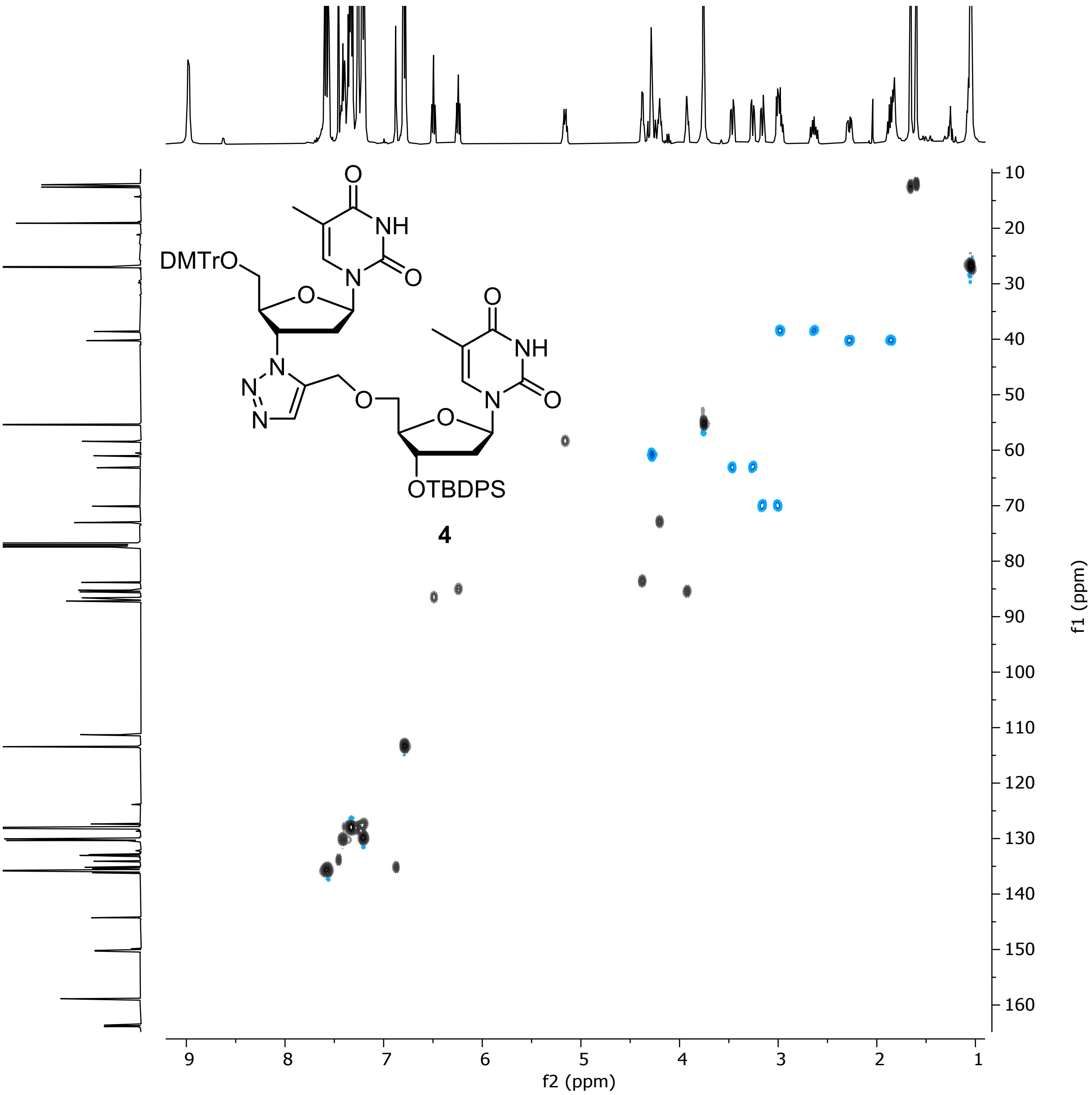

Figure S43. HSQC NMR ($CDCl_3$) of **4**.

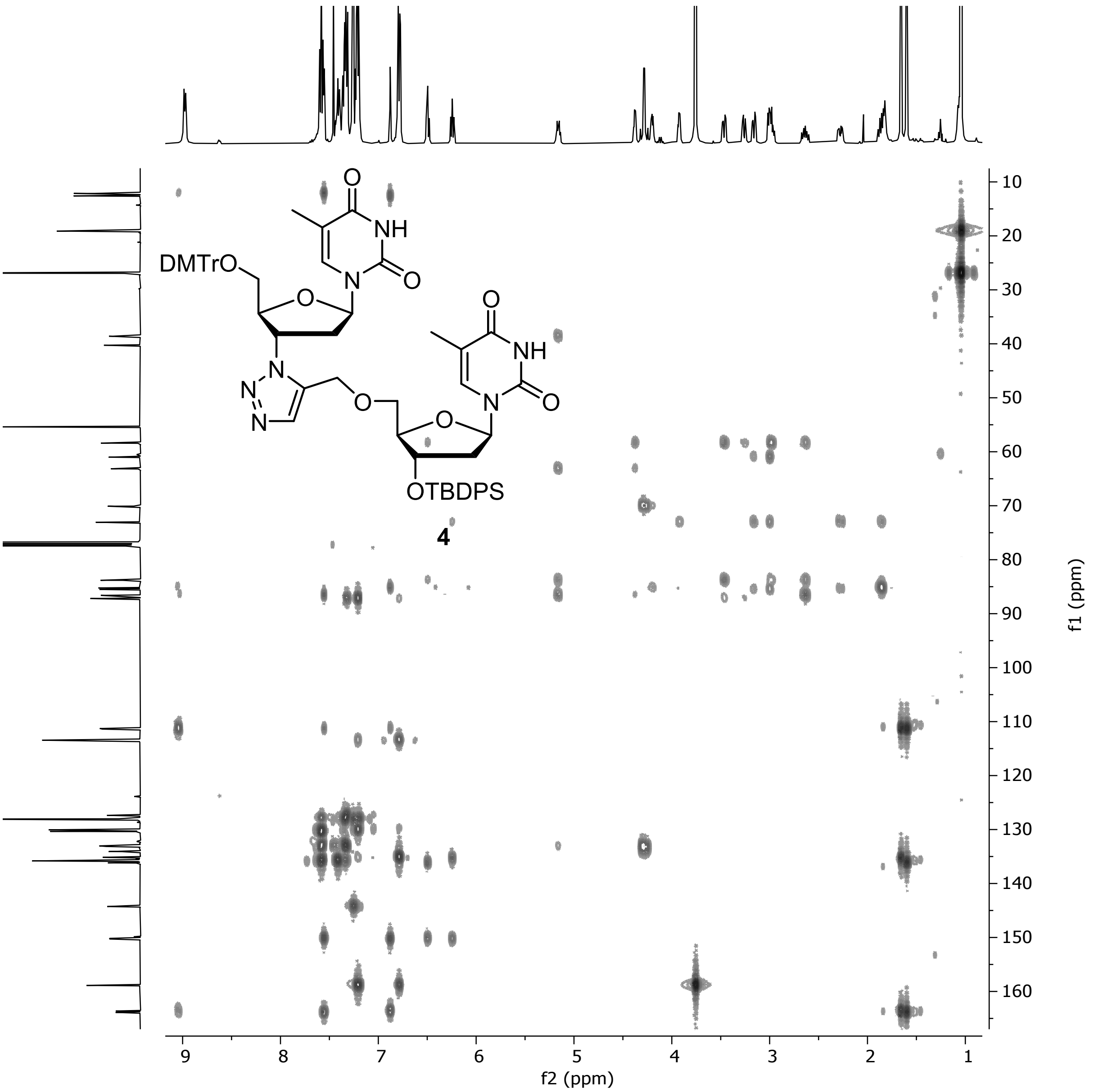

Figure S44. HMBC NMR ($CDCl_3$) of **4**.

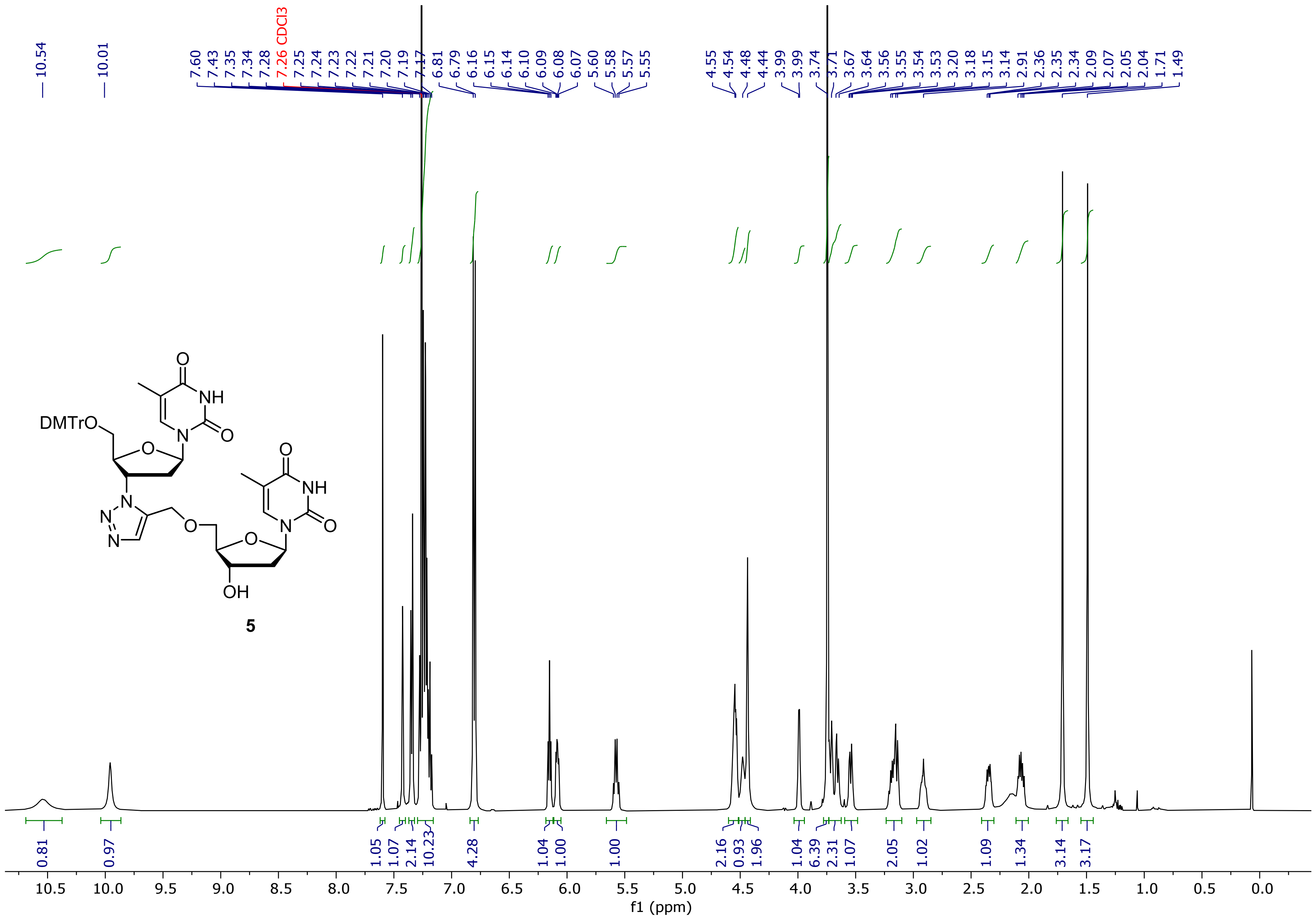

Figure S45. ^{1}H NMR ($CDCl_3$) of **5**.

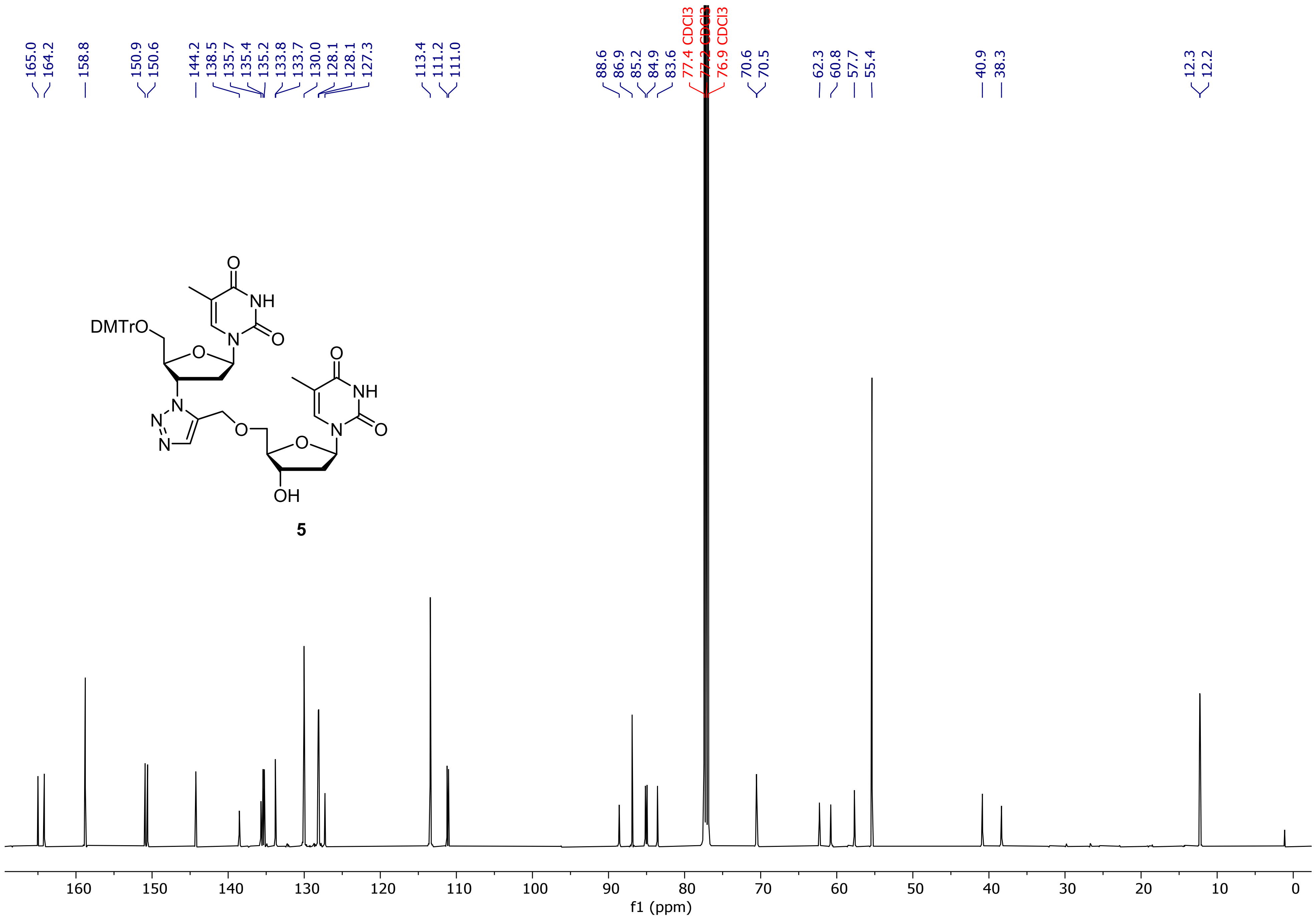

Figure S46. ^{13}C NMR ($CDCl_3$) of **5**.

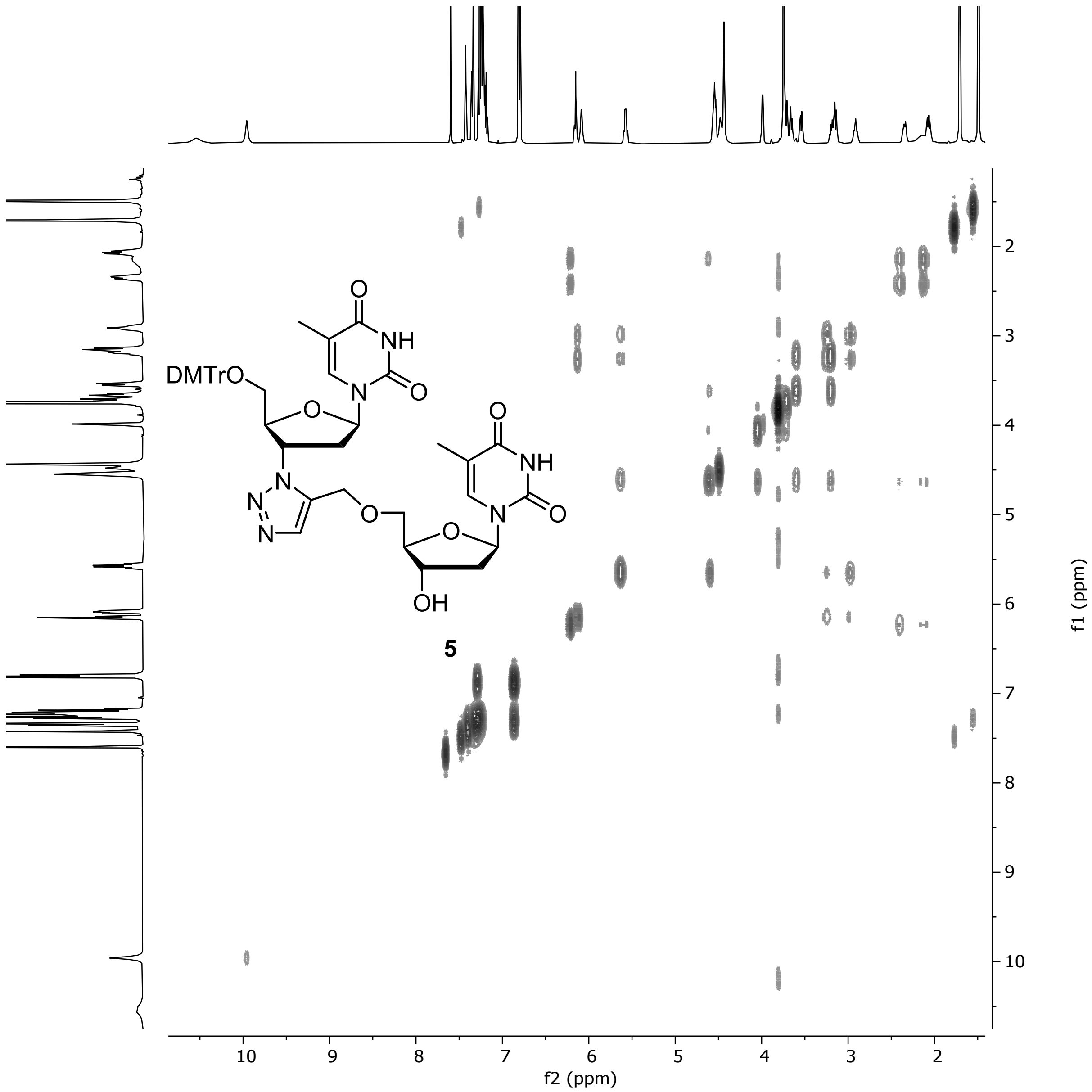

Figure S47. COSY NMR ($CDCl_3$) of **5**.

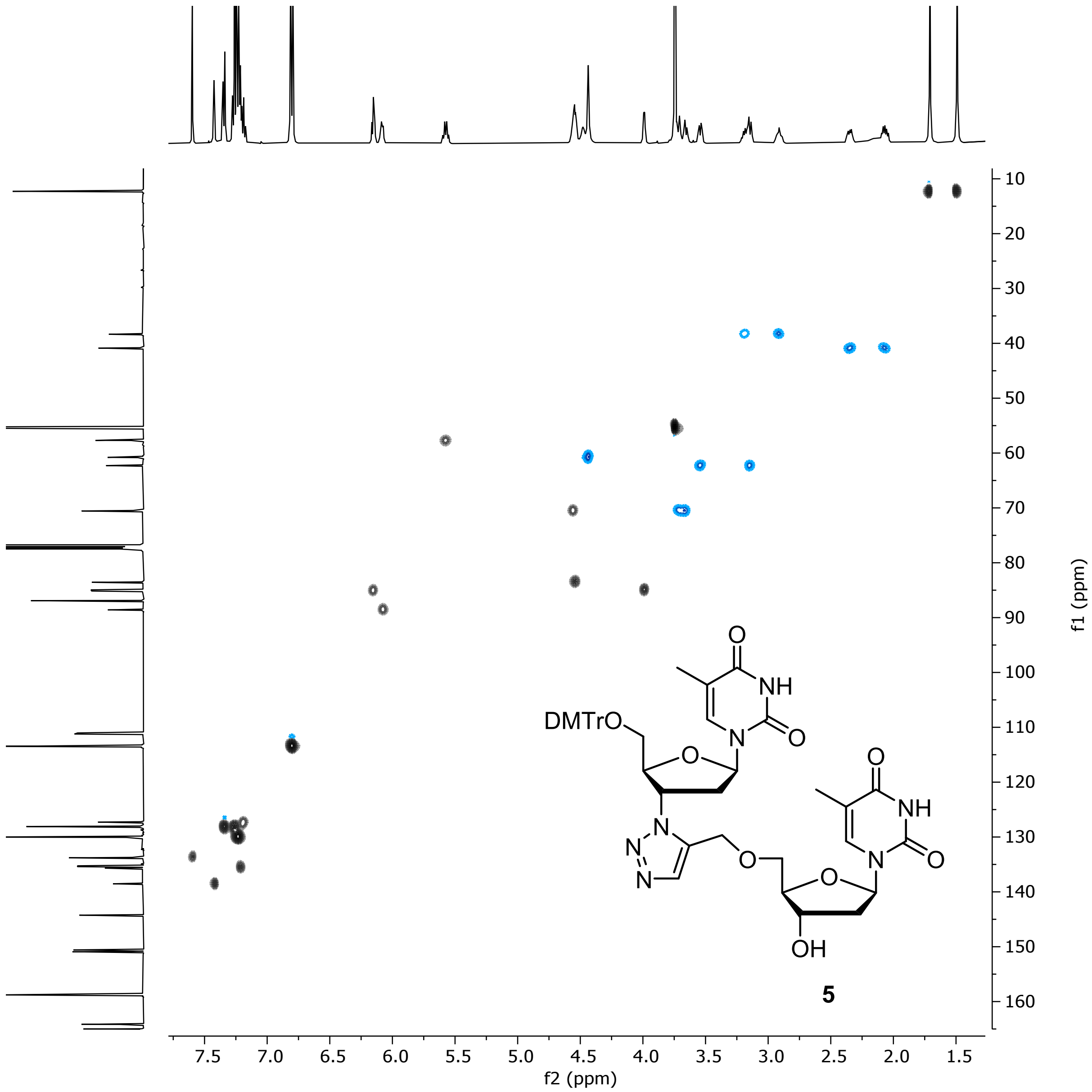

Figure S48. HSQC NMR ($CDCl_3$) of **5**.

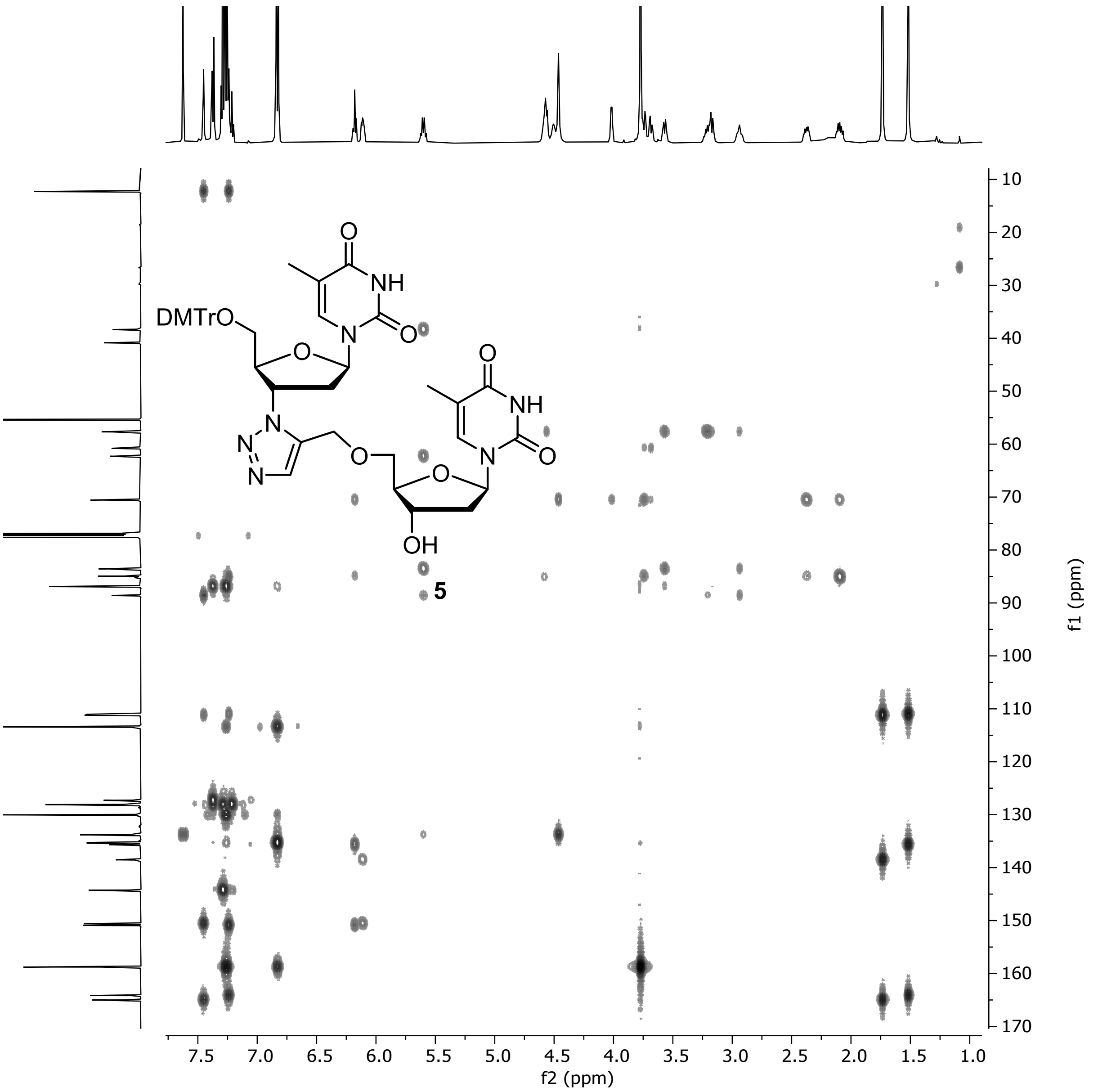

Figure S49. HMBC NMR ($CDCl_3$) of **5**.

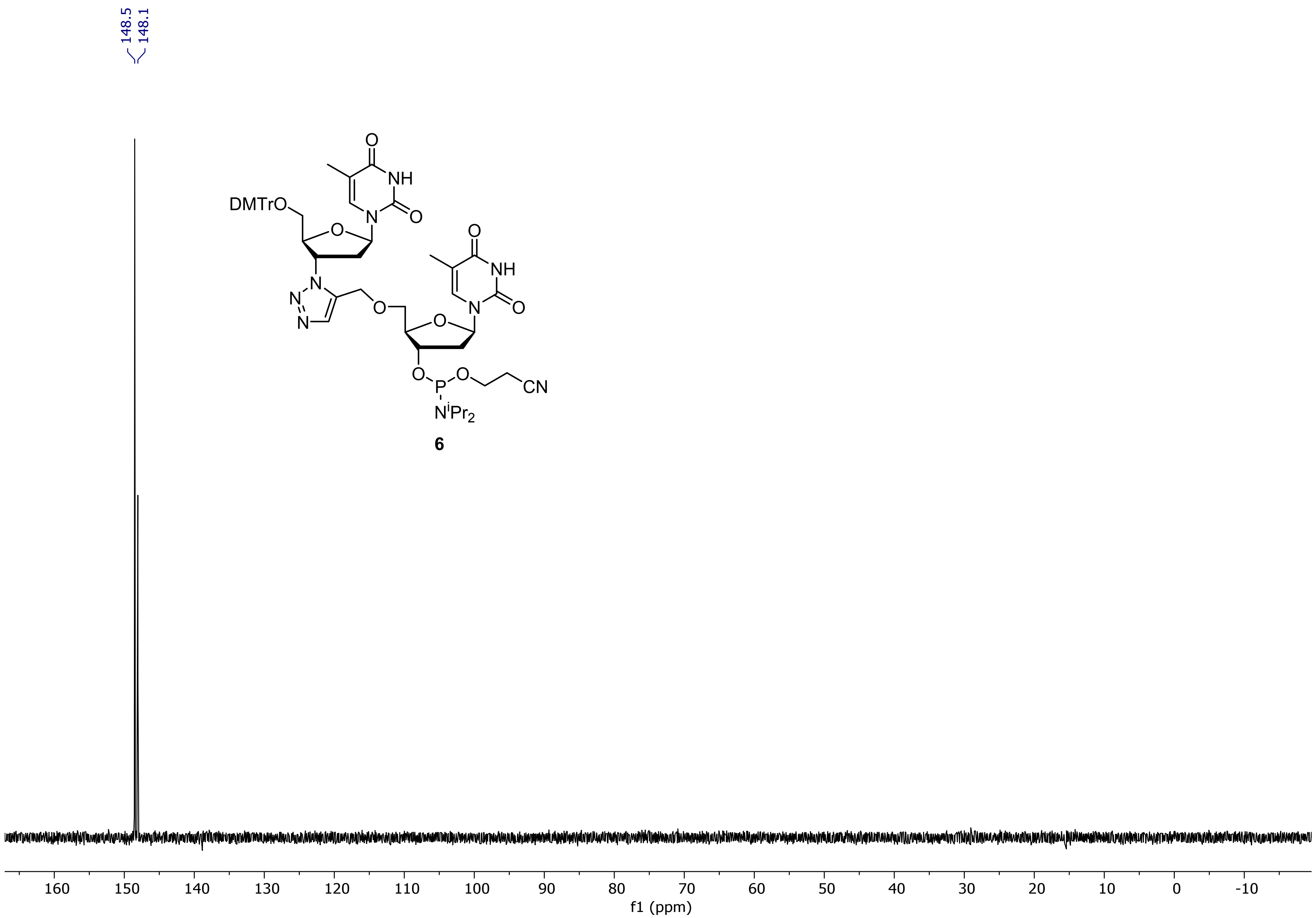

Figure S50. ^{31}P NMR ($CDCl_3$) of **6**.

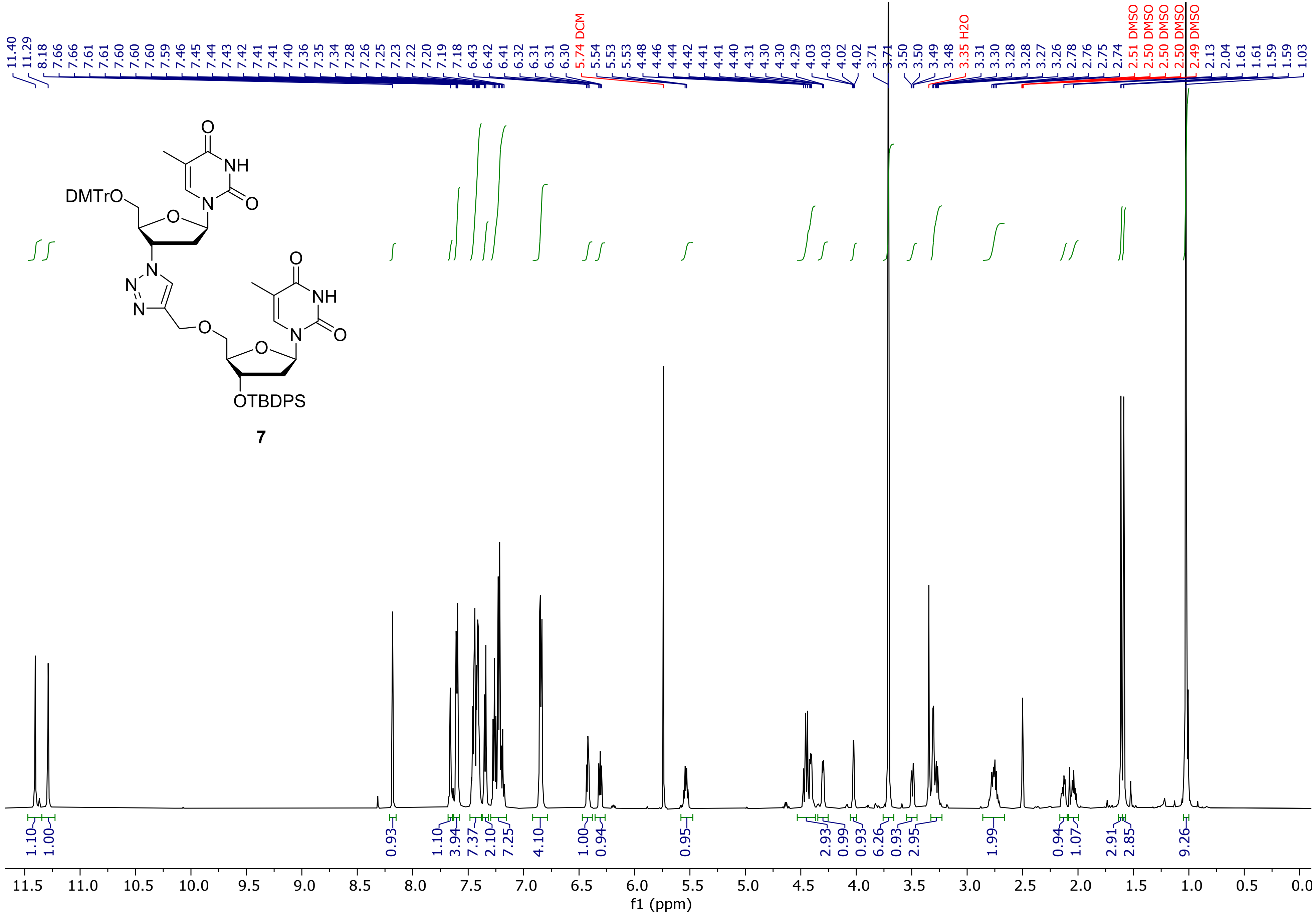

Figure S51. ^{1}H NMR (DMSO-d_6) of **7**.

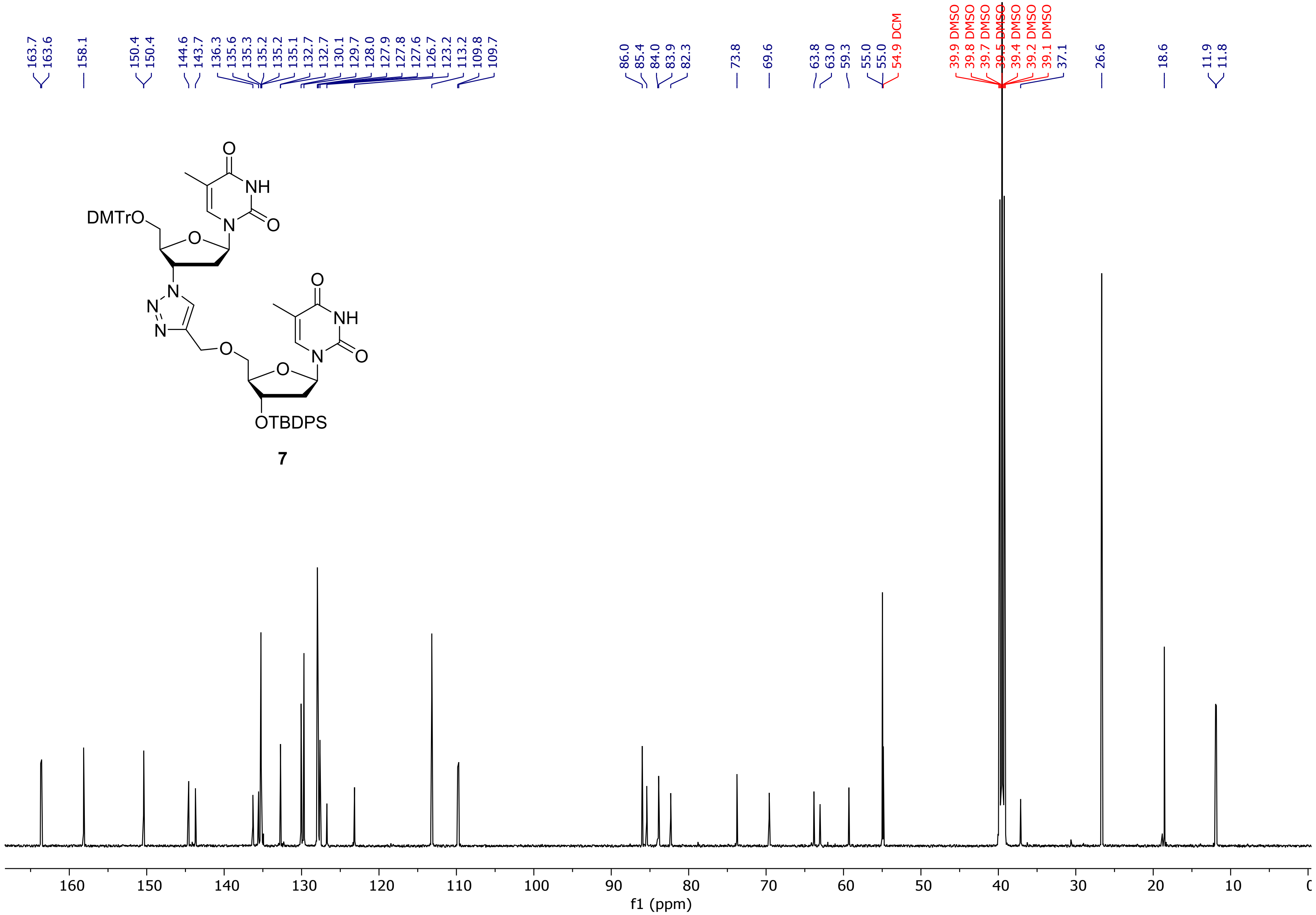

Figure S52. ^{13}C NMR (DMSO-d_6) of **7**.

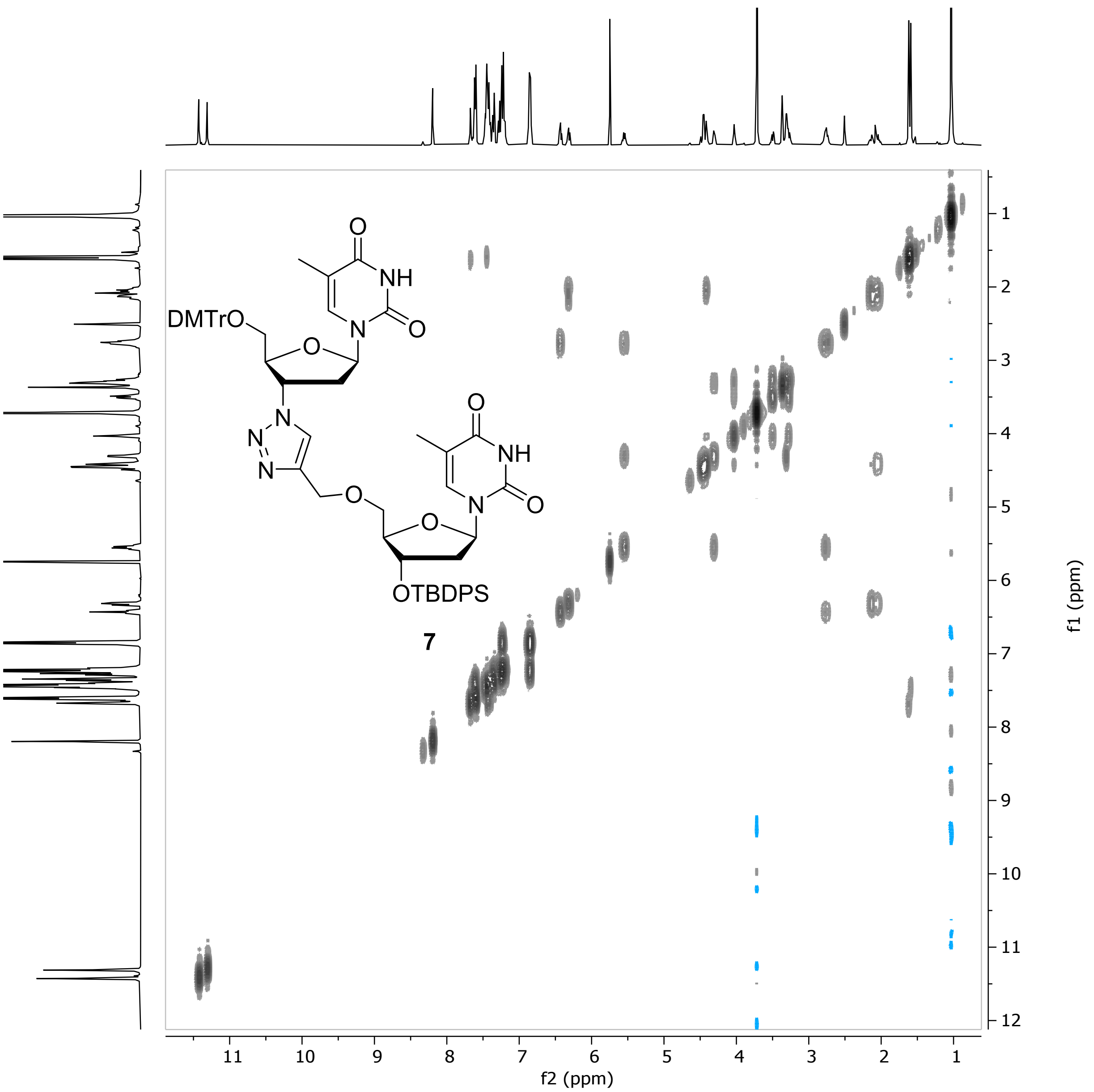

Figure S53. COSY NMR (DMSO-d_6) of **7**.

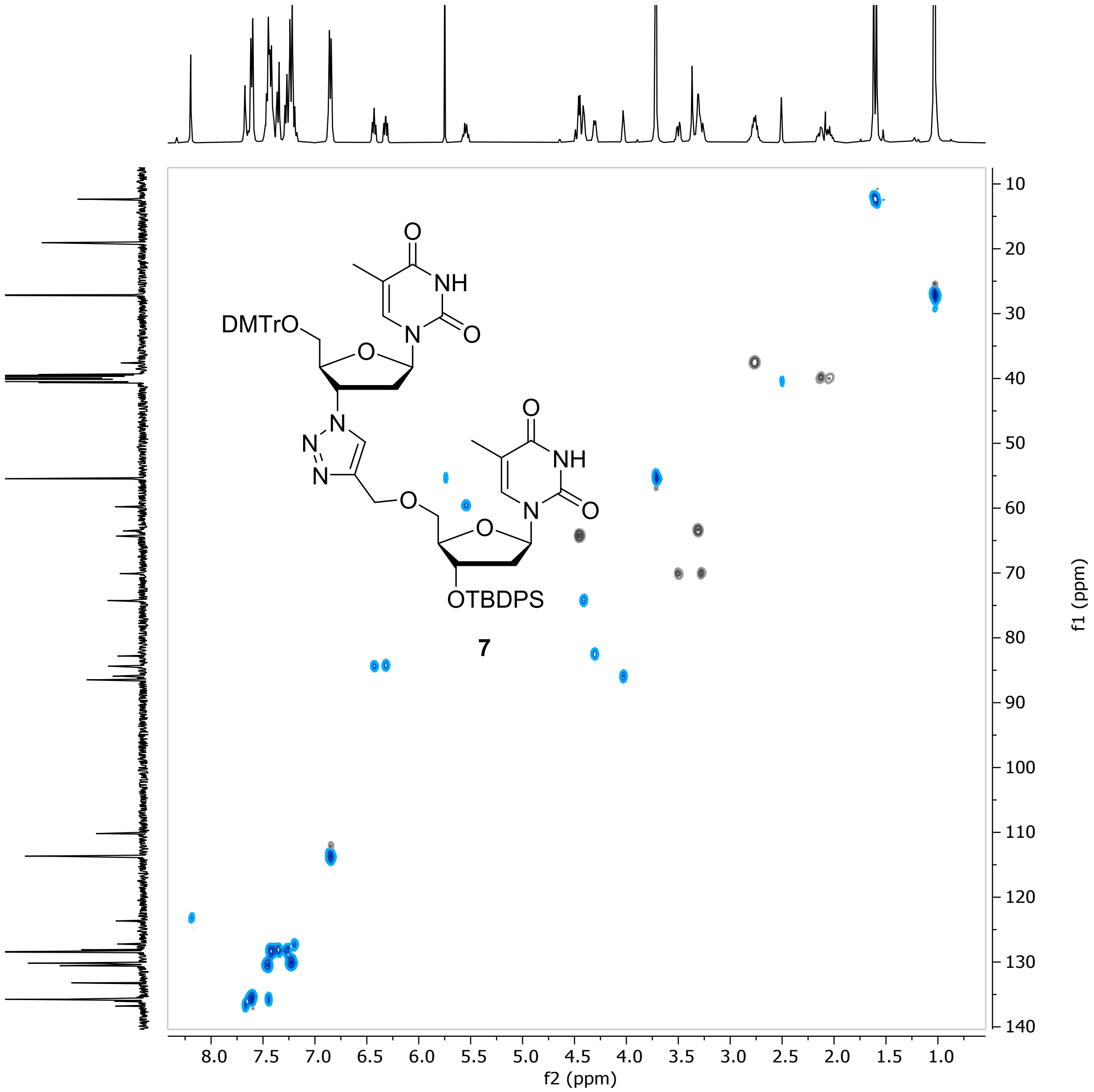

Figure S54. HSQC NMR (DMSO-d_6) of **7**.

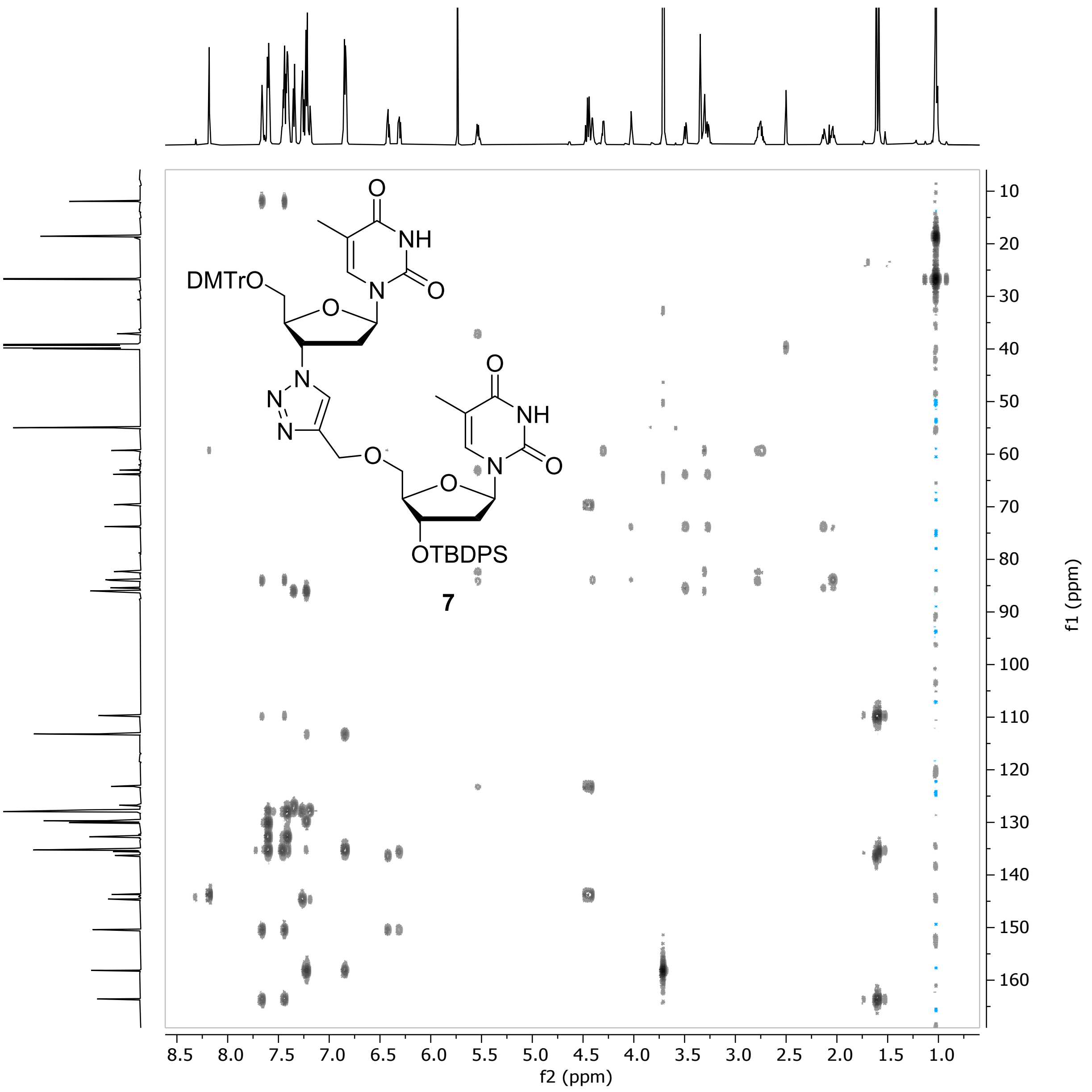

Figure S55. HMBC NMR (DMSO-d_6) of **7**.

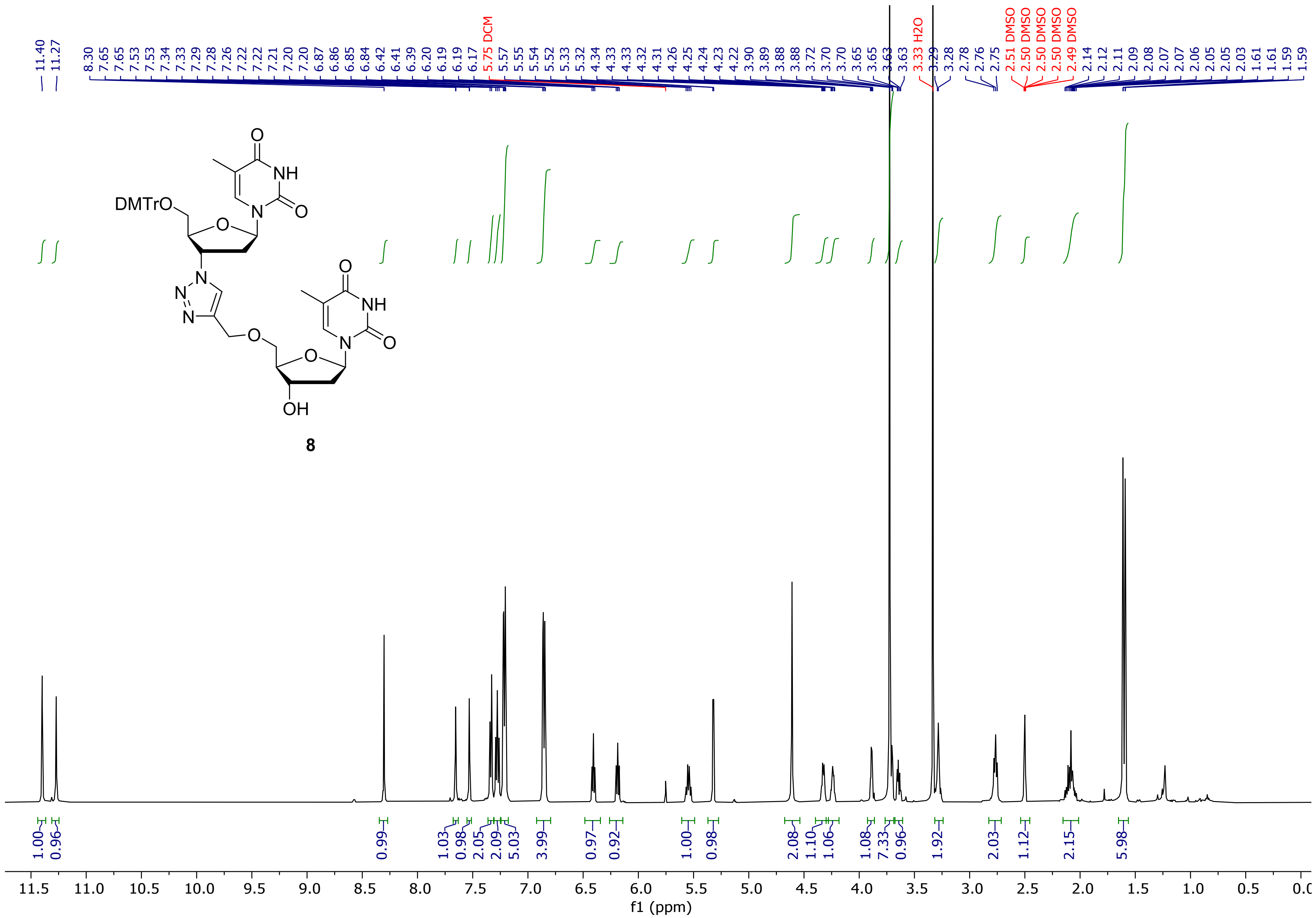

Figure S56. ^{1}H NMR (DMSO-d_6) of **8**.

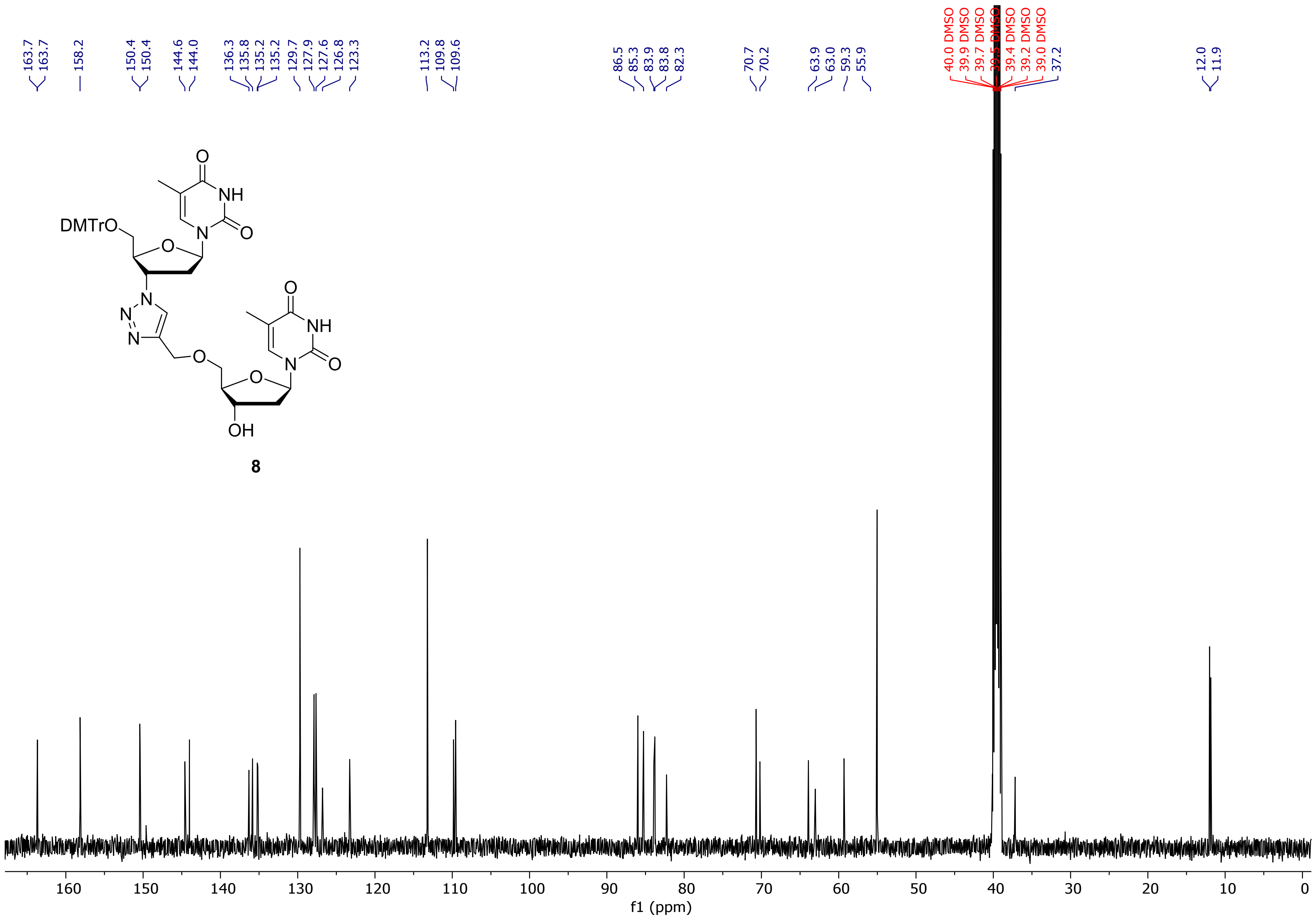

Figure S57. ^{13}C NMR (DMSO-d_6) of **8**.

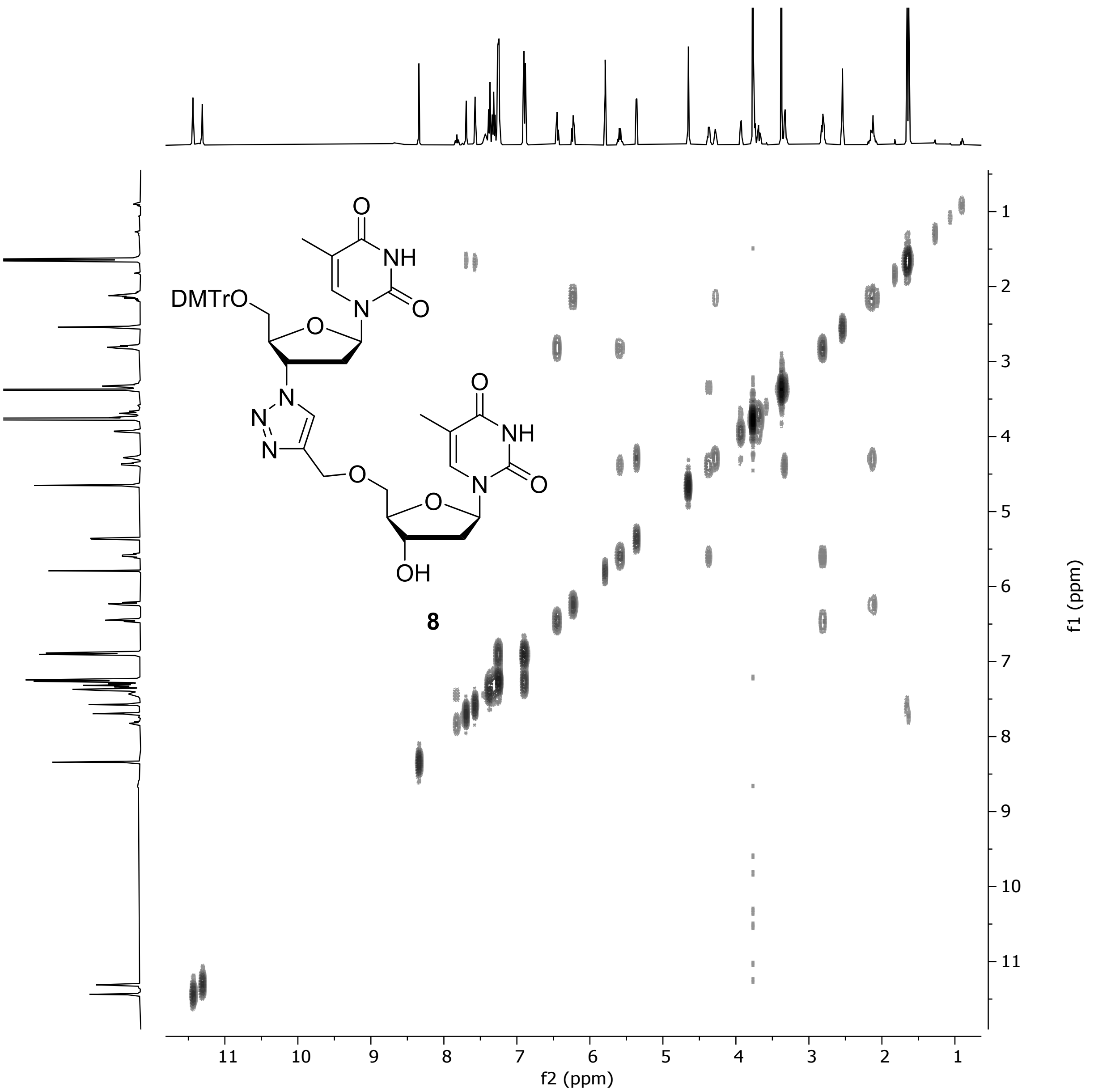

Figure S58. COSY NMR (DMSO-d_6) of **8**.

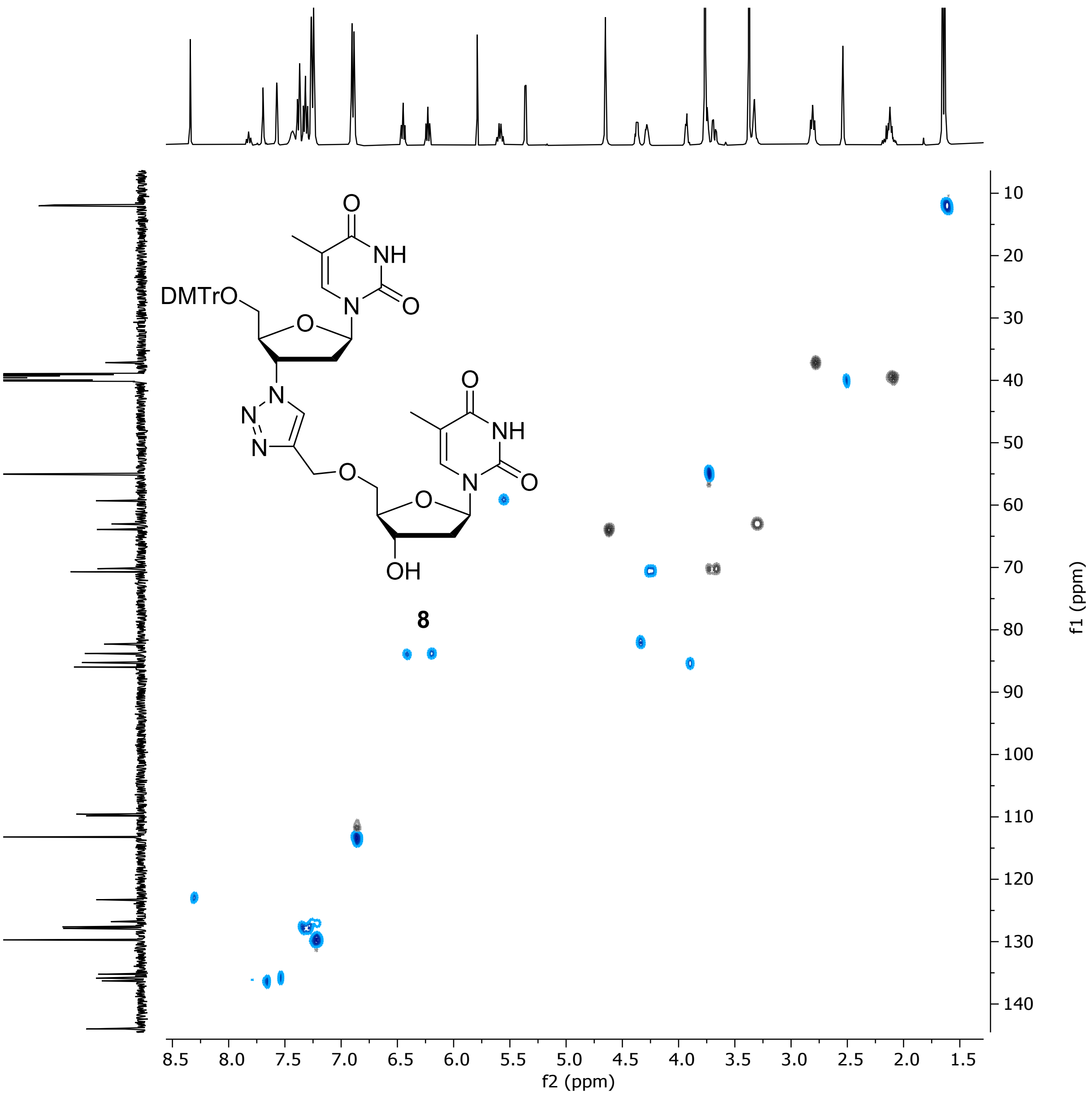

Figure S59. HSQC NMR (DMSO-d_6) of **8**.

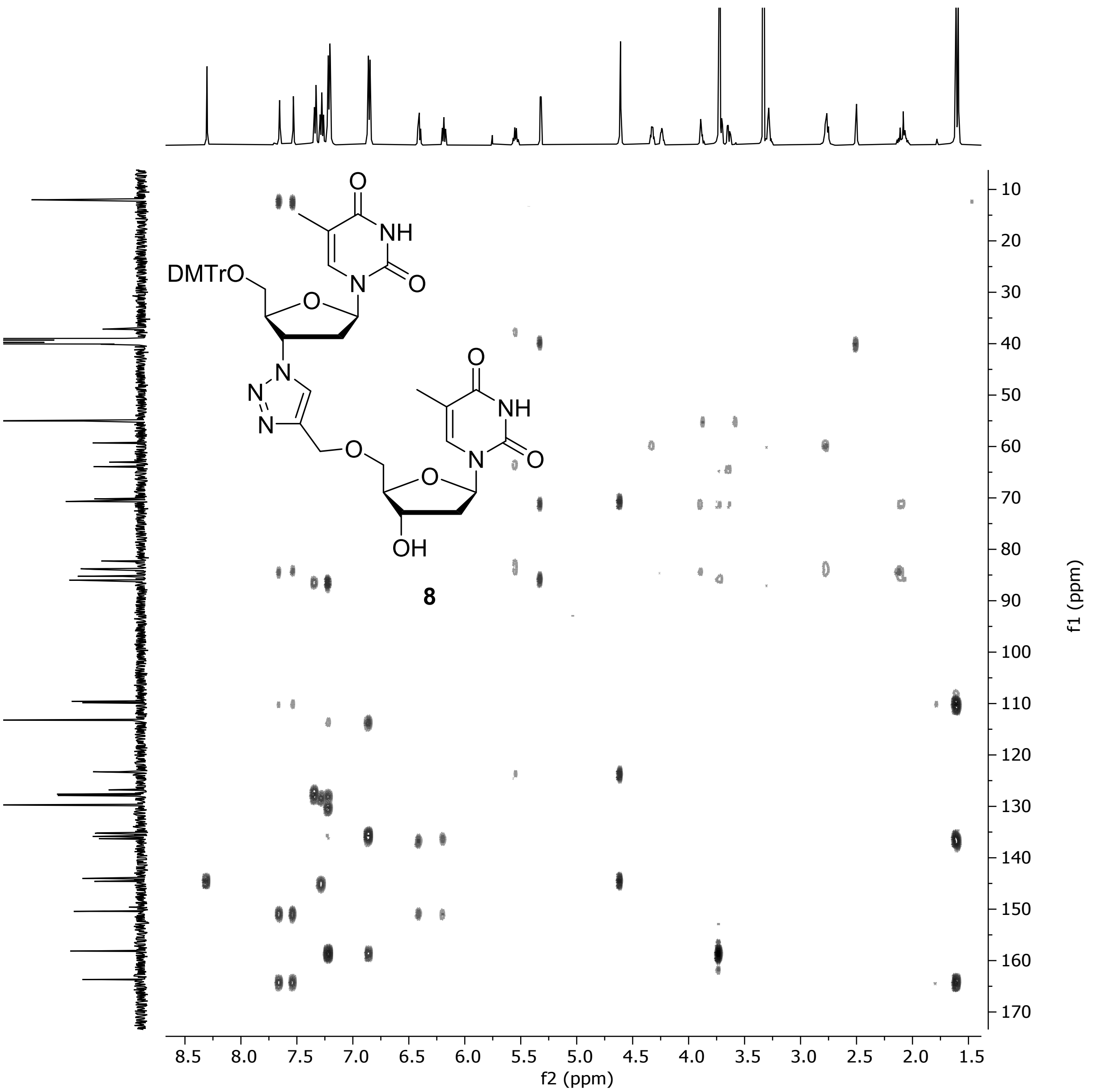

Figure S60. HMBC NMR (DMSO-d_6) of **8**.

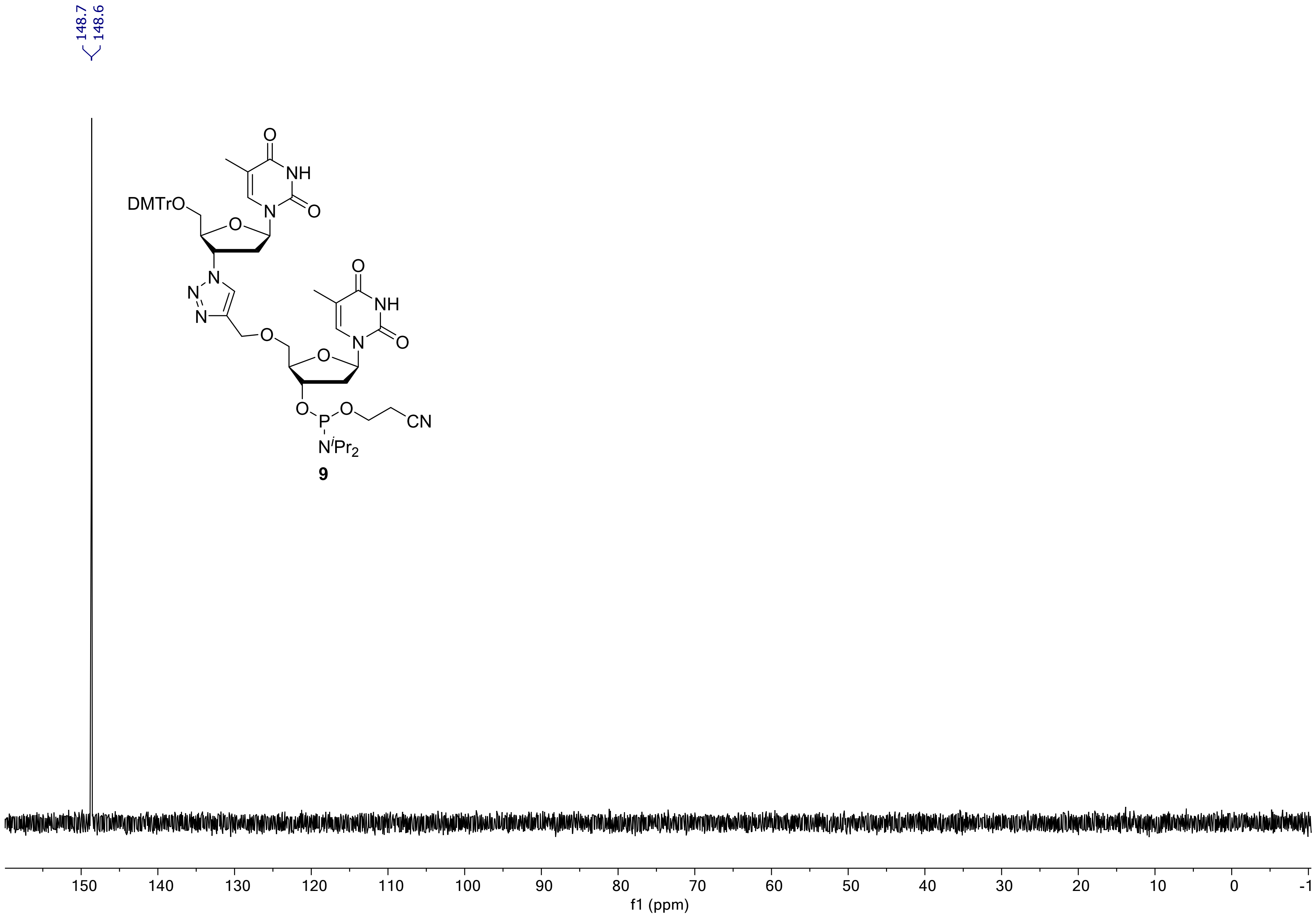

Figure S61. ^{31}P NMR ($CDCl_3$) of **9**.

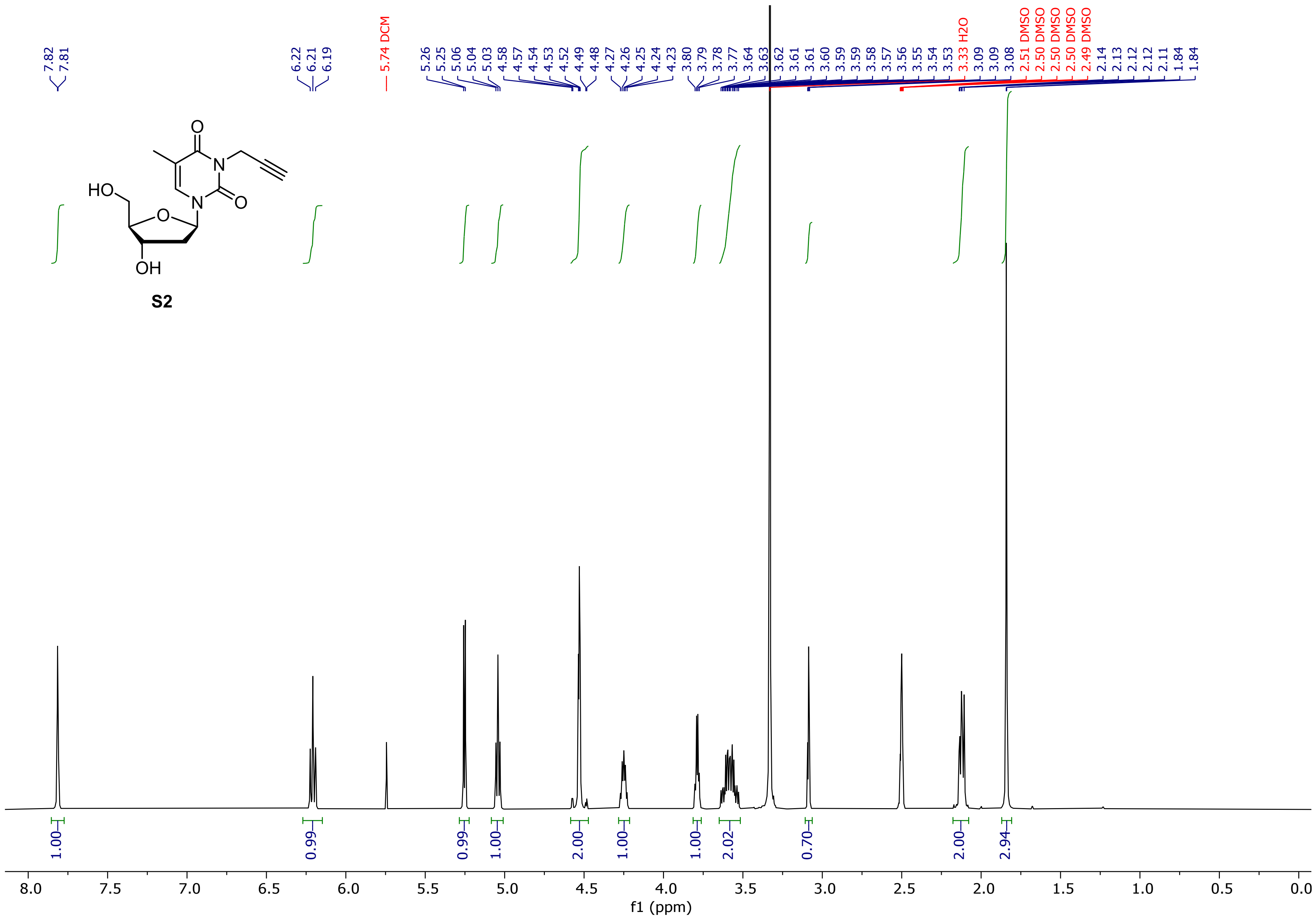

Figure S62. ^{1}H NMR (DMSO-d_6) of **S2**.

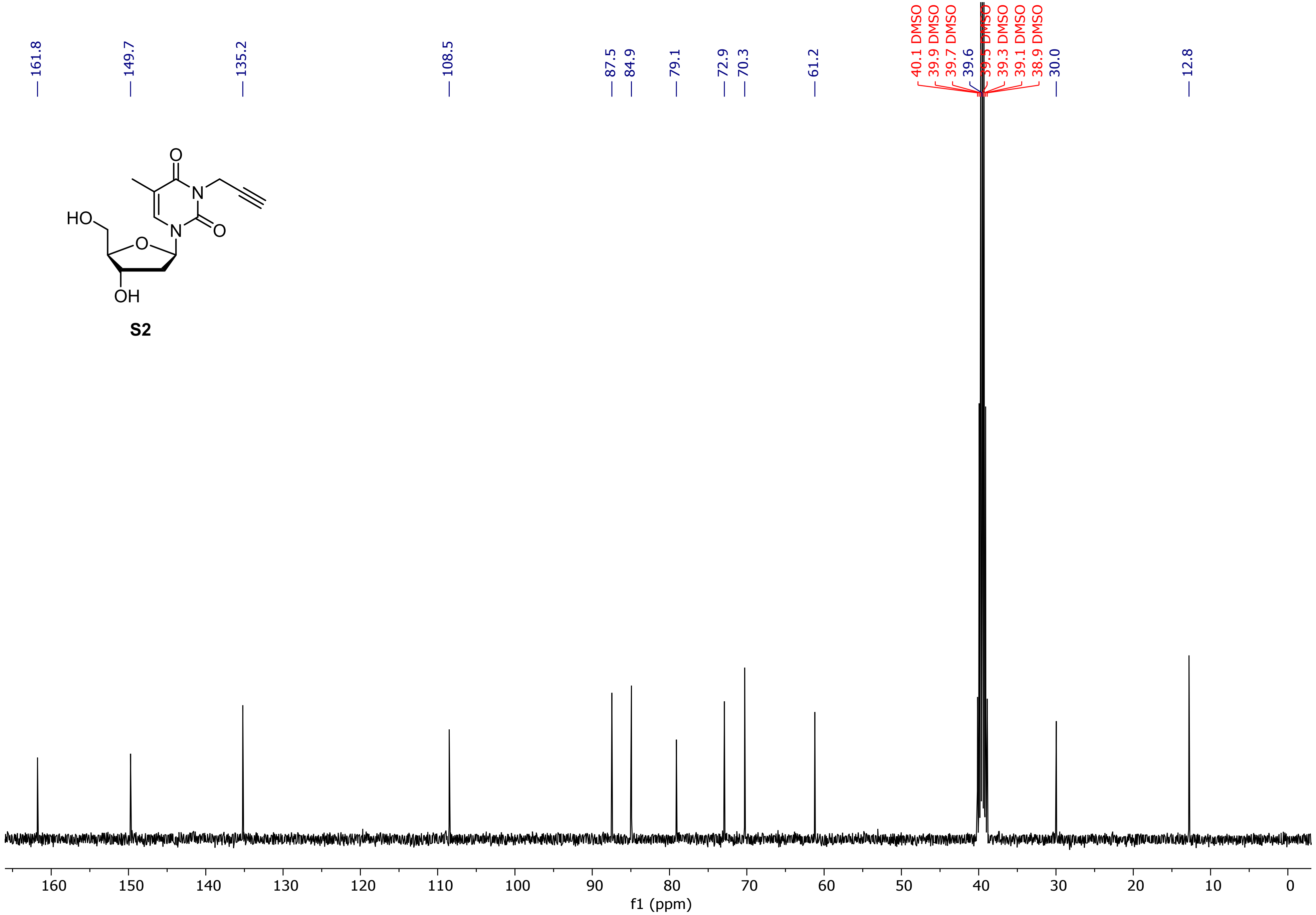

Figure S63. ^{13}C NMR (DMSO-d_6) of **S2**.

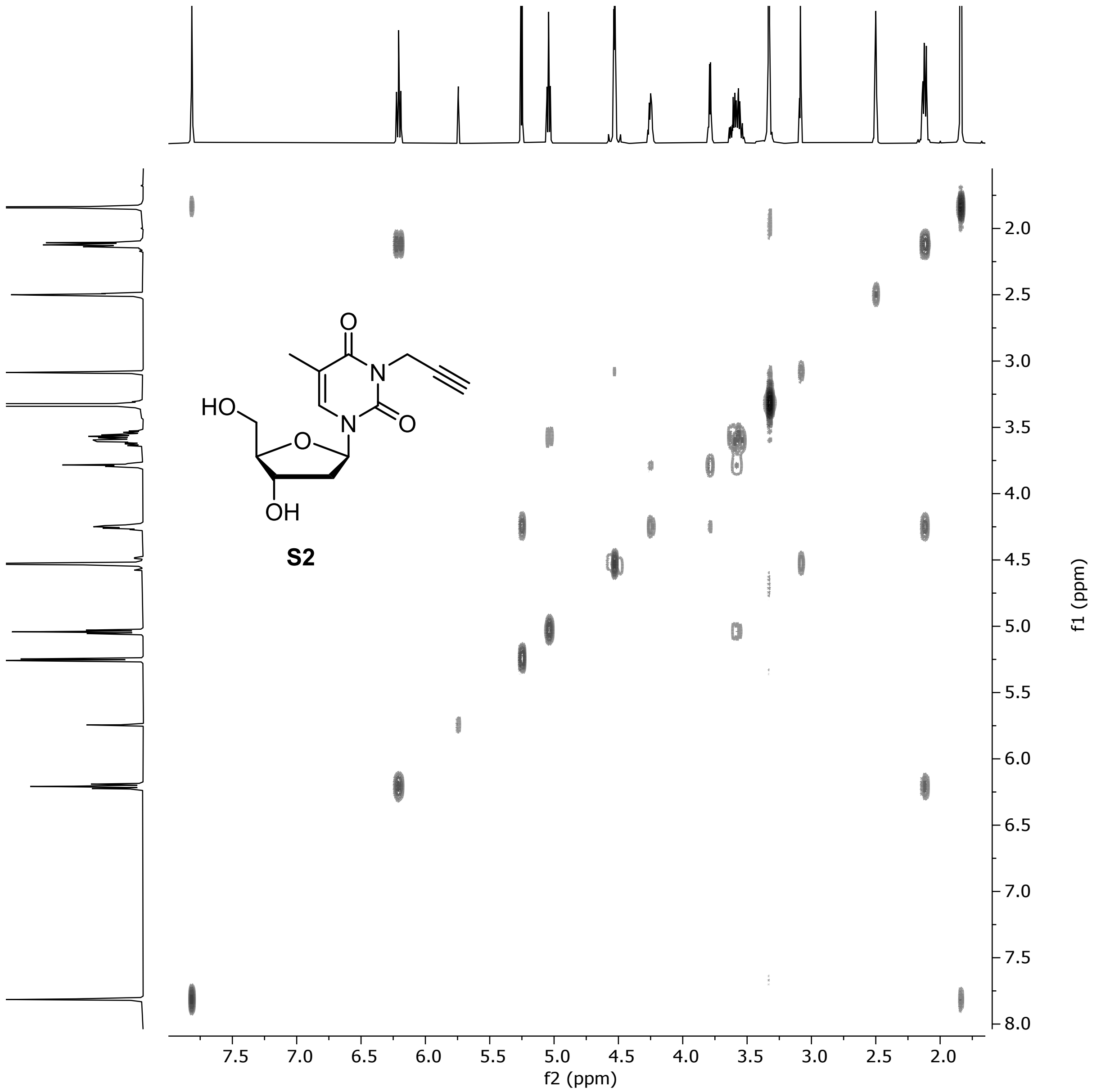

Figure S64. COSY NMR (DMSO-d_6) of **S2**.

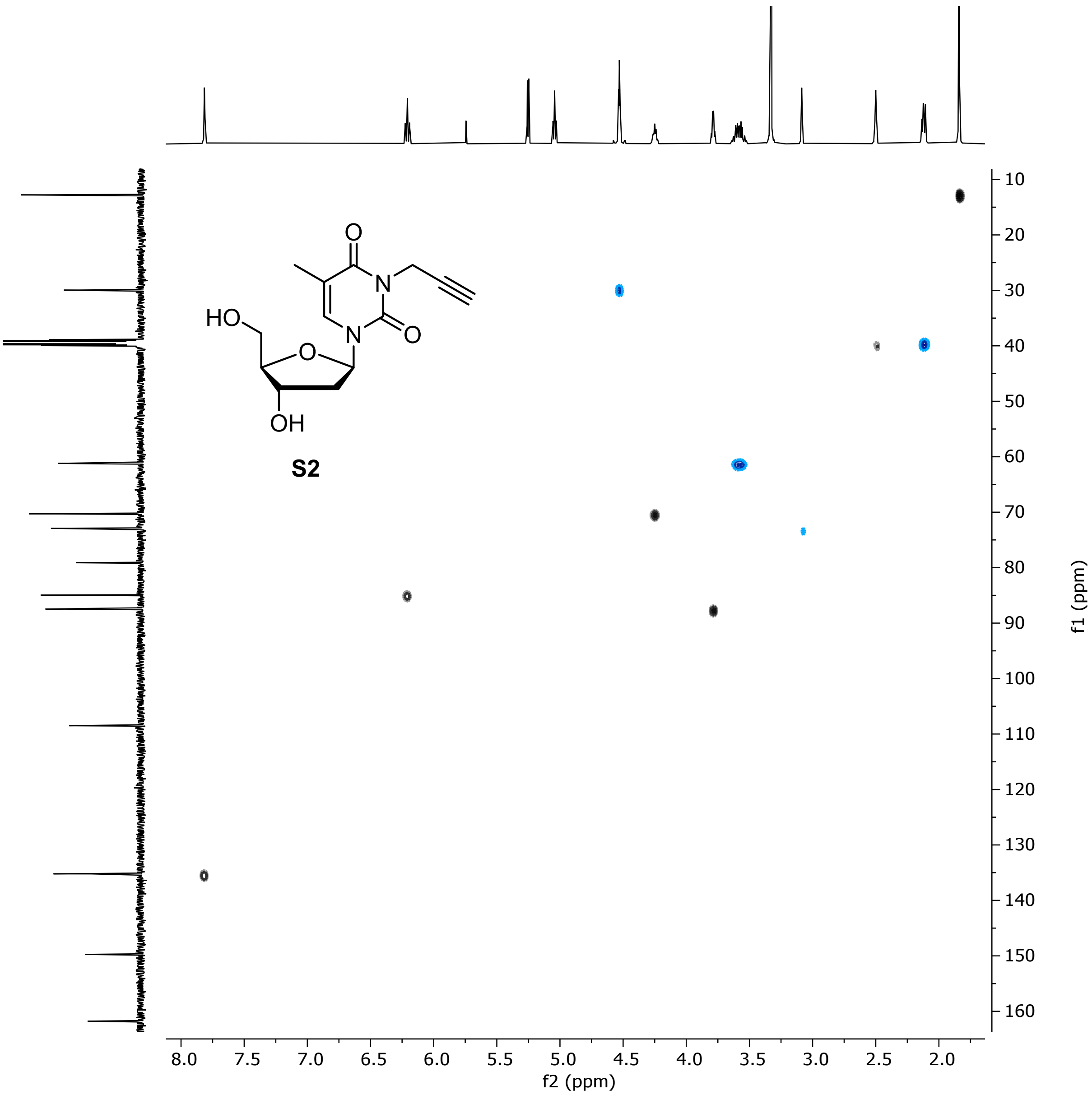

Figure S65. HSQC NMR (DMSO-d_6) of **S2**.

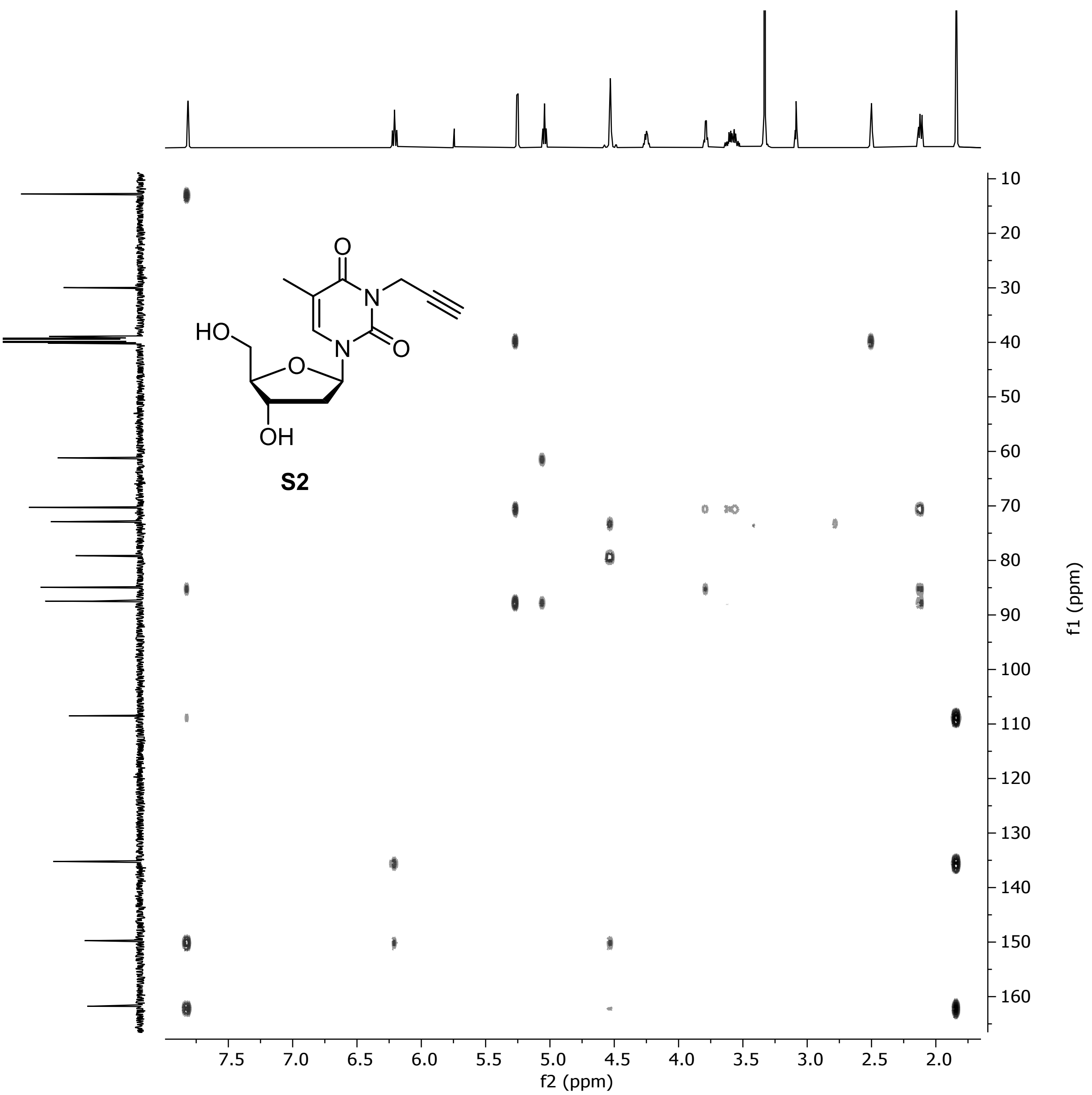

Figure S66. HMBC NMR (DMSO-d_6) of **S2**.

References

1 N. Kawagoe, Y. Kasori and T. Hasegawa, *Cellulose*, 2011, **18**, 83–93.

2 D. Baraniak, D. Baranowski, P. Ruszkowski and J. Boryski, *Nucleosides. Nucleotides Nucleic Acids*, 2019, **38**, 807–835.

3 T. Szabó and J. Stawiński, *Tetrahedron*, 1995, **51**, 4145–4160.
